# Supplementary material for: Stillbirth among women in nine states in India: rate and risk factors in study of 886,505 women from the annual health survey
Source: BMJ Open. 2018 Nov 8;8(11):e022583. doi: 10.1136/bmjopen-2018-022583 (PMC6231551; doi:10.1136/bmjopen-2018-022583)
Supplement: Supplementary file 1 [file bmjopen-2018-022583supp001.pdf]

Table-S1: Unadjusted and adjusted[1] odds ratios (OR) and 95%CI[2] for stillbirth by socioeconomic, health seeking behaviour, pre-existing medical conditions, and bio-demographic factors

Variables	Unadjusted Odds Ratios (95%CI)	Main model	Sensitivity analysis	
		Missing indicator Adjusted Odds Ratios (95%CI)	Complete case analysis Adjusted Odds Ratios (95%CI)	Multiple imputation Adjusted Odds Ratios (95%CI)
**Maternal Age (years)**				
15-19	1·52 (1·35-1·72)	1·76 (1·55-2·00)	1·58 (1·30-1·93)	1·79 (1·55 - 2·07)
20-24	1·17 (1·09-1·24)	1·29 (1·21-1·37)	1·21 (1·10-1·32)	1·29 (1·20 - 1·39)
25-29	1·00 (Ref)	1·00 (Ref)	1·00 (Ref)	1·00 (Ref)
30-34	1·20 (1·11-1·29)	1·05 (0·97-1·14)	1·13 (1·01-1·26)	1·07 (0·98 - 1·18)
35-39	1·43 (1·29-1·59)	1·16 (1·04-1·29)	1·18 (1·00-1·38)	1·18 (1·04 - 1·34)
40-45	1·63 (1·39-1·91)	1·29 (1·09-1·51)	1·42 (1·12-1·79)	1·37 (1·14 - 1·65)
45-49	1·24 (0·96-1·60)	1·04 (0·80-1·35)	1·05 (0·72-1·55)	1·05 (0·82 - 1·49)
**Place of Residence**				
Urban	1·00 (Ref)	1·00 (Ref)	1·00 (Ref)	1·00 (Ref)
Rural	1·35 (1·24-1·47)	1·27 (1·16 - 1·39)	1·31 (1·15-1·49)	1·25 (1·13 - 1·38)
**Religion**				
Hindu	1·00 (Ref)	1·00 (Ref)	1·00 (Ref)	1·00 (Ref)
Muslim	1·48 (1·39-1·58)	1·33 (1·25-1·43)	1·35 (1·22-1·49)	1·40 (1·29 - 1·51)
Christian	1·49 (1·25-1·78)	1·42 (1·19-1·70)	1·53 (1·25-1·86)	1·44 (1·20 - 1·73)
Others	0·87 (0·69-1·11)	1·06 (0·84-1·35)	0·85 (0·60-1·19)	1·09 (0·85 - 1·40)
**Social Group**				
Other	1·00 (Ref)	1·00 (Ref)	1·00 (Ref)	1·00 (Ref)
Schedule Caste	1·09 (1·02-1·16)	1·11 (1·04-1·19)	1·16 (1·05-1·28)	1·14 (1·05 - 1·23)
Schedule Tribe	0·68 (0·62-0·74)	0·74 (0·68-0·81)	0·67 (0·59-0·76)	0·71 (0·64 - 0·79)
**Maternal Education**				
Tertiary and above	1·00 (Ref)	1·00 (Ref)	1·00 (Ref)	1·00 (Ref)
Secondary	1·31 (1·08-1·60)	1·10 (0·90-1·34)	1·11 (0·84-1·47)	1·04 (0·84 - 1·30)
Primary and below	1·77 (1·48-2·12)	1·34 (1·10-1·62)	1·25 (0·95-1·64)	1·24 (1·00 - 1·53)
Illiterate	2·06 (1·72-2·47)	1·43 (1·17-1·74)	1·34 (1·02-1·77)	1·31 (1·06 - 1·62)
**Employment**				
In paid employment	1·00 (Ref)	1·00 (Ref)	1·00 (Ref)	1·00 (Ref)
Not in-paid employment	1·29 (1·20-1·38)	1·15 (1·07-1·24)	1·22 (1·10-1·36)	1·19 (1·09-1·29)
**Asset Index, Quintiles**				
5 "Highest"	1·00 (Ref)	1·00 (Ref)	1·00 (Ref)	1·00 (Ref)
4	1·26 (1·14-1·38)	1·34 (1·01-1·79)	1·58 (1·10-2·28)	1·32 (0·99-1·75)
3	1·41 (1·29-1·55)	1·91 (1·45-2·52)	2·13 (1·49-3·03)	1·81 (1·39-2·34)
2	1·46 (1·33-1·60)	2·45 (1·85-3·24)	2·64 (1·83-3·78)	2·23 (1·69-2·92)
1 "Lowest"	1·39 (1·26-1·52)	2·42 (1·82-3·21)	3·14 (2·20-4·47)	2·24 (1·71-2·94)
Missing	1·41 (1·27-1·56)	1·21 (0·91-1·62)	-	-

**Chewing Tobacco[3]**				
No	1·00 (Ref)	1·00 (Ref)	1·00 (Ref)	1·00 (Ref)
Yes	1·10 (1·01-1·19)	1·11 (1·02-1·21)	1·07 (0·97-1·19)	1·12 (1·00-1·25)
Missing	1·16 (1·09-1·24)	1·44 (1·29-1·59)	-	-
**Number of antenatal care visit**				
4 or more	1·00 (Ref)	1·00 (Ref)	1·00 (Ref)	1·00 (Ref)
Less than 4	1·19 (1·12-1·26)	1·08 (1·01-1·15)	1·11 (1·03-1·21)	1·06 (0·99-1·14)
Missing	1·53 (1·41-1·66)	1·36 (1·25-1·48)	-	-
**Timing of the first antenatal**				
**care visit**	1·00 (Ref)	-	-	-
Three or less	1·12 (1·06-1·19)	-	-	-
More than three	1·41 (1·31-1·52)	-	-	-
Missing				
**Place of delivery**				
Medical facility	1·00 (Ref)	-	-	-
Home	0·94 (0·89-0·99)	-	-	-
**Number of pregnancies**				
1	1·00 (Ref)	1·00 (Ref)	1·00 (Ref)	1·00 (Ref)
2-4	1·77 (1·63-1·93)	3·06 (2·42-3·86)	2·95 (2·20-3·94)	2·72 (2·18-3·38)
5 or more	2·60 (2·36-2·87)	4·98 (3·66-6·74)	5·35 (3·73-7·67)	4·30 (3·24-5·70)
Missing	1·21 (1·10-1·32)	1·40 (0·68-2·90)	-	-
**Sex of the baby**				
Female	1·00 (Ref)	1·00 (Ref)	1·00 (Ref)	1·00 (Ref)
Male	1·25 (1·19-1·32)	1·26 (1·20 - 1·33)	1·23 (1·14-1·32)	1·25 (1·18 - 1·32)
**Multiple gestations (index**				
**pregnancy)**	1·00 (Ref)	1·00 (Ref)	1·00 (Ref)	1·00 (Ref)
Singleton	2·03 (1·67-2·45)	1·77 (1·47 - 2·15)	1·90 (1·46-2·49)	1·84 (1·49 - 2·28)
Twin pregnancy				
**Mode of delivery**				
Spontaneous vaginal	1·00 (Ref)	1·00 (Ref)	1·00 (Ref)	1·00 (Ref)
Assisted vaginal	3·13 (2·76-3·55)	3·45 (3·02-3·93)	3·88 (3·25-4·63)	3·63 (3·16-4·18)
Caesarean section	1·53 (1·40-1·66)	1·73 (1·58-1·89)	1·75 (1·56-1·98)	1·58 (1·43-1·74)
**Any pregnancy complications**				
No	1·00 (Ref)	1·00 (Ref)	1·00 (Ref)	1·00 (Ref)
Yes	1·47 (1·38-1·56)	1·42 (1·33-1·51)	1·44 (1·33-1·56)	1·40 (1·31-1·49)
Missing	0·91 (0·84-0·98)	1·99 (1·00-3·94)	-	-

[1] Multivariable logistic regression, adjusting for the other variables in the model –except self-reported mental illness. The model also adjusts for the observed significant interaction between Gravidity and Asset Index.
[2] The 95% confidence intervals were calculated using linearized standard errors
[3] These variables reflect practice at the time of the survey
Results are weighted for design effects and non-response
