# Supplementary material for: Stillbirth among women in nine states in India: rate and risk factors in study of 886,505 women from the annual health survey
Source: BMJ Open. 2018 Nov 8;8(11):e022583. doi: 10.1136/bmjopen-2018-022583 (PMC6231551; doi:10.1136/bmjopen-2018-022583)
Supplement: Supplementary file 2 [file bmjopen-2018-022583supp002.pdf]

Table-S2: Sensitivity analysis - Unadjusted and adjusted[1] odds ratios (95% CI[2]) for stillbirth by pregnancy complications

Variable	Unadjusted Odds ratios (95%CI)	Main Model	Sensitivity analyses	
		Missing indicator method Adjusted Odds ratios (95%CI)	Complete case analysis[2] Adjusted Odds ratios (95%CI)	Multiple imputations[2] Adjusted Odds ratios (95%CI)
**Anemia**				
No	1 (Ref)	1 (Ref)	1 (Ref)	1 (Ref)
Yes	1·45 (1·36-1·54)	1·35 (1·27-1·43)	1·41 (1·30-1·52)	1·34 (1·26-1·42)
**Eclampsia**				
No	1 (Ref)	1 (Ref)	1 (Ref)	1 (Ref)
Yes	1·90 (1·73 -2·10)	1·79 (1·62 -1·97)	1·94 (1·72 -2·19)	1·79 (1·62 -1·98)
**Other hypertensive disorders**				
No	1 (Ref)	1 (Ref)	1 (Ref)	1 (Ref)
Yes	1·20 (1·05-1·36)	1·22 (1·07-1·38)	1·25 (1·07-1·46)	1·21 (1·06-1·38)
**Intra-partum Hemorrhage**				
No	1 (Ref)	1 (Ref)	1 (Ref)	1 (Ref)
Yes	2·97 (2·75 -3·21)	2·75 (2·54 -2·97)	2·73 (2·47 -3·02)	2·77 (2·56 -3·00)
**Ante-partum Hemorrhage**				
No	1 (Ref)	1 (Ref)	1 (Ref)	1 (Ref)
Yes	1·44 (1·25-1·67)	1·29 (1·11-1·50)	1·51 (1·39-1·64)	1·32 (1·14-1·53)
**Obstructed Labor**				
No	1 (Ref)	1 (Ref)	1 (Ref)	1 (Ref)
Yes	3·35 (3·13-3·58)	3·45 (3·19-3·74)	3·57 (3·24-3·94)	3·42 (3·16-3·70)
**Breech presentation**				
No	1 (Ref)	1 (Ref)	1 (Ref)	1 (Ref)
Yes	3·18 (2·88-3·50)	2·80 (2·51-3·12)	3·04 (2·65-3·47)	2·85 (2·56-3·18)
**Abnormal Fetal position**				
No	1 (Ref)	1 (Ref)	1 (Ref)	1 (Ref)
Yes	1·72 (1·54-1·92)	1·58 (1·40-1·77)	1·66 (1·43-1·91)	1·60 (1·43-1·80)

[1]Each multivariable logistic regression model adjusts for socioeconomic, health seeking behaviour, and bio-demographic characteristics identified to be significantly associated with Stillbirth in the previous model.

[2] Although the sub-population was restricted to women who had complete data on pregnancy-specific complication, the other variables adjusted for in the model had missing data, therefore sensitivity analyses were conducted.

Results are weighted for design effects and non-response
