# Supplementary material for: Stillbirth among women in nine states in India: rate and risk factors in study of 886,505 women from the annual health survey
Source: BMJ Open. 2018 Nov 8;8(11):e022583. doi: 10.1136/bmjopen-2018-022583 (PMC6231551; doi:10.1136/bmjopen-2018-022583)
Supplement: Author's manuscript [file bmjopen-2018-022583.draft_revisions.pdf]

BMJ Open

BMJ Open is committed to open peer review. As part of this commitment we make the peer review history of every article we publish publicly available.

When an article is published we post the peer reviewers' comments and the authors' responses online. We also post the versions of the paper that were used during peer review. These are the versions that the peer review comments apply to.

The versions of the paper that follow are the versions that were submitted during the peer review process. They are not the versions of record or the final published versions. They should not be cited or distributed as the published version of this manuscript.

BMJ Open is an open access journal and the full, final, typeset and author-corrected version of record of the manuscript is available on our site with no access controls, subscription charges or pay-per-view fees (http://bmjopen.bmj.com).

If you have any questions on BMJ Open's open peer review process please email editorial.bmjopen@bmj.com

BMJ Open

Stillbirth among women in nine states in India: Rate and risk factors in study of 886,505 women from the Annual Health Survey

Journal:	*BMJ Open*
Manuscript ID	bmjopen-2018-022583
Article Type:	Research
Date Submitted by the Author:	23-Feb-2018
Complete List of Authors:	Altijani , Noon; University of Oxford, Nuffield Department of Population Health<br>Carson, Claire; University of Oxford, National Perinatal Epidemiology Unit (NPEU); National Perinatal Epidemiology Unit (NPEU),<br>Choudhury , Saswati; FAA Medical College and Hospital, Department of Obstetrics and Gynaecology<br>Rani, Anjali; Banaras Hindu University Institute of Medical Sciences, Department of Obstetrics and Gynaecology<br>Sarma, Umesh; Srimanta Sankaradeva University of Health Sciences<br>Knight, Marian; National Perinatal Epidemiology Unit<br>Nair, Manisha; University of Oxford, NPEU, Nuffield Department of Population Health
Keywords:	Stillbirth, Risk factors, Pregnancy complications, India

SCHOLARONE™
Manuscripts

For peer review only

TITLE PAGE

Stillbirth among women in nine states in India: Rate and risk factors in study of 886,505 women from the Annual Health Survey

Authors

1. Noon Altijani (MSc)
   Nuffield Department of Population Health, University of Oxford
   Richard Doll Building, Old Road Campus, Headington, Oxford, OX3 7LF, UK
   Email: noon.altijani@gtc.ox.ac.uk

2. Claire Carson (PhD)
   National Perinatal Epidemiology Unit, Nuffield Department of Population Health, University of Oxford
   Richard Doll Building, Old Road Campus, Headington, Oxford, OX3 7LF, UK
   Email: claire.carson@npeu.ox.ac.uk

3. Saswati Sanyal Choudhury (MD)
   Department of Obstetrics and Gynaecology, FAA Medical College and Hospital, Barpeta, Barpeta-Hospital-Jania Road, Joti Gaon, Barpeta, Assam 781301, India
   Email: saswatischoudhury@gmail.com

4. Anjali Rani (MD)
   Department of Obstetrics and Gynaecology, Institute of Medical Sciences, Banaras Hindu University, Ajagara, Banaras Hindu University Campus, Varanasi, Uttar Pradesh 221005, India
   Email: anjalirani.imsbhu@gmail.com

5. Umesh C Sarma (MD)
   Srimanta Sankaradeva University of Health Sciences, Assam
   Narkashur Hilltop, Christian Basti Bhangagarh, Guwahati, Assam 781032, India
   Email: ssuhs_assam@yahoo.in

6. Marian Knight (DPhil)
   National Perinatal Epidemiology Unit, Nuffield Department of Population Health, University of Oxford
   Richard Doll Building, Old Road Campus, Headington, Oxford, OX3 7LF, UK.
   Email: marian.knight@npeu.ox.ac.uk

7. Manisha Nair (DPhil)
   National Perinatal Epidemiology Unit, Nuffield Department of Population Health, University of Oxford
   Richard Doll Building, Old Road Campus, Headington, Oxford, OX3 7LF, UK
   Email: manisha.nair@npeu.ox.ac.uk

Corresponding author

Manisha Nair
Senior Epidemiologist and Research Fellow,
National Perinatal Epidemiology Unit, NDPH, University of Oxford
Richard Doll Building, Old Road Campus, Headington, Oxford, OX3 7LF, UK
Email: manisha.nair@npeu.ox.ac.uk; Phone: +44-(0)1865-617820

For peer review only - http://bmjopen.bmj.com/site/about/guidelines.xhtml

ABSTRACT

Objectives: To assess the rate of stillbirth and associated risk factors across nine states in India.

Design: Secondary analysis of cross-sectional data from the Indian Annual Health Survey (2012-2013).

Setting: Nine states in India: Madhya Pradesh, Chattisgarh, Rajasthan, Uttarakhand, Jharkhand, Odisha, Bihar, Assam, Uttar Pradesh

Participants: 886,505 women, aged 15–49 years

Main outcome measures: Stillbirth rate with 95% Confidence Interval (CI). Adjusted odds ratio to examine the associations between stillbirth and (i) socioeconomic, behavioural, and bio-demographic risk factors (ii) complications in pregnancy (anaemia, eclampsia, other hypertensive disorders, antepartum and intrapartum haemorrhage, obstructed labour, breech presentation, abnormal fetal position).

Results: The overall rate of stillbirth was 10 per 1000 total births (95%CI: 9·8-10·3). Indicators of socioeconomic deprivation were strongly associated with an increase in stillbirth: rural residence (aOR:1·27,95%CI:1·16-1·39), female illiteracy (aOR:1·43,95%CI 1·17-1·74); low socioeconomic status (aOR:2·42, 95%CI:1·82-3·21); schedule caste background (aOR:1·11,95%CI:1·04-1·19), and woman not in paid employment (aOR:1·15,95%CI:1·07-1·24). Women from minority religious groups were at higher risk than the Hindu majority (Muslim (aOR:1·33,95%CI:1·25-1·43); Christian (aOR:1·42,95%CI:1·19-1·70)). While few women smoked (<1%), around 9% reported chewing tobacco, which was associated with an increased odds of stillbirth (aOR:1·11,95%CI:1·02-1·21). Adverse pregnancy and birth characteristics were also associated with stillbirth: antenatal care visits <4 (aOR:1·08,95%CI:1·01-1·15), maternal age <25 years (aOR:1·29,95%CI:1·21-1·37) and ≥35 years (aOR:1·16,95%CI:1·04-1·29), multigravida (aOR:3·06,95%CI:2·42-3·86), multiple pregnancy (aOR:1·77,95%CI:1·47-2·15), assisted delivery (aOR:3·45,95%CI:3·02-3·93), caesarean section (aOR:1·73,95%CI:1·58-1·89), as were pregnancy complications (aOR 1.42, 95%CI:1.33 – 1.51).

Conclusion: India is an emerging market economy experiencing a rapid health transition, yet these findings demonstrate the marked disparity in risk of stillbirth by women's socioeconomic status. Tobacco chewing, maternal and fetal complications were each found to be important modifiable risk factors. Targeting the 'at-risk' population identified here, improved recording of stillbirths and the introduction of local reviews would be important steps to reduce the high burden of stillbirths in India.

ARTICLE SUMMARY

Strengths and limitations of this study

- India has the highest number of stillbirths, globally. This study identifies the characteristics of high risk women and key risk factors in the Indian context using the largest available dataset

For peer review only - http://bmjopen.bmj.com/site/about/guidelines.xhtml

- The data were drawn from Indian Annual Health Survey (2012-2013), which sampled women from the nine states that account for 50% of the country's population, using methods designed to minimise selection bias – as a consequence, these findings are generalizable to the whole of India.
- This study analyses a large sample (>800,000 women), providing sufficient statistical power to conduct a robust examination of a wide range of risk factors
- However, retrospective data collection can lead to recall bias, and there is the potential for some under-reporting of stillbirth.

Key words: Stillbirth, Risk factors, Pregnancy complications, India

Word count: 2883

For peer review only

INTRODUCTION

Stillbirth is an important global health problem affecting over 7000 families every day and is associated with emotional, social and economic consequences.[1] In 2015 the stillbirth rate (SBR) was 18.4 per 1000 total births worldwide.[1] The progress in reducing stillbirth since 1990 has been slower than reductions in neonatal and under-five child mortality.[2] Currently, 98% of stillbirths occur in low-to-middle-income countries (LMICs)[1] and India has the highest number of stillbirths, with an estimated 592,100 deaths per year[3], and a World Health Organisation (WHO) estimated rate of 22 per 1000 total births. The Government of India has developed an Indian Newborn Action Plan (INAP) with a goal to reduce stillbirths to <10 per 1,000 births by 2030".[4] A modest reduction in India's SBR would translate into thousands of lives saved.

While previous studies have examined the immediate pregnancy-related risk factors for stillbirth such as infections during pregnancy and hypertensive disorders,[1 5] knowledge about distal risk factors such as socioeconomic, lifestyle related and co-morbidities is limited.[1] The Indian government recognises the need to improve pregnancy care and institutional delivery among disadvantaged socioeconomic groups who have a higher risk of maternal and fetal death.[6] Since 2005, the government has made several efforts including cash assistance and dedicated services through community health workers with a stronger focus in the states with poor health and development indicators.[6] The objective of this study was to understand the current disparities in the risk of stillbirth in these 'high focus' states by examining the association between socioeconomic, bio-demographic, behavioural, and pregnancy-specific risk factors and stillbirth in nine states in India.

METHODS

We conducted an analysis of secondary data from India's Annual Health Survey (2012-13). The survey covers nine states (Madhya Pradesh, Chattisgarh, Rajasthan, Uttarakhand, Jharkhand, Odisha, Bihar, Assam, Uttar Pradesh) which account for about 50% of the country's population.[7] A total of 886,505 women for whom information about the outcome of their last pregnancy (live birth or stillbirth) was available and whose pregnancy lasted more than seven completed months (~28 weeks' gestation) were included. Figure-1 illustrates how the sample for this study was derived.

Based on the outcome of last pregnancy, women were divided into two groups, those with a livebirth and those with a stillbirth. Potential risk factors for stillbirth were grouped as:

For peer review only - http://bmjopen.bmj.com/site/about/guidelines.xhtml

socioeconomic, behavioural, and bio-demographic based on a review of published literature. The description of variables included in each group is provided in Table-1. A directed acyclic graph was used to construct a theoretical framework of relationships between the aforementioned risk factors and stillbirth (Figure-2). In addition, in a sub-sample of the population (n=668,892), we examined the association of stillbirth with the following self-reported problems/ complications during pregnancy: anaemia, eclampsia, other hypertensive disorders, antepartum haemorrhage, intrapartum haemorrhage, abnormal fetal position, breech presentation, and obstructed labour.

Table 1: Description of the variables used to examine the risk factors for stillbirth

**Risk factors**	**Description of the variables**
**Proximal or Bio-Demographic factors**	
**Fetal factors**	
Sex	Sex of the foetus was coded as either female or male
Multiple pregnancy	Number of foetuses: Women were categorised as singleton (one foetus) or multiple pregnancy (twins or higher-order multiple gestations)
**Maternal factors**	
Maternal age	Maternal age at the time of the survey was divided into five year age bands: <20, 20-24, 25-29, 30-34, 35-39, 40-44, 45 and above
Gravidity	Gravidity (number of pregnancies) was used as a proxy for parity (number of deliveries) for which information was not available· Gravidity was categorised as "first" if the index pregnancy was the first pregnancy, "second - fourth", and "five or more"·
Any complication during pregnancy	Women who reported to have any of the following complications during their index pregnancy we coded as 'yes', otherwise 'no': anaemia, eclampsia, other hypertensive disorders, antepartum haemorrhage, intrapartum haemorrhage, abnormal fetal position, breech presentation, and obstructed labour
Mode of delivery	This was categorised into vaginal delivery, assisted vaginal delivery and Caesarean section
**Intermediate factors or behavioural factors**	
**Lifestyle factors**	
Smoking tobacco	Women were asked about smoking and chewing tobacco during the interview; current practice was coded as "yes", while women who never practiced or no longer practiced were coded as "no"
Chewing tobacco	
**Health seeking behaviour**	
Antenatal care visits	Number of antenatal care (ANC) visits was categorised as '≥4 visits' (which is recommended as adequate by the WHO), and '<4 visits'
Timing of first ANC visit	Timing of the first ANC visit was categorised as 'visit in the first trimester (or first three months), which is recommended as adequate by the WHO, and 'after first three months'
Place of delivery	Place of delivery was grouped as "medical facility" or "home"
**Distal or Socioeconomic factors**	
**Social factors**	
Religion	Women were categorised into the following religious groups: 'Hindu',

For peer review only - http://bmjopen.bmj.com/site/about/guidelines.xhtml

	'Muslim', 'Christian', and 'Others' which included several groups with small numbers such as Sikh, Buddhists, etc
Place of Residence	Place of residence was grouped into urban and rural
Social group	Women were categorised into the following social groups: 'Schedule caste (SC)', 'Schedule tribe (ST)' and 'Others'<br>○ SC and ST are officially designated groups of historically disadvantaged populations in India<br>○ 'Others' included the general social class and other backward classes
**Economic factors**	
Education	Maternal education at the time of the survey was categorised into: illiterate, primary school or below, secondary school, tertiary and above
Occupation	Based on occupational status, women were grouped as 'being in paid employment' or 'not in paid employment'
Economic status	Asset index was used to measure the economic status of the participants· Asset index scores were calculated and the study sample was divided into quintiles ranging from the lowest (quintile-1) to highest (quintile-5) socioeconomic status

Statistical analysis

Overall and state-specific rates of stillbirth per 1000 total births with 95% confidence intervals (CIs) were calculated in the study population. The characteristics of women who had a stillbirth were compared with those who had a livebirth. A univariable logistic regression analysis was performed to investigate the association between each potential risk factor and stillbirth. Maternal age was tested for linearity which showed the presence of a non-linear association with stillbirth (Figure-3); it was therefore used as a categorical variable. A multivariable model was built using a step-wise forward regression, including risk factors that were statistically significantly associated with stillbirth during univariable analysis (based on a Wald p-value of <0.05). The order in which the variables were added to the model, distal followed by intermediate then proximal factors, was informed by the theoretical framework (Figure-2). Three variables, 'smoking tobacco', 'place of delivery' and 'timing of first ANC visit' were not statistically significantly associated with stillbirth at $p<0.05$ during the model building process, and were therefore dropped from the final models.

Calculated pairwise correlation coefficients did not show any significant collinearity between the risk factors. The following interactions were identified a priori and tested for significance using Wald test, which is a method of choice for survey-weighted data: Asset index and gravidity, place of residence and place of delivery. We found a significant interaction between 'Gravidity' (number of pregnancies) and 'Asset index'. The final regression model

For peer review only - http://bmjopen.bmj.com/site/about/guidelines.xhtml

was adjusted for this by fitting an interaction term. We conducted further analysis to examine the association between gravidity stratified by the quintiles of the Asset index.

In a sub-sample of the study population with complete data on complications during pregnancy and medical co-morbidities, we conducted univariable logistic regression analysis to investigate their individual association with stillbirth. Statistically significant associations were further examined using multivariable logistic regression models that adjusted for socioeconomic, behavioural and bio-demographic risk factors found to be significantly associated with stillbirth.

Missingness was investigated and data were assumed to be 'Missing at Random'. Three methods were used to address bias due to missing data; missing indicator method, complete case analysis, and multiple imputations.[8] The 'missing indicator' model in which missing data were grouped as a separate category was used as the final model. However, to maintain model stability, for variables that had <1% missing data, a separate category for 'missing' was not generated. All analyses accounted for the stratified, clustered nature of the data and were conducted using the survey command (svy) in Stata V.13.1, SE (StataCorp, College Station, Texas, USA). The proportions, means and confidence intervals presented here are weighted for design effects and non-response.

Study power

The fixed sample had more than 90% power to detect an OR of ≥1.11 or ≤0.91 associated with stillbirth at p<0.05 (two-tailed) for the risk factor with the highest prevalence (83% for rural residence) and an OR of ≥1.39 or ≤0.67 for the risk factor with the lowest prevalence in the study population (current smoker 0.8%).

Patient and Public Involvement

This is not applicable since this was a secondary analysis of anonymous survey data.

RESULTS

Rate of Stillbirth

Of the 886,505 women included in the analyses 8,429 reported a stillbirth, giving an overall rate of 10 stillbirths (95%CI 9.8-10.3) per 1000 total births. The rates of stillbirth per state are shown in Table-2 and Figure-4.

Table 2: Number and rate of stillbirths in the study population in nine states in India

State	Stillbirths	Total births	Stillbirth rate per 1000 total birth (95% CI)

For peer review only - http://bmjopen.bmj.com/site/about/guidelines.xhtml

Assam	856	64 841	12·8 (11·8-13·9)
Bihar	2 833	250 609	11·3 (10·9-11·8)
Chhattisgarh	476	98 220	5·0 (4·5-5·5)
Jharkhand	657	60 153	10·6 (9·7-11·6)
Madhya Pradesh	615	145 552	4·2 (3·8 - 4·6)
Odisha	534	46 162	10·9 (9·9-12·0)
Rajasthan	413	52 537	7·1 (6·4-8·0)
Uttar Pradesh	1 813	131 324	14·8 (14·1-15·6)
Uttarakhand	233	37 485	7·7 (6·5-9·2)
Overall	8 430	886 505	10·0 (9·8-10·3)

Frequencies are unweighted (true counts). Rates are weighted for design effects and non-response

Association between socioeconomic, behavioural, and bio-demographic characteristics and stillbirth

The median age for women in the study population was 26 years (Interquartile range 23-30). The frequency, proportions and association with stillbirth (unadjusted and adjusted OR) for socioeconomic, behavioural and bio-demographic variables are shown in Table 3, and pregnancy-related variables in Table 4. After adjusting for other risk factors, all examined socioeconomic factors were found to be significantly associated with stillbirth. Women living in rural settings had 27% higher odds of stillbirth compared with urban settings. Belonging to a Schedule caste social group was associated with 11% higher odds compared with the reference 'other' social group, but 26% lower odds of stillbirth for women belonging to the Schedule tribe group. Compared with Hindus, belonging to Muslim and Christian religious groups was associated with increased odds of stillbirth. Compared with women with university education or higher, women with no school education had 43% higher odds. Women not in paid employment were 15% more likely to have a stillborn baby compared with women in paid employment.

Chewing tobacco was associated with 11% higher odds of stillbirth after adjusting for other risk factors in the study population. Health seeking behaviours were significantly associated with stillbirth. Women attending <4 antenatal care (ANC) visits had 8% higher odds compared with women who had ≥4 ANC visits.

All examined demographic and pregnancy-related factors were also found to be significantly associated with stillbirth in the study population. After full adjustment, the non-linear (U-shaped) association between mother's age and stillbirth persisted. Multiple pregnancies were associated with 77% higher odds of stillbirth compared with singleton. Pregnancies with a male fetus had 26% higher odds compared with a female fetus. Women who had an assisted delivery or caesarean section were more likely to report a stillbirth compared with women who had a normal delivery. However, the association between stillbirth and caesarean section was no longer significant at $p<0.05$ and the aOR for assisted delivery was

For peer review only - http://bmjopen.bmj.com/site/about/guidelines.xhtml

significantly attenuated in the model that examined the association between obstructed labour and stillbirth (discussed below).

We found a significant statistical interaction between ‘gravidity’ and ’Asset index’. A stratified analysis showed that multigravida women had higher odds of stillbirth and this association was substantially stronger for women who belonged to the higher Asset Index quintiles (Table-5). The results of sensitivity analyses (complete case analysis and multiple imputations) were not materially different from the ‘missing indicator’ model (Supplementary Table-S1).

For peer review only

For peer review only - http://bmjopen.bmj.com/site/about/guidelines.xhtml

Table 3: The Association between stillbirth and socioeconomic behaviouraland bio-demographic factors: frequencies, unadjusted and adjusted Odds Ratios

Variables	Total births N=886 505 Frequency (%)	Stillbirths N= 8 429 Frequency (%)	Live births, N= 878 076 Frequency (%)	Unadjusted Odds Ratios (95%CI[2])	Adjusted Odds Ratios[1] (95%CI[2])
**Maternal Age (years)**					
15-19	33 482 (3·7)	436 (4·9)	33 046 (3·7)	1·52 (1·35-1·72)	1·76 (1·55-2·00)
20-24	324 130 (36·0)	3 079 (36·2)	321 051 (36·0)	1·17 (1·09-1·24)	1·29 (1·21-1·37)
25-29	301 330 (33·7)	2 546 (29·2)	298 784 (33·7)	1·00 (Ref)	1·00 (Ref)
30-34	143 529 (16·6)	1 400 (17·1)	142 129 (16·6)	1·20 (1·11-1·29)	1·05 (0·97-1·14)
35-39	55 893 (6·6)	643 (8·2)	55 250 (6·6)	1·43 (1·29-1·59)	1·16 (1·04-1·29)
40-45	20 006 (2·4)	244 (3·4)	19 762 (2·4)	1·63 (1·39-1·91)	1·29 (1·09-1·51)
45-49	8 135 (1·0)	81 (1·1)	8 054 (1·0)	1·24 (0·96-1·60)	1·04 (0·80-1·35)
**Place of Residence**					
Urban	123 099 (16·4)	874 (12·7)	122 225 (16·4)	1·00 (Ref)	1·00 (Ref)
Rural	763 406 (83·7)	7 555 (87·4)	755 851 (83·6)	1·35 (1·24-1·47)	1·27 (1·16 - 1·39)
**Religion**					
Hindu	734 134 (81·5)	6 522 (75·3)	727 612 (81·5)	1·00 (Ref)	1·00 (Ref)
Muslim	125 723 (15·9)	1 619 (21·6)	124 104 (15·8)	1·48 (1·39-1·58)	1·33 (1·25-1·43)
Christian	14 914 (1·8)	178 (2·4)	14 736 (1·8)	1·49 (1·25-1·78)	1·42 (1·19-1·70)
Others	11 734 (0·9)	110 (0·7)	11 624 (0·9)	0·87 (0·69-1·11)	1·06 (0·84-1·35)
**Social Group**					
Other	598 477 (68·4)	5 868 (70·0)	592 609 (68·4)	1·00 (Ref)	1·00 (Ref)
Schedule Caste	161 273 (19·2)	1 644 (21·4)	159 629 (19·2)	1·09 (1·02-1·16)	1·11 (1·04-1·19)
Schedule Tribe	126 755 (12·4)	917 (8·7)	125 838 (12·5)	0·68 (0·62-0·74)	0·74 (0·68-0·81)
**Maternal Education**					
Tertiary and above	36 617 (3·9)	188 (2·2)	36 429 (4·0)	1·00 (Ref)	1·00 (Ref)
Secondary	110 458 (11·3)	786 (8·2)	109 672 (11·3)	1·31 (1·08-1·60)	1·10 (0·90-1·34)
Primary and below	364 809 (39·1)	3 413 (38·1)	361 396 (39·1)	1·77 (1·48-2·12)	1·34 (1·10-1·62)
Illiterate	374 621 (45·7)	4 042 (51·6)	370 579 (45·6)	2·06 (1·72-2·47)	1·43 (1·17-1·74)
**Employment**					
In paid employment	154 177 (16·4)	1 273 (13·2)	152 904 (16·4)	1·00 (Ref)	1·00 (Ref)
Not in-paid employment	732 328 (83·6)	7 156 (86·8)	725 172 (83·6)	1·29 (1·20-1·38)	1·15 (1·07-1·24)
**Asset Index, Quintiles**					
5 "Highest"	159 957 (17·9)	1 133 (13·6)	158 824 (17·9)	1·00 (Ref)	1·00 (Ref)
4	160 335 (17·6)	1 422 (16·9)	158 913 (17·6)	1·26 (1·14-1·38)	1·34 (1·01-1·79)
3	159 567 (18·0)	1 606 (19·3)	157 961 (18·0)	1·41 (1·29-1·55)	1·91 (1·45-2·52)
2	157 506 (17·9)	1 654 (19·8)	155 852 (17·8)	1·46 (1·33-1·60)	2·45 (1·85-3·24)
1 "Lowest"	155 283 (18·0)	1 626 (18·9)	153 657 (17·9)	1·39 (1·26-1·52)	2·42 (1·82-3·21)
Missing	93 857 (10·7)	988 (11·5)	92 869 (10·7)	1·41 (1·27-1·56)	1·21 (0·91-1·62)
**Current Smoking[3]**				Add this	n/a
No	725 560 (82·0)	6 670 (79·6)	718 890 (82·0)		
Yes	6 553 (0·8)	80 (1·2)	6 473 (0·8)		
Missing	154 392 (17·2)	1 679(19·3)	152 713 (17·2)		

For peer review only - http://bmjopen.bmj.com/site/about/guidelines.xhtml

**Chewing Tobacco**[3]					
No	635 393 (74·1)	5 743 (71·5)	629 650 (74·1)	1·00 (Ref)	1·00 (Ref)
Yes	96 806 (8·7)	1 010 (9·3)	95 796 (8·7)	1·10 (1·01-1·19)	1·11 (1·02-1·21)
Missing	154 306 (17·2)	1 676 (19·2)	152 630 (17·2)	1·16 (1·09-1·24)	1·44 (1·29-1·59)

[1] Multivariable logistic regression, adjusting for the other variables in the model –except self-reported mental illness. The model also adjusts for the observed significant interaction between Gravidity and Asset Index.
[2] The 95% confidence intervals were calculated using linearized standard errors
[3] These variables reflect practice at the time of the survey
Frequencies are true counts, % and OR are weighted for design effects and non-response

For peer review only

For peer review only - http://bmjopen.bmj.com/site/about/guidelines.xhtml

Table 4: The Association between stillbirth and pregnancy-related factors: frequencies, unadjusted and adjusted Odds Ratios

Variables	Total births N=886 505 Frequency (%)	Stillbirths N= 8 429 Frequency (%)	Live births N= 878 076 Frequency (%)	Unadjusted Odds Ratios (95%CI[2])	Adjusted Odds Ratios[1] (95%CI[2])
**Number of antenatal care visit**					
4 or more	269 148 (28·9)	2 222 (24·6)	266 926 (29·0)	1·00 (Ref)	1·00 (Ref)
Less than 4	518 288 (58·7)	4 939 (59·3)	513 349 (58·6)	1·19 (1·12-1·26)	1·08 (1·01-1·15)
Missing	99 069 (12·4)	1 268 (16·1)	97 801 (12·4)	1·53 (1·41-1·66)	1·36 (1·25-1·48)
**Timing of the first antenatal care visit**					
Three or less	534 118 (58·6)	4 678 (54·0)	529 440 (58·7)	1·00 (Ref)	-
More than three	253 460 (28·9)	2 483 (29·9)	250 977 (28·9)	1·12 (1·06-1·19)	-
Missing	98 927 (12·4)	1 268 (16·1)	97 659 (12·4)	1·41 (1·31-1·52)	-
**Place of delivery**					
Medical facility	539 704 (60·5)	5 176 (61·9)	534 528 (60·5)	1·00 (Ref)	-
Home	346 801 (39·5)	3 253 (38·1)	343 548 (39·5)	0·94 (0·89-0·99)	-
**Number of pregnancies**					
1	158 886 (16·9)	957 (10·6)	157 929 (16·9)	1·00 (Ref)	1·00 (Ref)
2-4	414 938 (46·8)	4 409 (51·8)	410 529 (46·7)	1·77 (1·63-1·93)	3·06 (2·42-3·86)
5 or more	93 458 (11·7)	1 404 (19·0)	92 024 (11·7)	2·60 (2·36-2·87)	4·98 (3·66-6·74)
Missing	219 253 (24·7)	1 659 (18·6)	217 594 (24·7)	1·21 (1·10-1·32)	1·40 (0·68-2·90)
**Sex of the baby**					
Female	414 913 (46·6)	3 468 (41·2)	411 445 (46·7)	1·00 (Ref)	1·00 (Ref)
Male	471 592 (53·4)	4 961 (58·9)	466 631 (53·3)	1·25 (1·19-1·32)	1·26 (1·20 - 1·33)
**Multiple gestations (index pregnancy)**					
Singleton	878 556 (99·1)	8 267 (98·2)	870 289 (99·1)	1·00 (Ref)	1·00 (Ref)
Twin pregnancy	7 949 (0·9)	162 (1·8)	7 787 (0·9)	2·03 (1·67-2·45)	1·77 (1·47 - 2·15)
**Mode of delivery**					
Spontaneous vaginal	805 430 (90·7)	7 146 (84·3)	798 284 (90·8)	1·00 (Ref)	1·00 (Ref)
Assisted vaginal	14 909 (1·8)	390 (5·0)	14 519 (1·7)	3·13 (2·76-3·55)	3·45 (3·02-3·93)
Caesarean section	66 166 (7·6)	893 (10·7)	65 273 (7·5)	1·53 (1·40-1·66)	1·73 (1·58-1·89)
**Any pregnancy complications**					
No	250 099 (28·1)	2 000 (23·5)	250 099 (28·1)	1·00 (Ref)	1·00 (Ref)
Yes	418 856 (47·5)	4 779 (58·0)	414 077 (47·4)	1·47 (1·38-1·56)	1·42 (1·33-1·51)
Missing	217 550 (24·5)	1 650 (18·5)	215 900 (24·5)	0·91 (0·84-0·98)	1·99 (1·00-3·94)

[1] Multivariable logistic regression, adjusting for the other variables in the model –except self-reported mental illness. The model also adjusts for the observed significant interaction between Gravidity and Asset Index.

[2] The 95% confidence intervals were calculated using linearized standard errors

Frequencies are true counts, % and OR are weighted for design effects and non-response

Table 5: Adjusted[1] odds ratios and 95%CI for the association between gravidity and stillbirth stratified by the Asset index quintiles

**A· Missing Indicator Method**					
	**Asset Index Quintiles**				
	**1 (most deprived)**	**2**	**3**	**4**	**5 (least deprived)**
Gravidity					
1	1·00 (Ref)	1·00 (Ref)	1·00 (Ref)	1·00 (Ref)	1·00 (Ref)
2-4	1·44 (1·15-1·81)	1·47 (1·18-1·84)	1·83 (1·48 - 2·26)	2·55 (2·02 – 3·23)	3·04 (2·37 – 3·90)
More than 5	1·89 (1·43-2·49)	1·88 (1·43-2·48)	3·04 (2·34 - 3·94)	3·71 (2·75 - 5·02)	4·62 (3·27 - 6·54)
Missing	0·95 (0·74-1·22)	1·18 (0·92-1·50)	1·37 (1·09-1·72)	1·88 (1·44 – 2·45)	2·26 (1·67 – 3·05)
**B· Complete Case Analysis**					
	**Asset Index Quintiles**				
	**1**	**2**	**3**	**4**	**5**
Gravidity					
1	1·00 (Ref)	1·00 (Ref)	1·00 (Ref)	1·00 (Ref)	1·00 (Ref)
2-4	1·19 (0·92-1·54)	1·22 (0·94-1·60)	1·74 (1·35 - 2·24)	2·05 (1·56 – 2·67)	2·86 (2·11 – 3·88)
More than 5	1·59 (1·15-2·18)	1·48 (1·05-2·08)	2·70 (1·97 - 3·73)	3·02 (2·12 - 4·31)	5·02 (3·35 - 7·53)
**C· Multiple Imputations**					
	**Asset Index Quintiles**				
	**1**	**2**	**3**	**4**	**5**
Gravidity					
1	1·00 (Ref)	1·00 (Ref)	1·00 (Ref)	1·00 (Ref)	1·00 (Ref)
2-4	1·48 (0·90-2·48)	1·50 (0·90-2·55)	1·77 (1·08-2·92)	2·29 (1·39-3·84)	2·73 (2·20 – 3·40)
More than 5	1·90 (1·00-3·52)	1·98 (1·07-3·81)	2·69 (1·40-5·04)	3·31 (1·70-6·45)	4·41 (3·33 - 5·86)

[1]Multivariable logistic regression, adjusting for socioeconomic, health seeking behaviour, pre-existing medical conditions, and bio-demographic characteristics
Results are weighted for design effects and non-response

For peer review only - http://bmjopen.bmj.com/site/about/guidelines.xhtml

Association between specific complications during pregnancy and stillbirth

Reporting any pregnancy complication was associated with significantly higher odds of stillbirth after adjusting for the identified socioeconomic, behavioural, and bio-demographic risk factors (Table-4). The association between each of the reported complication and stillbirth are shown in Table-6. Women who reported to have anaemia during pregnancy had 35% higher odds of stillbirth compared with women who did not have anaemia. Eclampsia was associated with almost twice the odds of stillbirth and other hypertensive disorders were associated with about 22% higher odds. Women who had an ante-partum or intra-partum haemorrhage had significantly higher odds of stillbirth compared with women who did not have these complications. The adjusted odds of stillbirth were 58% higher in women with abnormal fetal position, more than three times higher for women who had an obstructed labour, and nearly three times higher for women who had a breech presentation compared with women who did not report these complications. Sensitivity analysis conducted using complete case analysis and multiple imputations did not change the results materially (Supplementary Table-S2).

For peer review only

Table 6: Association between stillbirth and complications during pregnancy–frequencies, unadjusted and adjusted Odds Ratios (95% CI)

Variables	Total births N=668,892 Frequency (%)	Stillbirths N= 6,777 Frequency (%)	Live births, N= 662,115 Frequency (%)	Unadjusted Odds ratios (95%CI)	Adjusted Odds ratios (95%CI)
**Anemia**					
No	500 785 (73.8)	4 611 (66.0)	496 174 (73.9)	1 (Ref)	1 (Ref)
Yes	168 107 (26.2)	2 166 (34.0)	165 941 (26.1)	1·45 (1·36-1·54)	1·35 (1·27-1·43)
**Eclampsia**					
No	628 681 (93.8)	6 043 (88.9)	622 638 (93.8)	1 (Ref)	1 (Ref)
Yes	40 211 (6.2)	734 (11.2)	39 477 (6.2)	1·90 (1·73 -2·10)	1·79 (1·62 -1·97)
**Other hypertensive disorders**					
No	639 689 (95.5)	6 412 (94.7)	633 277 (95.5)	1 (Ref)	1 (Ref)
Yes	29 203 (4.5)	365 (5.3)	28 838 (4.5)	1·20 (1·05-1·36)	1·22 (1·07-1·38)
**Intra-partum Hemorrhage**					
No	622 206 (93.3)	5 559 (82.6)	616 647 (93.4)	1 (Ref)	1 (Ref)
Yes	46 686 (6.7)	1 218 (17.4)	45 468 (6.6)	2·97 (2·75 -3·21)	2·75 (2·54 -2·97)
**Ante-partum Hemorrhage**					
No	650 631 (97.2)	6 488 (96.0)	644 143 (97.2)	1 (Ref)	1 (Ref)
Yes	18 261 (2.8)	289 (4.0)	17 972 (2.8)	1·44 (1·25-1·67)	1·29 (1·11-1·50)
**Obstructed Labor**					
No	602 184 (90.1)	5 037 (73.4)	597 147 (90.2)	1 (Ref)	1 (Ref)
Yes	66 708 (9.9)	1 740 (26.6)	64 968 (9.8)	3·35 (3·13-3·58)	3·45 (3·19-3·74)
**Breech presentation**					
No	645 162 (96.2)	6 092 (89.1)	639 070 (96.3)	1 (Ref)	1 (Ref)
Yes	23 730 (3.8)	685 (10.9)	23 045 (3.7)	3·18 (2·88-3·50)	2·80 (2·51-3·12)
**Abnormal Fetal position**					
No	640 015 (95.4)	6 269 (92.5)	633 746 (95.5)	1 (Ref)	1 (Ref)
Yes	28 877 (4.6)	508 (7.5)	28 369 (4.5)	1·72 (1·54-1·92)	1·58 (1·40-1·77)

[1]Each multivariable logistic regression model adjusts for socioeconomic, health seeking behaviour, and bio-demographic characteristics identified to be significantly associated with Stillbirth in the previous model.
[2] Although the sub-population was restricted to women who had complete data on pregnancy-specific complication, the other variables adjusted for in the model had missing data, therefore sensitivity analyses were conducted.
Frequencies are true counts, % and OR are weighted for design effects and non-response

For peer review only - http://bmjopen.bmj.com/site/about/guidelines.xhtml

DISCUSSION

The overall rate of stillbirth was found to be 10 per 1000 total births, but varied widely between the nine states ranging from 4.2 to 14.8 per 1000 total births. The marked variation at the state level may be explained by the different population characteristics and the varying distribution of risk factors across the nine states. The findings show the impact of inequality on stillbirth, as women in the most deprived groups are at highest risk. This was identified across a number of variables which capture different aspects of deprivation; for example, poorer women, those with little education, those living in rural areas, not currently working and belonging to 'schedule caste' groups are at increased risk of stillbirth compared to more affluent and advantaged women. Yet, among the multigravidas odds of stillbirth were substantially higher in the more affluent group, after adjusting for other factors. Chewing tobacco is highlighted as a significant risk factor for stillbirth, which is important in the Indian context where few women report tobacco smoking. Pregnancy complications, including anaemia, eclampsia, other hypertensive disorders, antepartum haemorrhage, intrapartum haemorrhage, abnormal fetal position, breech presentation, and obstructed labour significantly increased the odds of stillbirth.

The overall estimated stillbirth rate in the study population from the nine states in India was approximately half that of the World Health Organisation (WHO) estimated rate of 22 per 1000 total births. Possible explanations include under-reporting of stillbirths or classifying stillbirths as neonatal deaths due to associated stigma. Nevertheless, we found a significant disparity in the risk of stillbirth by socioeconomic status which is consistent with previous research. A systematic review of studies from developing countries showed that low socioeconomic status was significantly associated with stillbirth with a population attributable fraction ranging between 2% and 75%.[9] A study from 13 European countries reported that lower maternal education and maternal unemployment were associated with 1.9 and 1.6 times higher odds of stillbirth, respectively.[9] Maternal education and employment may act through promoting high self-esteem and empowering women to make decisions about healthcare utilisation.[10]

The increased odds of stillbirth in women belonging to religious minority groups in India could be due to cultural constraints reported in other studies or inequalities that minority groups are subjected to which influences their healthcare seeking behaviours, even in countries that have universal access to medical care.[11] In India, Schedule tribe and Schedule caste social groups have been historically disadvantaged, belonging to a Schedule caste group was associated with higher odds of stillbirth which is consistent with previous literature of disadvantaged groups having higher risk.[12] However, women

For peer review only - http://bmjopen.bmj.com/site/about/guidelines.xhtml

belonging to the Schedule tribe group had lower odds of stillbirth. While, it might be possible that there is differential underreporting of stillbirth, it is also possible that individuals belonging to Schedule tribe groups have different health seeking behaviours leading to a lower risk.[13 14]

Self-reported tobacco smoking or chewing has been shown to be associated with increased risk of stillbirth in other studies.[15 16] In the study population, chewing tobacco was 15-times more common compared with smoking tobacco and was associated with 14% higher odds of stillbirth. In contrast to other studies, smoking was not found to be significantly associated with stillbirth in our study population after adjusting for other risk factors. This could be because smoking at the time of the survey may not reflect smoking during pregnancy. While smoking during pregnancy is discouraged, healthcare messages about chewing tobacco during pregnancy are less clear.

Our findings related to timing and number of ANC visits conforms to the results of other studies. A study by Chopra et al estimated that 24% of stillbirth and perinatal deaths in South Africa could be prevented every year through improved use of ANC services.[17] However another study from South Africa found that timing of booking visit may not, in isolation, be an important determinant of stillbirth.[18] Assisted delivery and caesarean section were found to be associated with higher risk of stillbirth, but this association was grossly attenuated and no longer statistically significant for caesarean section after adjusting for obstructed labour, suggesting the presence of a reverse causation. Similar to other studies, our study showed a U-shaped association between maternal age and stillbirth[19-21] and male sex of the fetus to be associated with higher odds of stillbirth.[1 22] The higher odds of stillbirth observed with multiple pregnancies compared with singleton pregnancies can be explained by propensity of women with multiple pregnancies to have more complications.[23]

A number of studies have shown that anaemia during pregnancy is associated with increased risk of stillbirth, with 3.7-16 times higher odds of stillbirth associated with anaemia among pregnant women.[24 25] A meta-analysis of risk factors in high income countries reported higher odds of stillbirth for women with pregnancy-induced hypertension, pre-eclampsia and eclampsia.[26] Antepartum and intrapartum haemorrhage can be due to a myriad of obstetric complications, a meta-analysis reported that there was a four-fold increased risk of stillbirth associated with bleeding.[27] Breech presentation, abnormal fetal position, and obstructed labour operate through similar mechanisms, the fetus is trapped in the birth canal and is subjected to hypoxia leading to stillbirth.[28] However, a stillbirth could be prevented if these complications are detected timely and managed appropriately.

For peer review only - http://bmjopen.bmj.com/site/about/guidelines.xhtml

Previous studies suggest that women with pre-existing mental health problems have 70% higher risk of stillbirth.[29]

The Annual Health Survey is the largest health survey in the world. The large sample provided adequate number of events (stillbirths) and allowed us to conduct an adequately powered, robust examination of a wide range of risk factors. The survey is a representative sample from nine states in India with high rates of stillbirth and the sampling strategy minimised the possibility of selection bias. Findings are therefore generalisable to India. To our knowledge, no other large dataset is available to investigate stillbirth in India, the country with the highest number of stillbirths.

However, there are some limitations of this study which should be considered when interpreting the findings. Important to consider is that stillbirths may be under-reported in the study from which our data were drawn. Retrospective data collection could also have led to potential recall bias, and self-reported complications during pregnancy were not verified with hospital records. It was not possible to differentiate between pregnancy-induced hypertension and pre-existing essential hypertension. To minimise the effect of recall bias, dummy variables (e.g. anaemia) were generated wherever feasible and highly subjective symptoms (e.g. prolonged labour) were not investigated. Data were not available for a number of potential risk factors for stillbirth such as timing of stillbirth, thus we were not able to analyse the risk factors separately for antepartum and intrapartum stillbirths. An inherent limitation of routine survey data is missing information; however, the results of the analyses using three different methods to account for missing data were not substantially different.

Conclusion

Our study showed that despite several efforts being made to improve pregnancy care in India, socioeconomic disparities in stillbirth still exits and maternal and fetal complications were found to be important preventable risk factors. Another important finding was the risk associated with chewing tobacco which demonstrates an urgent need for strong public health messages to stop chewing tobacco during pregnancy in addition to the messages to stop smoking. Improving uptake of antenatal care, and timely identification and effective management of maternal and fetal complications could reduce preventable stillbirths. Eradicating poverty and promotion of female education are part of the global developmental agenda espoused by the Sustainable Development Goals, for which India is a signatory. This study showed that progress towards these goals could accelerate progress in preventing stillbirths.

For peer review only - http://bmjopen.bmj.com/site/about/guidelines.xhtml

Studies are needed to identify risk factors separately for antepartum and intrapartum stillbirths in India. The association between the maternal and fetal complications and stillbirth suggests that although at present, pregnant women in India are incentivised to seek antenatal care and give birth in health institutions, the quality of pregnancy care need further investigation. Currently, neither are all stillbirths recorded nor are local reviews of stillbirths conducted in the country. Targeting at-risk population groups, recording all stillbirths and conducting local reviews would be important to reduce the high burden of stillbirths in India.

REFERENCE LIST

1. Lawn JE, Blencowe H, Waiswa P, et al. Stillbirths: rates, risk factors, and acceleration towards 2030. *The Lancet* 2016;387(10018):587-603. doi: http://dx.doi.org/10.1016/S0140-6736(15)00837-5
2. Frøen JF, Friberg IK, Lawn JE, et al. Stillbirths: progress and unfinished business. *The Lancet*;387(10018):574-86. doi: 10.1016/S0140-6736(15)00818-1
3. Blencowe H, Cousens S, Jassir FB, et al. National, regional, and worldwide estimates of stillbirth rates in 2015, with trends from 2000: a systematic analysis. *The Lancet Global Health* 2016;4(2):e98-e108. doi: http://dx.doi.org/10.1016/S2214-109X(15)00275-2
4. Sharma D. India newborn action plan. *International Journal of Medical Science Research and Practice* 2015;2(2):58.
5. Di Mario S, Say L, Lincetto O. Risk Factors for Stillbirth in Developing Countries: A Systematic Review of the Literature. *Sexually Transmitted Diseases* 2007;34(7):S11-S21. doi: 10.1097/01.olq.0000258130.07476.e3
6. Ministry of Health and Family Welfare (MoHFW) GoI. National Health Portal: Janani Suraksha Yojana (JSY) Ne Delhi: National Institute of Health and Family Welfare (NIHFW), MoHFW; [cited 2017 18 December]. accessed 18 December 2017.
7. Office of Registrar General & Census Commissioner MoHA, Government of India. Annual Health Survey Report: A report on core and vital health indicators part I. 2014
8. Pedersen AB, Mikkelsen EM, Cronin-Fenton D, et al. Missing data and multiple imputation in clinical epidemiological research. *Clinical Epidemiology* 2017;9:157.
9. McClure EM, Goldenberg RL. Stillbirth in Developing Countries: A review of causes, risk factors and prevention strategies. *The journal of maternal-fetal & neonatal medicine : the official journal of the European Association of Perinatal Medicine, the Federation of Asia and Oceania Perinatal Societies, the International Society of Perinatal Obstetricians* 2009;22(3):183-90. doi: 10.1080/14767050802559129
10. Pincus T, Callahan LF. Associations of low formal education level and poor health status: behavioral, in addition to demographic and medical, explanations? *Journal of clinical epidemiology* 1994;47(4):355-61.
11. Penn N, Oteng-Ntim E, Oakley LL, et al. Ethnic variation in stillbirth risk and the role of maternal obesity: analysis of routine data from a London maternity unit. *BMC pregnancy and childbirth* 2014;14(1):404.
12. Smith GCS, Fretts RC. Stillbirth. *The Lancet* 2007;370(9600):1715-25. doi: http://dx.doi.org/10.1016/S0140-6736(07)61723-1
13. Moffatt M. An untouchable community in South India: Structure and consensus: Princeton University Press 2015.

For peer review only - http://bmjopen.bmj.com/site/about/guidelines.xhtml

14. Navaneetham K, Dharmalingam A. Utilization of maternal health care services in Southern India. *Social science & medicine (1982)* 2002;55(10):1849-69. [published Online First: 2002/10/18]
15. Gupta PC, Subramoney S. Smokeless tobacco use and risk of stillbirth: a cohort study in Mumbai, India. *Epidemiology (Cambridge, Mass)* 2006;17(1):47-51. [published Online First: 2005/12/17]
16. Marufu TC, Ahankari A, Coleman T, et al. Maternal smoking and the risk of still birth: systematic review and meta-analysis. *BMC Public Health* 2015;15:239. doi: 10.1186/s12889-015-1552-5
17. Neogi SB, Negandhi P, Chopra S, et al. Risk Factors for Stillbirth: Findings from a Population-Based Case–Control Study, Haryana, India. *Paediatric and perinatal epidemiology* 2016;30(1):56-66.
18. Beauclair R, Petro G, Myer L. The association between timing of initiation of antenatal care and stillbirths: a retrospective cohort study of pregnant women in Cape Town, South Africa. *BMC Pregnancy and Childbirth* 2014;14(1):204. doi: 10.1186/1471-2393-14-204
19. Andersen A-MN, Wohlfahrt J, Christens P, et al. Maternal age and fetal loss: population based register linkage study. *BMJ* 2000;320(7251):1708-12. doi: 10.1136/bmj.320.7251.1708
20. Althabe F, Moore JL, Gibbons L, et al. Adverse maternal and perinatal outcomes in adolescent pregnancies: The Global Network's Maternal Newborn Health Registry study. *Reproductive Health* 2015;12(Suppl 2):S8-S8. doi: 10.1186/1742-4755-12-S2-S8
21. Khandait DW, Ambadekar NN, Zodpey SP, et al. Maternal age as a risk factor for stillbirth. *Indian journal of public health* 2000;44(1):28-30. [published Online First: 2001/07/07]
22. Mondal D, Galloway TS, Bailey TC, et al. Elevated risk of stillbirth in males: systematic review and meta-analysis of more than 30 million births. *BMC Medicine* 2014;12:220. doi: 10.1186/s12916-014-0220-4
23. Kurdi AM, Mesleh RA, Al-Hakeem MM, et al. Multiple pregnancy and preterm labor. *Saudi medical journal* 2004;25(5):632-7. [published Online First: 2004/05/13]
24. Lone FW, Qureshi RN, Emanuel F. Maternal anaemia and its impact on perinatal outcome. *Tropical Medicine & International Health* 2004;9(4):486-90.
25. Engelgau MM, Sampson UK, Rabadan-Diehl C, et al. Tackling NCD in LMIC: Achievements and Lessons Learned From the NHLBI–UnitedHealth Global Health Centers of Excellence Program. *Global Heart* 2016;11(1):5-15. doi: http://dx.doi.org/10.1016/j.gheart.2015.12.016
26. Flenady V, Koopmans L, Middleton P, et al. Major risk factors for stillbirth in high-income countries: a systematic review and meta-analysis. *Lancet* 2011;377 doi: 10.1016/s0140-6736(10)62233-7
27. Ananth CV, Savitz DA. Vaginal bleeding and adverse reproductive outcomes: a meta-analysis. *Paediatric and Perinatal Epidemiology* 1994;8(1):62-78. doi: 10.1111/j.1365-3016.1994.tb00436.x
28. Lawn J, Shibuya K, Stein C. No cry at birth: global estimates of intrapartum stillbirths and intrapartum-related neonatal deaths. *Bulletin of the World Health Organization* 2005;83(6):409-17.
29. Gardosi J, Madurasinghe V, Williams M, et al. Maternal and fetal risk factors for stillbirth: population based study. *Bmj* 2013;346:f108.

AUTHOR STATEMENT

Disclosure of interest

All authors declare: no support from any organisation for the submitted work; no financial relationships with any organisations that might have an interest in the submitted work in the previous three years; no other relationships or activities that could appear to have influenced the submitted work.

For peer review only - http://bmjopen.bmj.com/site/about/guidelines.xhtml

Contribution to authorship

NA reviewed the literature, conducted the analysis and wrote the first draft of the paper; CC helped with the data analysis, and edited the paper; SSC helped in acquiring and interpreting the data, and edited the paper; AR edited the paper; UCS edited the paper; MK contributed to the interpretation and discussion of the results, and edited the paper; MN developed the concept for the study, supervised the data analysis, interpretation and discussion of the results, and edited the paper.

Details of ethics approval

Ethics approval was not required since this was a secondary analysis of anonymous survey data.

Funding

The study was funded by a Medical Research Council Career Development Award to Manisha Nair (Grant Ref: MR/P022030/1). The funder had no role in the study design, data analysis, data interpretation, or writing of the manuscript. All authors, had full access to all of the data in the study and can take responsibility for the integrity of the data and the accuracy of the data analysis.

Data sharing statement

The anonymised data is freely available through the Indian Government's Data Sharing Portal.

Figure legends

Figure 1: Flowchart showing the derivation of the final study population

Figure 2: Hypothesized relationship between the risk factors and stillbirth

Figure 3: Association between maternal age in 5 year groups and stillbirth

Figure-4: Rate of stillbirth overall and by nine states in India using the Annual Health Survey data (2012-13)

For peer review only - http://bmjopen.bmj.com/site/about/guidelines.xhtml

Figure 1: Flowchart showing the derivation of the final study population

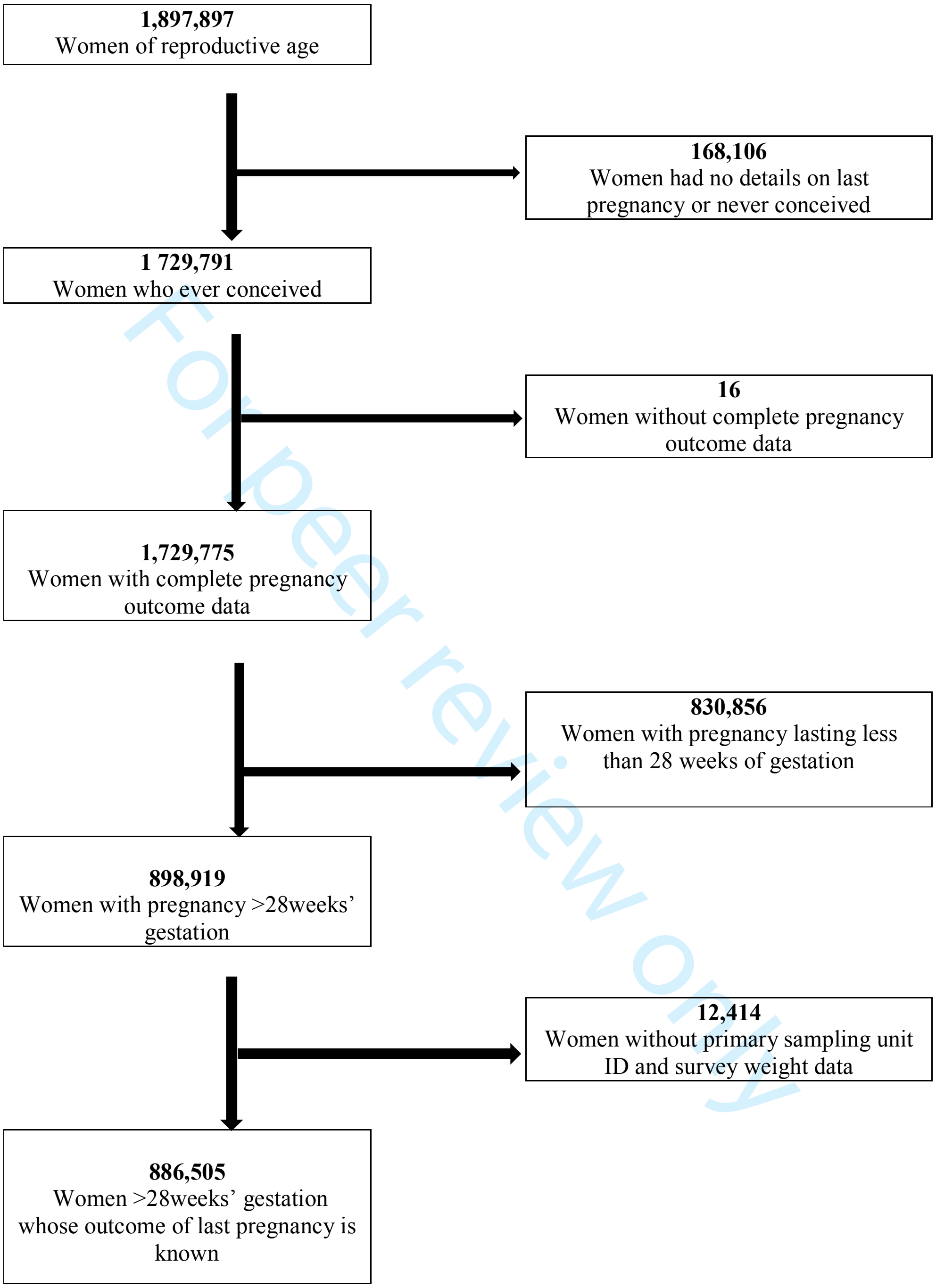

For peer review only

Figure 2: Hypothesized relationship between the risk factors and stillbirth

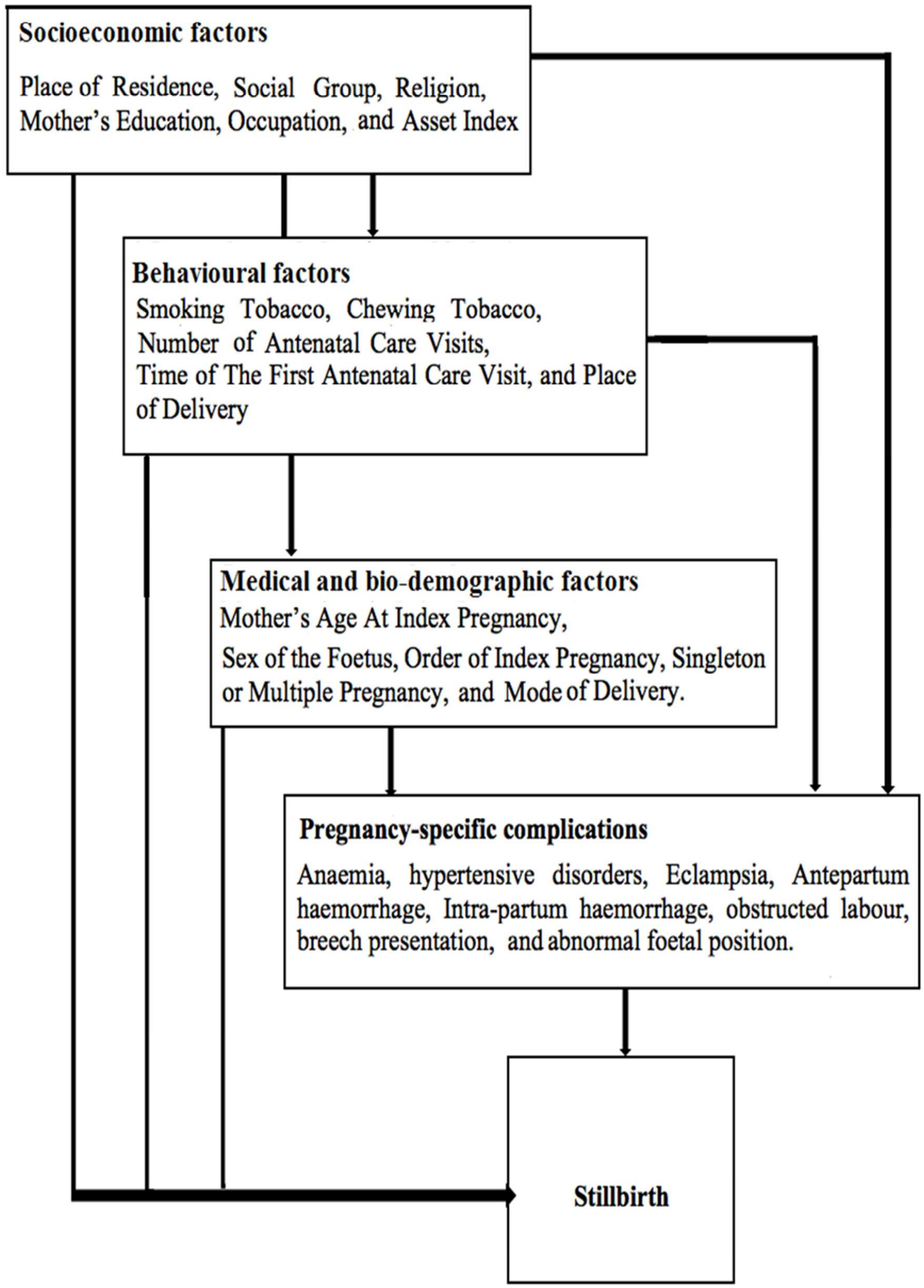

Figure 3: Association between maternal age in 5 year groups and stillbirth

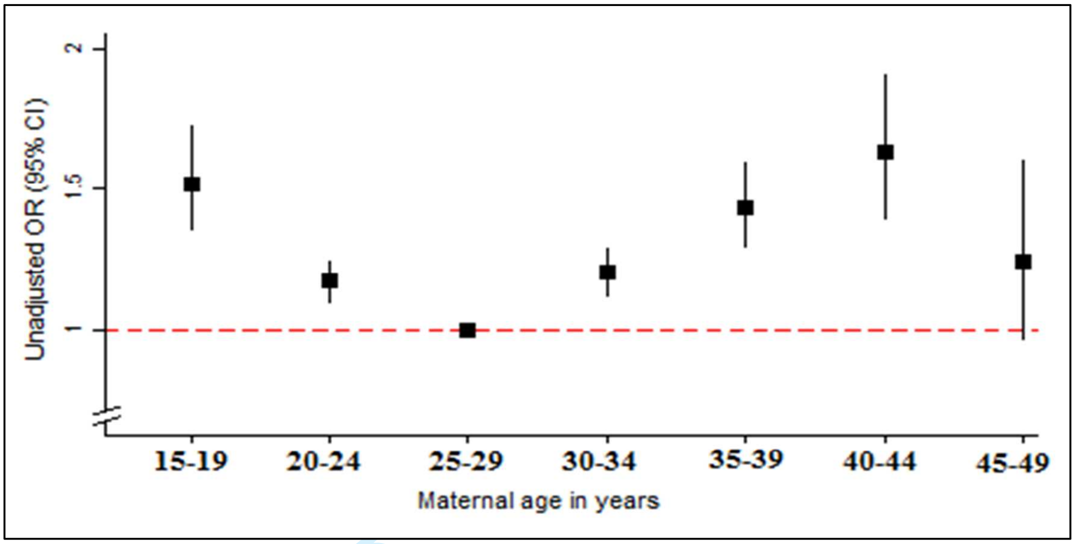

Data source: Annual Health Survey 2012-13, India

For peer review only

Figure-4: Rate of stillbirth overall and by nine states in India using the Annual Health Survey data (2012-13)

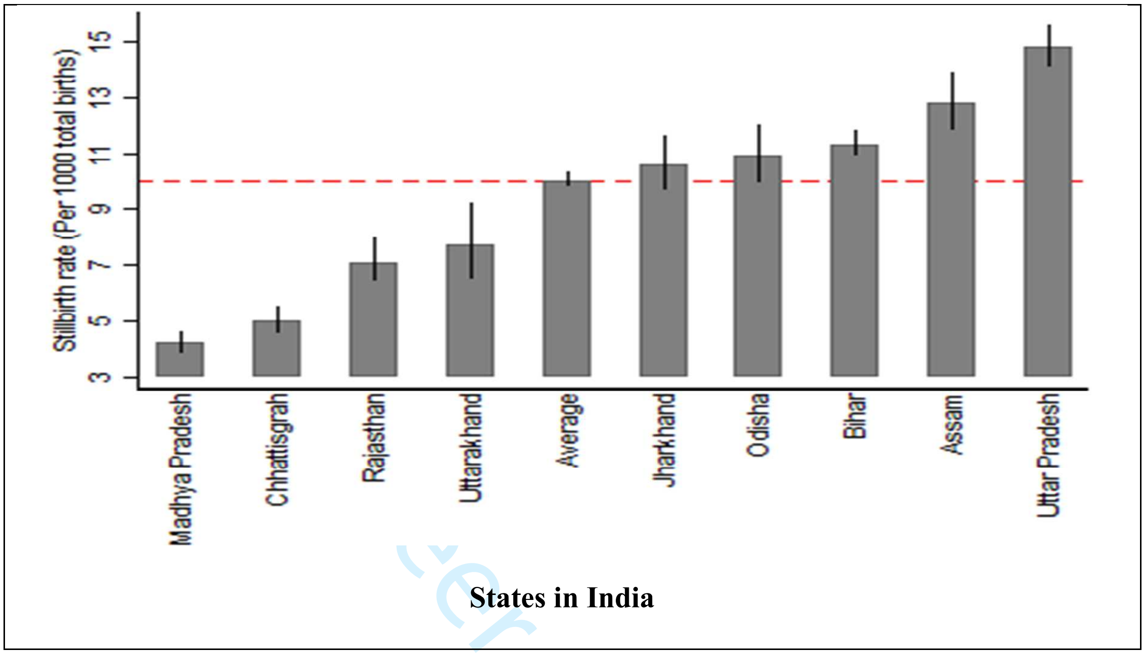

Table-S1: Unadjusted and adjusted[1] odds ratios (OR) and 95%CI[2] for stillbirth by socioeconomic, health seeking behaviour, pre-existing medical conditions, and bio-demographic factors

Variables	Unadjusted Odds Ratios (95%CI)	Main model	Sensitivity analysis	
		Missing indicator Adjusted Odds Ratios (95%CI)	Complete case analysis Adjusted Odds Ratios (95%CI)	Multiple imputation Adjusted Odds Ratios (95%CI)
**Maternal Age (years)**				
15-19	1·52 (1·35-1·72)	1·76 (1·55-2·00)	1·58 (1·30-1·93)	1·79 (1·55 - 2·07)
20-24	1·17 (1·09-1·24)	1·29 (1·21-1·37)	1·21 (1·10-1·32)	1·29 (1·20 - 1·39)
25-29	1·00 (Ref)	1·00 (Ref)	1·00 (Ref)	1·00 (Ref)
30-34	1·20 (1·11-1·29)	1·05 (0·97-1·14)	1·13 (1·01-1·26)	1·07 (0·98 - 1·18)
35-39	1·43 (1·29-1·59)	1·16 (1·04-1·29)	1·18 (1·00-1·38)	1·18 (1·04 - 1·34)
40-45	1·63 (1·39-1·91)	1·29 (1·09-1·51)	1·42 (1·12-1·79)	1·37 (1·14 - 1·65)
45-49	1·24 (0·96-1·60)	1·04 (0·80-1·35)	1·05 (0·72-1·55)	1·05 (0·82 - 1·49)
**Place of Residence**				
Urban	1·00 (Ref)	1·00 (Ref)	1·00 (Ref)	1·00 (Ref)
Rural	1·35 (1·24-1·47)	1·27 (1·16 - 1·39)	1·31 (1·15-1·49)	1·25 (1·13 - 1·38)
**Religion**				
Hindu	1·00 (Ref)	1·00 (Ref)	1·00 (Ref)	1·00 (Ref)
Muslim	1·48 (1·39-1·58)	1·33 (1·25-1·43)	1·35 (1·22-1·49)	1·40 (1·29 - 1·51)
Christian	1·49 (1·25-1·78)	1·42 (1·19-1·70)	1·53 (1·25-1·86)	1·44 (1·20 - 1·73)
Others	0·87 (0·69-1·11)	1·06 (0·84-1·35)	0·85 (0·60-1·19)	1·09 (0·85 - 1·40)
**Social Group**				
Other	1·00 (Ref)	1·00 (Ref)	1·00 (Ref)	1·00 (Ref)
Schedule Caste	1·09 (1·02-1·16)	1·11 (1·04-1·19)	1·16 (1·05-1·28)	1·14 (1·05 - 1·23)
Schedule Tribe	0·68 (0·62-0·74)	0·74 (0·68-0·81)	0·67 (0·59-0·76)	0·71 (0·64 - 0·79)
**Maternal Education**				
Tertiary and above	1·00 (Ref)	1·00 (Ref)	1·00 (Ref)	1·00 (Ref)
Secondary	1·31 (1·08-1·60)	1·10 (0·90-1·34)	1·11 (0·84-1·47)	1·04 (0·84 - 1·30)
Primary and below	1·77 (1·48-2·12)	1·34 (1·10-1·62)	1·25 (0·95-1·64)	1·24 (1·00 - 1·53)
Illiterate	2·06 (1·72-2·47)	1·43 (1·17-1·74)	1·34 (1·02-1·77)	1·31 (1·06 - 1·62)
**Employment**				
In paid employment	1·00 (Ref)	1·00 (Ref)	1·00 (Ref)	1·00 (Ref)
Not in-paid employment	1·29 (1·20-1·38)	1·15 (1·07-1·24)	1·22 (1·10-1·36)	1·19 (1·09-1·29)
**Asset Index, Quintiles**				
5 “Highest”	1·00 (Ref)	1·00 (Ref)	1·00 (Ref)	1·00 (Ref)
4	1·26 (1·14-1·38)	1·34 (1·01-1·79)	1·58 (1·10-2·28)	1·32 (0·99-1·75)
3	1·41 (1·29-1·55)	1·91 (1·45-2·52)	2·13 (1·49-3·03)	1·81 (1·39-2·34)
2	1·46 (1·33-1·60)	2·45 (1·85-3·24)	2·64 (1·83-3·78)	2·23 (1·69-2·92)
1 “Lowest”	1·39 (1·26-1·52)	2·42 (1·82-3·21)	3·14 (2·20-4·47)	2·24 (1·71-2·94)
Missing	1·41 (1·27-1·56)	1·21 (0·91-1·62)	-	-

**Chewing Tobacco**[3]				
No	1·00 (Ref)	1·00 (Ref)	1·00 (Ref)	1·00 (Ref)
Yes	1·10 (1·01-1·19)	1·11 (1·02-1·21)	1·07 (0·97-1·19)	1·12 (1·00-1·25)
Missing	1·16 (1·09-1·24)	1·44 (1·29-1·59)	-	-
**Number of antenatal care visit**				
4 or more	1·00 (Ref)	1·00 (Ref)	1·00 (Ref)	1·00 (Ref)
Less than 4	1·19 (1·12-1·26)	1·08 (1·01-1·15)	1·11 (1·03-1·21)	1·06 (0·99-1·14)
Missing	1·53 (1·41-1·66)	1·36 (1·25-1·48)	-	-
**Timing of the first antenatal**				
**care visit**	1·00 (Ref)	-	-	-
Three or less	1·12 (1·06-1·19)	-	-	-
More than three	1·41 (1·31-1·52)	-	-	-
Missing				
**Place of delivery**				
Medical facility	1·00 (Ref)	-	-	-
Home	0·94 (0·89-0·99)	-	-	-
**Number of pregnancies**				
1	1·00 (Ref)	1·00 (Ref)	1·00 (Ref)	1·00 (Ref)
2-4	1·77 (1·63-1·93)	3·06 (2·42-3·86)	2·95 (2·20-3·94)	2·72 (2·18-3·38)
5 or more	2·60 (2·36-2·87)	4·98 (3·66-6·74)	5·35 (3·73-7·67)	4·30 (3·24-5·70)
Missing	1·21 (1·10-1·32)	1·40 (0·68-2·90)	-	-
**Sex of the baby**				
Female	1·00 (Ref)	1·00 (Ref)	1·00 (Ref)	1·00 (Ref)
Male	1·25 (1·19-1·32)	1·26 (1·20 - 1·33)	1·23 (1·14-1·32)	1·25 (1·18 - 1·32)
**Multiple gestations (index**				
**pregnancy)**	1·00 (Ref)	1·00 (Ref)	1·00 (Ref)	1·00 (Ref)
Singleton	2·03 (1·67-2·45)	1·77 (1·47 - 2·15)	1·90 (1·46-2·49)	1·84 (1·49 - 2·28)
Twin pregnancy				
**Mode of delivery**				
Spontaneous vaginal	1·00 (Ref)	1·00 (Ref)	1·00 (Ref)	1·00 (Ref)
Assisted vaginal	3·13 (2·76-3·55)	3·45 (3·02-3·93)	3·88 (3·25-4·63)	3·63 (3·16-4·18)
Caesarean section	1·53 (1·40-1·66)	1·73 (1·58-1·89)	1·75 (1·56-1·98)	1·58 (1·43-1·74)
**Any pregnancy complications**				
No	1·00 (Ref)	1·00 (Ref)	1·00 (Ref)	1·00 (Ref)
Yes	1·47 (1·38-1·56)	1·42 (1·33-1·51)	1·44 (1·33-1·56)	1·40 (1·31-1·49)
Missing	0·91 (0·84-0·98)	1·99 (1·00-3·94)	-	-

[1] Multivariable logistic regression, adjusting for the other variables in the model –except self-reported mental illness. The model also adjusts for the observed significant interaction between Gravidity and Asset Index.
[2] The 95% confidence intervals were calculated using linearized standard errors
[3] These variables reflect practice at the time of the survey
Results are weighted for design effects and non-response

Table-S2: Sensitivity analysis - Unadjusted and adjusted[1] odds ratios (95% CI[2]) for stillbirth by pregnancy complications

Variable	Unadjusted Odds ratios (95%CI)	Main Model	Sensitivity analyses	
		Missing indicator method Adjusted Odds ratios (95%CI)	Complete case analysis[2] Adjusted Odds ratios (95%CI)	Multiple imputations[2] Adjusted Odds ratios (95%CI)
**Anemia**				
No	1 (Ref)	1 (Ref)	1 (Ref)	1 (Ref)
Yes	1·45 (1·36-1·54)	1·35 (1·27-1·43)	1·41 (1·30-1·52)	1·34 (1·26-1·42)
**Eclampsia**				
No	1 (Ref)	1 (Ref)	1 (Ref)	1 (Ref)
Yes	1·90 (1·73 -2·10)	1·79 (1·62 -1·97)	1·94 (1·72 -2·19)	1·79 (1·62 -1·98)
**Other hypertensive disorders**				
No	1 (Ref)	1 (Ref)	1 (Ref)	1 (Ref)
Yes	1·20 (1·05-1·36)	1·22 (1·07-1·38)	1·25 (1·07-1·46)	1·21 (1·06-1·38)
**Intra-partum Hemorrhage**				
No	1 (Ref)	1 (Ref)	1 (Ref)	1 (Ref)
Yes	2·97 (2·75 -3·21)	2·75 (2·54 -2·97)	2·73 (2·47 -3·02)	2·77 (2·56 -3·00)
**Ante-partum Hemorrhage**				
No	1 (Ref)	1 (Ref)	1 (Ref)	1 (Ref)
Yes	1·44 (1·25-1·67)	1·29 (1·11-1·50)	1·51 (1·39-1·64)	1·32 (1·14-1·53)
**Obstructed Labor**				
No	1 (Ref)	1 (Ref)	1 (Ref)	1 (Ref)
Yes	3·35 (3·13-3·58)	3·45 (3·19-3·74)	3·57 (3·24-3·94)	3·42 (3·16-3·70)
**Breech presentation**				
No	1 (Ref)	1 (Ref)	1 (Ref)	1 (Ref)
Yes	3·18 (2·88-3·50)	2·80 (2·51-3·12)	3·04 (2·65-3·47)	2·85 (2·56-3·18)
**Abnormal Fetal position**				
No	1 (Ref)	1 (Ref)	1 (Ref)	1 (Ref)
Yes	1·72 (1·54-1·92)	1·58 (1·40-1·77)	1·66 (1·43-1·91)	1·60 (1·43-1·80)

[1]Each multivariable logistic regression model adjusts for socioeconomic, health seeking behaviour, and bio-demographic characteristics identified to be significantly associated with Stillbirth in the previous model.
[2] Although the sub-population was restricted to women who had complete data on pregnancy-specific complication, the other variables adjusted for in the model had missing data, therefore sensitivity analyses were conducted.
Results are weighted for design effects and non-response

STROBE Statement—Checklist of items that should be included in reports of ***cross-sectional studies***

	Item No	Recommendation	Page No
**Title and abstract**	1	(*a*) Indicate the study's design with a commonly used term in the title or the abstract	1,2
		(*b*) Provide in the abstract an informative and balanced summary of what was done and what was found	2
**Introduction**			
Background/rationale	2	Explain the scientific background and rationale for the investigation being reported	4
Objectives	3	State specific objectives, including any prespecified hypotheses	4
**Methods**			
Study design	4	Present key elements of study design early in the paper	4
Setting	5	Describe the setting, locations, and relevant dates, including periods of recruitment, exposure, follow-up, and data collection	4,5
Participants	6	(*a*) Give the eligibility criteria, and the sources and methods of selection of participants	4, Figure-1
Variables	7	Clearly define all outcomes, exposures, predictors, potential confounders, and effect modifiers. Give diagnostic criteria, if applicable	4,5,6
Data sources/ measurement	8*	For each variable of interest, give sources of data and details of methods of assessment (measurement). Describe comparability of assessment methods if there is more than one group	5,6
Bias	9	Describe any efforts to address potential sources of bias	6,7
Study size	10	Explain how the study size was arrived at	7
Quantitative variables	11	Explain how quantitative variables were handled in the analyses. If applicable, describe which groupings were chosen and why	5,6,7
Statistical methods	12	(*a*) Describe all statistical methods, including those used to control for confounding	6,7
		(*b*) Describe any methods used to examine subgroups and interactions	6,7
		(*c*) Explain how missing data were addressed	7
		(*d*) If applicable, describe analytical methods taking account of sampling strategy	7
		(*e*) Describe any sensitivity analyses	7
**Results**			
Participants	13*	(a) Report numbers of individuals at each stage of study—eg numbers potentially eligible, examined for eligibility, confirmed eligible, included in the study, completing follow-up, and analysed	4 and Figure-1
		(b) Give reasons for non-participation at each stage	Figure-1
		(c) Consider use of a flow diagram	Figure-1
Descriptive data	14*	(a) Give characteristics of study participants (eg demographic, clinical, social) and information on exposures and potential confounders	7,8,9
		(b) Indicate number of participants with missing data for each	10,11,12

For peer review only

		variable of interest	
Outcome data	15*	Report numbers of outcome events or summary measures	7,8
Main results	16	(*a*) Give unadjusted estimates and, if applicable, confounder-adjusted estimates and their precision (eg, 95% confidence interval). Make clear which confounders were adjusted for and why they were included	8, 9,10,11,12, 13,14, 15
		(*b*) Report category boundaries when continuous variables were categorized	9
		(*c*) If relevant, consider translating estimates of relative risk into absolute risk for a meaningful time period	Not applicable
Other analyses	17	Report other analyses done—eg analyses of subgroups and interactions, and sensitivity analyses	Table-S1 and S2
**Discussion**			
Key results	18	Summarise key results with reference to study objectives	16
Limitations	19	Discuss limitations of the study, taking into account sources of potential bias or imprecision. Discuss both direction and magnitude of any potential bias	18
Interpretation	20	Give a cautious overall interpretation of results considering objectives, limitations, multiplicity of analyses, results from similar studies, and other relevant evidence	16,17,18
Generalisability	21	Discuss the generalisability (external validity) of the study results	17,18
**Other information**			
Funding	22	Give the source of funding and the role of the funders for the present study and, if applicable, for the original study on which the present article is based	21

*Give information separately for exposed and unexposed groups.

Note: An Explanation and Elaboration article discusses each checklist item and gives methodological background and published examples of transparent reporting. The STROBE checklist is best used in conjunction with this article (freely available on the Web sites of PLoS Medicine at http://www.plosmedicine.org/, Annals of Internal Medicine at http://www.annals.org/, and Epidemiology at http://www.epidem.com/). Information on the STROBE Initiative is available at www.strobe-statement.org.

For peer review only

BMJ Open

Stillbirth among women in nine states in India: Rate and risk factors in study of 886,505 women from the Annual Health Survey

Journal:	*BMJ Open*
Manuscript ID	bmjopen-2018-022583.R1
Article Type:	Research
Date Submitted by the Author:	16-May-2018
Complete List of Authors:	Altijani , Noon; University of Oxford, Nuffield Department of Population Health<br>Carson, Claire; University of Oxford, National Perinatal Epidemiology Unit (NPEU); National Perinatal Epidemiology Unit (NPEU),<br>Choudhury , Saswati; FAA Medical College and Hospital, Department of Obstetrics and Gynaecology<br>Rani, Anjali; Banaras Hindu University Institute of Medical Sciences, Department of Obstetrics and Gynaecology<br>Sarma, Umesh; Srimanta Sankaradeva University of Health Sciences<br>Knight, Marian; National Perinatal Epidemiology Unit<br>Nair, Manisha; University of Oxford, NPEU, Nuffield Department of Population Health
**Primary Subject Heading**:	Epidemiology
Secondary Subject Heading:	Global health, Public health
Keywords:	Stillbirth, Risk factors, Pregnancy complications, India

SCHOLARONE™
Manuscripts

TITLE PAGE

Stillbirth among women in nine states in India: Rate and risk factors in study of 886,505 women from the Annual Health Survey

Authors

1. Noon Altijani (MSc)
   Nuffield Department of Population Health, University of Oxford
   Richard Doll Building, Old Road Campus, Headington, Oxford, OX3 7LF, UK
   Email: noon.altijani@gtc.ox.ac.uk

2. Claire Carson (PhD)
   National Perinatal Epidemiology Unit, Nuffield Department of Population Health, University of Oxford
   Richard Doll Building, Old Road Campus, Headington, Oxford, OX3 7LF, UK
   Email: claire.carson@npeu.ox.ac.uk

3. Saswati Sanyal Choudhury (MD)
   Department of Obstetrics and Gynaecology, FAA Medical College and Hospital, Barpeta, Barpeta-Hospital-Jania Road, Joti Gaon, Barpeta, Assam 781301, India
   Email: saswatischoudhury@gmail.com

4. Anjali Rani (MD)
   Department of Obstetrics and Gynaecology, Institute of Medical Sciences, Banaras Hindu University, Ajagara, Banaras Hindu University Campus, Varanasi, Uttar Pradesh 221005, India
   Email: anjalirani.imsbhu@gmail.com

5. Umesh C Sarma (MD)
   Srimanta Sankaradeva University of Health Sciences, Assam
   Narkashur Hilltop, Christian Basti Bhangagarh, Guwahati, Assam 781032, India
   Email: ssuhs_assam@yahoo.in

6. Marian Knight (DPhil)
   National Perinatal Epidemiology Unit, Nuffield Department of Population Health, University of Oxford
   Richard Doll Building, Old Road Campus, Headington, Oxford, OX3 7LF, UK.
   Email: marian.knight@npeu.ox.ac.uk

7. Manisha Nair (DPhil)
   National Perinatal Epidemiology Unit, Nuffield Department of Population Health, University of Oxford
   Richard Doll Building, Old Road Campus, Headington, Oxford, OX3 7LF, UK
   Email: manisha.nair@npeu.ox.ac.uk

Corresponding author

Manisha Nair
Senior Epidemiologist and Research Fellow,
National Perinatal Epidemiology Unit, NDPH, University of Oxford
Richard Doll Building, Old Road Campus, Headington, Oxford, OX3 7LF, UK
Email: manisha.nair@npeu.ox.ac.uk; Phone: +44-(0)1865-617820

For peer review only

For peer review only - http://bmjopen.bmj.com/site/about/guidelines.xhtml

ABSTRACT

Objectives: To assess the rate of stillbirth and associated risk factors across nine states in India.

Design: Secondary analysis of cross-sectional data from the Indian Annual Health Survey (2010-2013).

Setting: Nine states in India: Madhya Pradesh, Chattisgarh, Rajasthan, Uttarakhand, Jharkhand, Odisha, Bihar, Assam, Uttar Pradesh

Participants: 886,505 women, aged 15–49 years

Main outcome measures: Stillbirth rate with 95% Confidence Interval (CI). Adjusted odds ratio to examine the associations between stillbirth and (i) socioeconomic, behavioural, and bio-demographic risk factors (ii) complications in pregnancy (anaemia, eclampsia, other hypertensive disorders, antepartum and intrapartum haemorrhage, obstructed labour, breech presentation, abnormal fetal position).

Results: The overall rate of stillbirth was 10 per 1000 total births (95%CI: 9·8-10·3). Indicators of socioeconomic deprivation were strongly associated with an increase in stillbirth: rural residence (aOR:1·27,95%CI:1·16-1·39), female illiteracy (aOR:1·43,95%CI 1·17-1·74); low socioeconomic status (aOR:2·42, 95%CI:1·82-3·21); schedule caste background (aOR:1·11,95%CI:1·04-1·19), and woman not in paid employment (aOR:1·15,95%CI:1·07-1·24). Women from minority religious groups were at higher risk than the Hindu majority (Muslim (aOR:1·33,95%CI:1·25-1·43); Christian (aOR:1·42,95%CI:1·19-1·70)). While few women smoked (<1%), around 9% reported chewing tobacco, which was associated with an increased odds of stillbirth (aOR:1·11,95%CI:1·02-1·21). Adverse pregnancy and birth characteristics were also associated with stillbirth: antenatal care visits <4 (aOR:1·08,95%CI:1·01-1·15), maternal age <25 years (aOR:1·29,95%CI:1·21-1·37) and ≥35 years (aOR:1·16,95%CI:1·04-1·29), multigravida (aOR:3·06,95%CI:2·42-3·86), multiple pregnancy (aOR:1·77,95%CI:1·47-2·15), assisted delivery (aOR:3·45,95%CI:3·02-3·93), caesarean section (aOR:1·73,95%CI:1·58-1·89), as were pregnancy complications (aOR 1.42, 95%CI:1.33 – 1.51).

Conclusion: India is an emerging market economy experiencing a rapid health transition, yet these findings demonstrate the marked disparity in risk of stillbirth by women's socioeconomic status. Tobacco chewing, maternal and fetal complications were each found to be important modifiable risk factors. Targeting the 'at-risk' population identified here, improved recording of stillbirths and the introduction of local reviews would be important steps to reduce the high burden of stillbirths in India.

ARTICLE SUMMARY

Strengths and limitations of this study

- India has the highest number of stillbirths, globally. This study identifies the characteristics of high risk women and key risk factors in the Indian context using the largest available dataset

For peer review only - http://bmjopen.bmj.com/site/about/guidelines.xhtml

- The data were drawn from Indian Annual Health Survey (2010-2013), which sampled women from the nine states that account for 50% of the country's population, using methods designed to minimise selection bias.
- This study analyses a large sample (>800,000 women), providing sufficient statistical power to conduct a robust examination of a wide range of risk factors.
- The findings are generalizable for high burden states included in this study, but may not be generalizable to the rest of India.
- However, retrospective data collection can lead to recall bias, and there is the potential for some under-reporting of stillbirth.

For peer review only

Key words: Stillbirth, Risk factors, Pregnancy complications, India

Word count: 3527

For peer review only - http://bmjopen.bmj.com/site/about/guidelines.xhtml

INTRODUCTION

Stillbirth is an important global health problem affecting over 7000 families every day and is associated with emotional, social and economic consequences.[1] In 2015 the stillbirth rate (SBR) was 18.4 per 1000 total births worldwide.[1] The progress in reducing stillbirth since 1990 has been slower than reductions in neonatal and under-five child mortality.[2] Currently, 98% of stillbirths occur in low-to-middle-income countries (LMICs)[1] and India has the highest number of stillbirths, with an estimated 592,100 deaths per year[3], and a World Health Organisation (WHO) estimated rate of 22 per 1000 total births. The Government of India has developed an Indian Newborn Action Plan (INAP) which includes efforts to reduce stillbirths to <10 per 1,000 births by 2030".[4] A modest reduction in India's SBR would translate into thousands of lives saved.

While previous studies have examined the immediate pregnancy-related risk factors for stillbirth such as infections during pregnancy and hypertensive disorders,[1 5] knowledge about distal risk factors such as socioeconomic, lifestyle related and co-morbidities is limited.[1] The Indian government recognises the need to improve pregnancy care and institutional delivery among disadvantaged socioeconomic groups who have a higher risk of maternal and fetal death.[6] Since 2005, the government has made several efforts including cash assistance and dedicated services through community health workers with a stronger focus in the states with poor health and development indicators.[6] The objective of this study was to understand the current disparities in the risk of stillbirth in these 'high focus' states by examining the association between socioeconomic, bio-demographic, behavioural, and pregnancy-specific risk factors and stillbirth in nine states in India.

METHODS

We conducted an analysis of secondary data from India's Annual Health Survey (2010-13). The survey covers nine states (Madhya Pradesh, Chattisgarh, Rajasthan, Uttarakhand, Jharkhand, Odisha, Bihar, Assam, Uttar Pradesh) which account for about 50% of the country's population.[7] A total of 886,505 women for whom information about the outcome of their last pregnancy (live birth or stillbirth) was available and whose pregnancy lasted more than seven completed months (~28 weeks' gestation) were included. Figure-1 illustrates how the sample for this study was derived.

Based on the outcome of last pregnancy, women were divided into two groups, those with a livebirth and those with a stillbirth. The outcome data is from the Women schedule (Section-

For peer review only - http://bmjopen.bmj.com/site/about/guidelines.xhtml

1) that was implemented during the baseline round of the Annual Health Survey (AHS) in 2010-11. Ever married women in the age group 15-49 years were asked about the outcome of their last pregnancy during the reference period 01-January-2007 to 31-December-2009. The numerator 'stillbirth' and denominator 'total birth = stillbirth + livebirth) is from the same reference period of the survey data. Potential risk factors for stillbirth were grouped as: socioeconomic, behavioural, and bio-demographic based on a review of published literature. The description of variables included in each group is provided in Table-1. A directed acyclic graph was used to construct a theoretical framework of relationships between the aforementioned risk factors and stillbirth (Figure-2). In addition, in a sub-sample of the population (n=668,892), we examined the association of stillbirth with the following self-reported problems/ complications during pregnancy: anaemia, eclampsia, other hypertensive disorders, antepartum haemorrhage, intrapartum haemorrhage, abnormal fetal position, breech presentation, and obstructed labour.

Table 1: Description of the variables used to examine the risk factors for stillbirth

**Risk factors**	**Description of the variables**
**Proximal or Bio-Demographic factors**	
**Fetal factors**	
Sex	Sex of the foetus was coded as either female or male
Multiple pregnancy	Number of foetuses: Women were categorised as singleton (one foetus) or multiple pregnancy (twins or higher-order multiple gestations)
**Maternal factors**	
Maternal age	Maternal age at the time of the survey was divided into five year age bands: <20, 20-24, 25-29, 30-34, 35-39, 40-44, 45 and above
Gravidity	Gravidity (number of pregnancies) was used as a proxy for parity (number of deliveries) for which information was not available· Gravidity was categorised as "first" if the index pregnancy was the first pregnancy, "second - fourth", and "five or more"·
Any complication during pregnancy	Women who reported to have any of the following complications during their index pregnancy we coded as 'yes', otherwise 'no': anaemia, eclampsia, other hypertensive disorders, antepartum haemorrhage, intrapartum haemorrhage, abnormal fetal position, breech presentation, and obstructed labour
Mode of delivery	This was categorised into vaginal delivery, assisted vaginal delivery and Caesarean section
**Intermediate factors or behavioural factors**	
**Lifestyle factors**	
Smoking tobacco	Women were asked about smoking and chewing tobacco during the interview; current practice was coded as "yes", while women who never practiced or no longer practiced were coded as "no"
Chewing tobacco	
**Health seeking behaviour**	
Antenatal care visits	Number of antenatal care (ANC) visits was categorised as '≥4 visits' (which is recommended as adequate by the WHO), and '<4 visits'
Timing of first ANC visit	Timing of the first ANC visit was categorised as 'visit in the first trimester

For peer review only - http://bmjopen.bmj.com/site/about/guidelines.xhtml

	(or first three months), which is recommended as adequate by the WHO, and 'after first three months'
Place of delivery	Place of delivery was grouped as "medical facility" or "home"
**Distal or Socioeconomic factors**	
**Social factors**	
Religion	Women were categorised into the following religious groups: 'Hindu', 'Muslim', 'Christian', and 'Others' which included several groups with small numbers such as Sikh, Buddhists, etc
Place of Residence	Place of residence was grouped into urban and rural
Social group	Women were categorised into the following social groups: 'Schedule caste (SC)', 'Schedule tribe (ST)' and 'Others'<br>○ SC and ST are officially designated groups of historically disadvantaged populations in India<br>○ 'Others' included the general social class and other backward classes
**Economic factors**	
Education	Maternal education at the time of the survey was categorised into: illiterate, primary school or below, secondary school, tertiary and above
Occupation	Based on occupational status, women were grouped as 'being in paid employment' or 'not in paid employment'
Economic status	Asset index was used to measure the economic status of the participants· Asset index scores were calculated and the study sample was divided into quintiles ranging from the lowest (quintile-1) to highest (quintile-5) socioeconomic status
**Pregnancy complication**	
Anaemia during pregnancy	This is a derived variable based on questions related to the following signs and symptoms: paleness, giddiness, weakness, excessive fatigue

Statistical analysis

Overall and state-specific rates of stillbirth per 1000 total births with 95% confidence intervals (CIs) were calculated in the study population. The characteristics of women who had a stillbirth were compared with those who had a livebirth. A univariable logistic regression analysis was performed to investigate the association between each potential risk factor and stillbirth. Maternal age was tested for linearity which showed the presence of a non-linear association with stillbirth (Figure-3); it was therefore used as a categorical variable. A multivariable model was built using a step-wise forward regression, including risk factors that were statistically significantly associated with stillbirth during univariable analysis (based on a Wald p-value of <0.05). The order in which the variables were added to the model, distal followed by intermediate then proximal factors, was informed by the theoretical framework (Figure-2). Three variables, 'smoking tobacco', 'place of delivery' and 'timing of

For peer review only - http://bmjopen.bmj.com/site/about/guidelines.xhtml

For peer review only

first ANC visit' were not statistically significantly associated with stillbirth at $p<0.05$ during the model building process, and were therefore dropped from the final models.

Calculated pairwise correlation coefficients did not show any significant collinearity between the risk factors. The following interactions were identified a priori and tested for significance using Wald test, which is a method of choice for survey-weighted data: Asset index and gravidity, place of residence and place of delivery. We found a significant interaction between 'Gravidity' (number of pregnancies) and 'Asset index'. The final regression model was adjusted for this by fitting an interaction term. We conducted further analysis to examine the association between gravidity stratified by the quintiles of the Asset index.

In a sub-sample of the study population with complete data on complications during pregnancy and medical co-morbidities, we conducted univariable logistic regression analysis to investigate their individual association with stillbirth. Statistically significant associations were further examined using multivariable logistic regression models that adjusted for socioeconomic, behavioural and bio-demographic risk factors found to be significantly associated with stillbirth. We compared the proportion of stillbirth in the subsample with the excluded group and the total sample which showed that in all three groups the proportion of stillbirth was about 1% and livebirth 99%. This suggests that the subsample for the specific pregnancy complication analysis was not a biased sample.

Missingness was investigated and data were assumed to be 'Missing at Random'. Three methods were used to address bias due to missing data; missing indicator method, complete case analysis, and multiple imputations.[8] The 'missing indicator' model in which missing data were grouped as a separate category was used as the final model. However, to maintain model stability, for variables that had <1% missing data, a separate category for 'missing' was not generated. All analyses accounted for the stratified, clustered nature of the data and were conducted using the survey command (svy) in Stata V.13.1, SE (StataCorp, College Station, Texas, USA). The proportions, means and confidence intervals presented here are weighted for design effects and non-response.

Study power

The fixed sample had more than 90% power to detect an OR of ≥1.11 or ≤0.91 associated with stillbirth at $p<0.05$ (two-tailed) for the risk factor with the highest prevalence (83% for rural residence) and an OR of ≥1.39 or ≤0.67 for the risk factor with the lowest prevalence in the study population (current smoker 0.8%).

Patient and Public Involvement

For peer review only - http://bmjopen.bmj.com/site/about/guidelines.xhtml

This is not applicable since this was a secondary analysis of anonymous survey data.

RESULTS

Rate of Stillbirth

Of the 886,505 women included in the analyses 8,429 reported a stillbirth, giving an overall rate of 10 stillbirths (95%CI 9.8-10.3) per 1000 total births. The rates of stillbirth per state are shown in Table-2 and Figure-4.

Table 2: Number and rate of stillbirths in the study population in nine states in India

State	Stillbirths	Total births	Stillbirth rate per 1000 total birth (95% CI)
Assam	856	64 841	12·8 (11·8-13·9)
Bihar	2 833	250 609	11·3 (10·9-11·8)
Chhattisgarh	476	98 220	5·0 (4·5-5·5)
Jharkhand	657	60 153	10·6 (9·7-11·6)
Madhya Pradesh	615	145 552	4·2 (3·8 - 4·6)
Odisha	534	46 162	10·9 (9·9-12·0)
Rajasthan	413	52 537	7·1 (6·4-8·0)
Uttar Pradesh	1 813	131 324	14·8 (14·1-15·6)
Uttarakhand	233	37 485	7·7 (6·5-9·2)
Overall	8 430	886 505	10·0 (9·8-10·3)

Frequencies are unweighted (true counts). Rates are weighted for design effects and non-response

Association between socioeconomic, behavioural, and bio-demographic characteristics and stillbirth

The median age for women in the study population was 26 years (Interquartile range 23-30). The frequency, proportions and association with stillbirth (unadjusted and adjusted OR) for socioeconomic, behavioural and bio-demographic variables are shown in Table 3, and pregnancy-related variables in Table 4. After adjusting for other risk factors, all examined socioeconomic factors were found to be significantly associated with stillbirth. Women living in rural settings had 27% higher odds of stillbirth compared with urban settings. Belonging to a Schedule caste social group was associated with 11% higher odds compared with the reference 'other' social group, but 26% lower odds of stillbirth for women belonging to the Schedule tribe group. Compared with Hindus, belonging to Muslim and Christian religious groups was associated with increased odds of stillbirth. Compared with women with university education or higher, women with no school education had 43% higher odds. Women not in paid employment were 15% more likely to have a stillborn baby compared with women in paid employment.

Chewing tobacco was associated with 11% higher odds of stillbirth after adjusting for other risk factors in the study population. Health seeking behaviours were significantly associated

For peer review only - http://bmjopen.bmj.com/site/about/guidelines.xhtml

For peer review only

with stillbirth. Women attending <4 antenatal care (ANC) visits had 8% higher odds compared with women who had ≥4 ANC visits.

All examined demographic and pregnancy-related factors were also found to be significantly associated with stillbirth in the study population. After full adjustment, the non-linear (U-shaped) association between mother's age and stillbirth persisted. Multiple pregnancies were associated with 77% higher odds of stillbirth compared with singleton. Pregnancies with a male fetus had 26% higher odds compared with a female fetus. Women who had an assisted delivery or caesarean section were more likely to report a stillbirth compared with women who had a normal delivery.

We found a significant statistical interaction between 'gravidity' and 'Asset index'. A stratified analysis showed that multigravida women had higher odds of stillbirth and this association was substantially stronger for women who belonged to the higher Asset Index quintiles (Table-5). The results of sensitivity analyses (complete case analysis and multiple imputations) were not materially different from the 'missing indicator' model (Supplementary Table-S1).

For peer review only

For peer review only - http://bmjopen.bmj.com/site/about/guidelines.xhtml

Table 3: The Association between stillbirth and socioeconomic behaviouraland bio-demographic factors: frequencies, unadjusted and adjusted Odds Ratios

Variables	Total births N=886 505 Frequency (%)	Stillbirths N= 8 429 Frequency (%)	Live births, N= 878 076 Frequency (%)	Unadjusted Odds Ratios (95%CI[2])	Adjusted Odds Ratios[1] (95%CI[2])
**Maternal Age (years)**					
15-19	33 482 (3·7)	436 (4·9)	33 046 (3·7)	1·52 (1·35-1·72)	1·76 (1·55-2·00)
20-24	324 130 (36·0)	3 079 (36·2)	321 051 (36·0)	1·17 (1·09-1·24)	1·29 (1·21-1·37)
25-29	301 330 (33·7)	2 546 (29·2)	298 784 (33·7)	1·00 (Ref)	1·00 (Ref)
30-34	143 529 (16·6)	1 400 (17·1)	142 129 (16·6)	1·20 (1·11-1·29)	1·05 (0·97-1·14)
35-39	55 893 (6·6)	643 (8·2)	55 250 (6·6)	1·43 (1·29-1·59)	1·16 (1·04-1·29)
40-45	20 006 (2·4)	244 (3·4)	19 762 (2·4)	1·63 (1·39-1·91)	1·29 (1·09-1·51)
45-49	8 135 (1·0)	81 (1·1)	8 054 (1·0)	1·24 (0·96-1·60)	1·04 (0·80-1·35)
**Place of Residence**					
Urban	123 099 (16·4)	874 (12·7)	122 225 (16·4)	1·00 (Ref)	1·00 (Ref)
Rural	763 406 (83·7)	7 555 (87·4)	755 851 (83·6)	1·35 (1·24-1·47)	1·27 (1·16 - 1·39)
**Religion**					
Hindu	734 134 (81·5)	6 522 (75·3)	727 612 (81·5)	1·00 (Ref)	1·00 (Ref)
Muslim	125 723 (15·9)	1 619 (21·6)	124 104 (15·8)	1·48 (1·39-1·58)	1·33 (1·25-1·43)
Christian	14 914 (1·8)	178 (2·4)	14 736 (1·8)	1·49 (1·25-1·78)	1·42 (1·19-1·70)
Others	11 734 (0·9)	110 (0·7)	11 624 (0·9)	0·87 (0·69-1·11)	1·06 (0·84-1·35)
**Social Group**					
Other	598 477 (68·4)	5 868 (70·0)	592 609 (68·4)	1·00 (Ref)	1·00 (Ref)
Schedule Caste	161 273 (19·2)	1 644 (21·4)	159 629 (19·2)	1·09 (1·02-1·16)	1·11 (1·04-1·19)
Schedule Tribe	126 755 (12·4)	917 (8·7)	125 838 (12·5)	0·68 (0·62-0·74)	0·74 (0·68-0·81)
**Maternal Education**					
Tertiary and above	36 617 (3·9)	188 (2·2)	36 429 (4·0)	1·00 (Ref)	1·00 (Ref)
Secondary	110 458 (11·3)	786 (8·2)	109 672 (11·3)	1·31 (1·08-1·60)	1·10 (0·90-1·34)
Primary and below	364 809 (39·1)	3 413 (38·1)	361 396 (39·1)	1·77 (1·48-2·12)	1·34 (1·10-1·62)
Illiterate	374 621 (45·7)	4 042 (51·6)	370 579 (45·6)	2·06 (1·72-2·47)	1·43 (1·17-1·74)
**Employment**					
In paid employment	154 177 (16·4)	1 273 (13·2)	152 904 (16·4)	1·00 (Ref)	1·00 (Ref)
Not in-paid employment	732 328 (83·6)	7 156 (86·8)	725 172 (83·6)	1·29 (1·20-1·38)	1·15 (1·07-1·24)
**Asset Index, Quintiles**					
5 “Highest”	159 957 (17·9)	1 133 (13·6)	158 824 (17·9)	1·00 (Ref)	1·00 (Ref)
4	160 335 (17·6)	1 422 (16·9)	158 913 (17·6)	1·26 (1·14-1·38)	1·34 (1·01-1·79)
3	159 567 (18·0)	1 606 (19·3)	157 961 (18·0)	1·41 (1·29-1·55)	1·91 (1·45-2·52)
2	157 506 (17·9)	1 654 (19·8)	155 852 (17·8)	1·46 (1·33-1·60)	2·45 (1·85-3·24)
1 “Lowest”	155 283 (18·0)	1 626 (18·9)	153 657 (17·9)	1·39 (1·26-1·52)	2·42 (1·82-3·21)
Missing	93 857 (10·7)	988 (11·5)	92 869 (10·7)	1·41 (1·27-1·56)	1·21 (0·91-1·62)
**Current Smoking[3]**					
No	725 560 (82·0)	6 670 (79·6)	718 890 (82·0)	1·00 (Ref)	-
Yes	6 553 (0·8)	80 (1·2)	6 473 (0·8)	1·53 (1·19-1·97)	-
Missing	154 392 (17·2)	1 679(19·3)	152 713 (17·2)	1·16 (1·08-1·23)	-

**Chewing Tobacco**[3]					
No	635 393 (74·1)	5 743 (71·5)	629 650 (74·1)	1·00 (Ref)	1·00 (Ref)
Yes	96 806 (8·7)	1 010 (9·3)	95 796 (8·7)	1·10 (1·01-1·19)	1·11 (1·02-1·21)
Missing	154 306 (17·2)	1 676 (19·2)	152 630 (17·2)	1·16 (1·09-1·24)	1·44 (1·29-1·59)

[1] Multivariable logistic regression, adjusting for the other variables in the model –except self-reported mental illness. The model also adjusts for the observed significant interaction between Gravidity and Asset Index. Smoking was not significantly associated with stillbirth during the model building process and therefore, removed from the final model.

[2] The 95% confidence intervals were calculated using linearized standard errors

[3] These variables reflect practice at the time of the survey

Frequencies are true counts, % and OR are weighted for design effects and non-response

For peer review only

For peer review only - http://bmjopen.bmj.com/site/about/guidelines.xhtml

Table 4: The Association between stillbirth and pregnancy-related factors: frequencies, unadjusted and adjusted Odds Ratios

Variables	Total births N=886 505 Frequency (%)	Stillbirths N= 8 429 Frequency (%)	Live births N= 878 076 Frequency (%)	Unadjusted Odds Ratios (95%CI[2])	Adjusted Odds Ratios[1] (95%CI[2])
**Number of antenatal care visit**					
4 or more	269 148 (28·9)	2 222 (24·6)	266 926 (29·0)	1·00 (Ref)	1·00 (Ref)
Less than 4	518 288 (58·7)	4 939 (59·3)	513 349 (58·6)	1·19 (1·12-1·26)	1·08 (1·01-1·15)
Missing	99 069 (12·4)	1 268 (16·1)	97 801 (12·4)	1·53 (1·41-1·66)	1·36 (1·25-1·48)
**Timing of the first antenatal care visit**					
Three or less	534 118 (58·6)	4 678 (54·0)	529 440 (58·7)	1·00 (Ref)	-
More than three	253 460 (28·9)	2 483 (29·9)	250 977 (28·9)	1·12 (1·06-1·19)	-
Missing	98 927 (12·4)	1 268 (16·1)	97 659 (12·4)	1·41 (1·31-1·52)	-
**Place of delivery**					
Medical facility	539 704 (60·5)	5 176 (61·9)	534 528 (60·5)	1·00 (Ref)	-
Home	346 801 (39·5)	3 253 (38·1)	343 548 (39·5)	0·94 (0·89-0·99)	-
**Number of pregnancies**					
1	158 886 (16·9)	957 (10·6)	157 929 (16·9)	1·00 (Ref)	1·00 (Ref)
2-4	414 938 (46·8)	4 409 (51·8)	410 529 (46·7)	1·77 (1·63-1·93)	3·06 (2·42-3·86)
5 or more	93 458 (11·7)	1 404 (19·0)	92 024 (11·7)	2·60 (2·36-2·87)	4·98 (3·66-6·74)
Missing	219 253 (24·7)	1 659 (18·6)	217 594 (24·7)	1·21 (1·10-1·32)	1·40 (0·68-2·90)
**Sex of the baby**					
Female	414 913 (46·6)	3 468 (41·2)	411 445 (46·7)	1·00 (Ref)	1·00 (Ref)
Male	471 592 (53·4)	4 961 (58·9)	466 631 (53·3)	1·25 (1·19-1·32)	1·26 (1·20 - 1·33)
**Multiple gestations (index pregnancy)**					
Singleton	878 556 (99·1)	8 267 (98·2)	870 289 (99·1)	1·00 (Ref)	1·00 (Ref)
Twin pregnancy	7 949 (0·9)	162 (1·8)	7 787 (0·9)	2·03 (1·67-2·45)	1·77 (1·47 - 2·15)
**Mode of delivery**					
Spontaneous vaginal	805 430 (90·7)	7 146 (84·3)	798 284 (90·8)	1·00 (Ref)	1·00 (Ref)
Assisted vaginal	14 909 (1·8)	390 (5·0)	14 519 (1·7)	3·13 (2·76-3·55)	3·45 (3·02-3·93)
Caesarean section	66 166 (7·6)	893 (10·7)	65 273 (7·5)	1·53 (1·40-1·66)	1·73 (1·58-1·89)
**Any pregnancy complications**					
No	250 099 (28·1)	2 000 (23·5)	250 099 (28·1)	1·00 (Ref)	1·00 (Ref)
Yes	418 856 (47·5)	4 779 (58·0)	414 077 (47·4)	1·47 (1·38-1·56)	1·42 (1·33-1·51)
Missing	217 550 (24·5)	1 650 (18·5)	215 900 (24·5)	0·91 (0·84-0·98)	1·99 (1·00-3·94)

[1] Multivariable logistic regression, adjusting for the other variables in the model –except self-reported mental illness. The model also adjusts for the observed significant interaction between Gravidity and Asset Index.
[2] The 95% confidence intervals were calculated using linearized standard errors
Frequencies are true counts, % and OR are weighted for design effects and non-response

For peer review only - http://bmjopen.bmj.com/site/about/guidelines.xhtml

Table 5: Adjusted[1] odds ratios and 95%CI for the association between gravidity and stillbirth stratified by the Asset index quintiles

**A· Missing Indicator Method**					
	**Asset Index Quintiles**				
	**1 (most deprived)**	**2**	**3**	**4**	**5 (least deprived)**
Gravidity					
1	1·00 (Ref)	1·00 (Ref)	1·00 (Ref)	1·00 (Ref)	1·00 (Ref)
2-4	1·44 (1·15-1·81)	1·47 (1·18-1·84)	1·83 (1·48 - 2·26)	2·55 (2·02 – 3·23)	3·04 (2·37 – 3·90)
More than 5	1·89 (1·43-2·49)	1·88 (1·43-2·48)	3·04 (2·34 - 3·94)	3·71 (2·75 - 5·02)	4·62 (3·27 - 6·54)
Missing	0·95 (0·74-1·22)	1·18 (0·92-1·50)	1·37 (1·09-1·72)	1·88 (1·44 – 2·45)	2·26 (1·67 – 3·05)
**B· Complete Case Analysis**					
	**Asset Index Quintiles**				
	**1**	**2**	**3**	**4**	**5**
Gravidity					
1	1·00 (Ref)	1·00 (Ref)	1·00 (Ref)	1·00 (Ref)	1·00 (Ref)
2-4	1·19 (0·92-1·54)	1·22 (0·94-1·60)	1·74 (1·35 - 2·24)	2·05 (1·56 – 2·67)	2·86 (2·11 – 3·88)
More than 5	1·59 (1·15-2·18)	1·48 (1·05-2·08)	2·70 (1·97 - 3·73)	3·02 (2·12 - 4·31)	5·02 (3·35 - 7·53)
**C· Multiple Imputations**					
	**Asset Index Quintiles**				
	**1**	**2**	**3**	**4**	**5**
Gravidity					
1	1·00 (Ref)	1·00 (Ref)	1·00 (Ref)	1·00 (Ref)	1·00 (Ref)
2-4	1·48 (0·90-2·48)	1·50 (0·90-2·55)	1·77 (1·08-2·92)	2·29 (1·39-3·84)	2·73 (2·20 – 3·40)
More than 5	1·90 (1·00-3·52)	1·98 (1·07-3·81)	2·69 (1·40-5·04)	3·31 (1·70-6·45)	4·41 (3·33 - 5·86)

[1]Multivariable logistic regression, adjusting for socioeconomic, health seeking behaviour, pre-existing medical conditions, and bio-demographic characteristics
Results are weighted for design effects and non-response

For peer review only

For peer review only - http://bmjopen.bmj.com/site/about/guidelines.xhtml

Association between specific complications during pregnancy and stillbirth

Reporting any pregnancy complication was associated with significantly higher odds of stillbirth after adjusting for the identified socioeconomic, behavioural, and bio-demographic risk factors (Table-4). The association between each of the reported complication and stillbirth are shown in Table-6. Women who reported to have anaemia during pregnancy had 35% higher odds of stillbirth compared with women who did not have anaemia. Eclampsia was associated with almost twice the odds of stillbirth and other hypertensive disorders were associated with about 22% higher odds. Women who had an ante-partum or intra-partum haemorrhage had significantly higher odds of stillbirth compared with women who did not have these complications. The adjusted odds of stillbirth were 58% higher in women with abnormal fetal position, more than three times higher for women who had an obstructed labour, and nearly three times higher for women who had a breech presentation compared with women who did not report these complications. We also noted that the association between stillbirth and caesarean section was no longer significant at $p<0.05$ and the aOR for assisted delivery was significantly attenuated in the model that examined the association between obstructed labour and stillbirth. Sensitivity analysis conducted using complete case analysis and multiple imputations did not change the results materially (Supplementary Table-S2).

For peer review only - http://bmjopen.bmj.com/site/about/guidelines.xhtml

Table 6: Association between stillbirth and complications during pregnancy–frequencies, unadjusted and adjusted Odds Ratios (95% CI)

Variables	Total births N=668,892 Frequency (%)	Stillbirths N= 6,777 Frequency (%)	Live births, N= 662,115 Frequency (%)	Unadjusted Odds ratios (95%CI)	Adjusted Odds ratios (95%CI)
**Anemia**					
No	500 785 (73.8)	4 611 (66.0)	496 174 (73.9)	1 (Ref)	1 (Ref)
Yes	168 107 (26.2)	2 166 (34.0)	165 941 (26.1)	1·45 (1·36-1·54)	1·35 (1·27-1·43)
**Eclampsia**					
No	628 681 (93.8)	6 043 (88.9)	622 638 (93.8)	1 (Ref)	1 (Ref)
Yes	40 211 (6.2)	734 (11.2)	39 477 (6.2)	1·90 (1·73 -2·10)	1·79 (1·62 -1·97)
**Other hypertensive disorders**					
No	639 689 (95.5)	6 412 (94.7)	633 277 (95.5)	1 (Ref)	1 (Ref)
Yes	29 203 (4.5)	365 (5.3)	28 838 (4.5)	1·20 (1·05-1·36)	1·22 (1·07-1·38)
**Intra-partum Hemorrhage**					
No	622 206 (93.3)	5 559 (82.6)	616 647 (93.4)	1 (Ref)	1 (Ref)
Yes	46 686 (6.7)	1 218 (17.4)	45 468 (6.6)	2·97 (2·75 -3·21)	2·75 (2·54 -2·97)
**Ante-partum Hemorrhage**					
No	650 631 (97.2)	6 488 (96.0)	644 143 (97.2)	1 (Ref)	1 (Ref)
Yes	18 261 (2.8)	289 (4.0)	17 972 (2.8)	1·44 (1·25-1·67)	1·29 (1·11-1·50)
**Obstructed Labor**					
No	602 184 (90.1)	5 037 (73.4)	597 147 (90.2)	1 (Ref)	1 (Ref)
Yes	66 708 (9.9)	1 740 (26.6)	64 968 (9.8)	3·35 (3·13-3·58)	3·45 (3·19-3·74)
**Breech presentation**					
No	645 162 (96.2)	6 092 (89.1)	639 070 (96.3)	1 (Ref)	1 (Ref)
Yes	23 730 (3.8)	685 (10.9)	23 045 (3.7)	3·18 (2·88-3·50)	2·80 (2·51-3·12)
**Abnormal Fetal position**					
No	640 015 (95.4)	6 269 (92.5)	633 746 (95.5)	1 (Ref)	1 (Ref)
Yes	28 877 (4.6)	508 (7.5)	28 369 (4.5)	1·72 (1·54-1·92)	1·58 (1·40-1·77)

[1]Each multivariable logistic regression model adjusts for socioeconomic, health seeking behaviour, and bio-demographic characteristics identified to be significantly associated with Stillbirth in the previous model.
[2] Although the sub-population was restricted to women who had complete data on pregnancy-specific complication, the other variables adjusted for in the model had missing data, therefore sensitivity analyses were conducted.
Frequencies are true counts, % and OR are weighted for design effects and non-response

For peer review only - http://bmjopen.bmj.com/site/about/guidelines.xhtml

DISCUSSION

The overall rate of stillbirth was found to be 10 per 1000 total births, but varied widely between the nine states ranging from 4.2 to 14.8 per 1000 total births. The marked variation at the state level may be explained by the different population characteristics and the varying distribution of risk factors across the nine states. The findings show the impact of inequality on stillbirth, as women in the most deprived groups are at highest risk. This was identified across a number of variables which capture different aspects of deprivation; for example, poorer women, those with little education, those living in rural areas, not currently working and belonging to 'schedule caste' groups are at increased risk of stillbirth compared to more affluent and advantaged women. Yet, among the multigravidas odds of stillbirth were substantially higher in the more affluent group, after adjusting for other factors. Chewing tobacco is highlighted as a significant risk factor for stillbirth, which is important in the Indian context where few women report tobacco smoking. Pregnancy complications, including anaemia, eclampsia, other hypertensive disorders, antepartum haemorrhage, intrapartum haemorrhage, abnormal fetal position, breech presentation, and obstructed labour significantly increased the odds of stillbirth.

The overall estimated stillbirth rate in the study population from the nine states in India was approximately half that of the World Health Organisation (WHO) estimated rate of 22 per 1000 total births. This was also true for the rate of stillbirth for the state of Bihar (11.3 per 1000 births; 95% CI = 10.9-11.8 reported in Table 2) when compared with the rate estimated for this state by another study that used both household survey and verbal autopsy to estimate the rate (21.2 per 1,000 births; 95% CI 19.7 to 22.6).[9] However, the risk factors for stillbirth identified by our study is consistent with the findings of the other study by Dandona et al.[9] Possible explanations for the low stillbirth rate include under-reporting of stillbirths or classifying stillbirths as neonatal deaths due to associated stigma. It is also important to acknowledge that the rates of stillbirth from the WHO, from Dandona et al's study[9] and from our study are all estimates, and it is difficult to confirm which estimate is the most accurate.

We found a significant disparity in the risk of stillbirth by socioeconomic status which is consistent with previous research. A systematic review of studies from developing countries showed that low socioeconomic status was significantly associated with stillbirth with a population attributable fraction ranging between 2% and 75%.[10] A study from 13 European countries reported that lower maternal education and maternal unemployment were associated with 1.9 and 1.6 times higher odds of stillbirth, respectively.[10] Maternal

For peer review only - http://bmjopen.bmj.com/site/about/guidelines.xhtml

education and employment may act through promoting high self-esteem and empowering women to make decisions about healthcare utilisation.[11]

The increased odds of stillbirth in women belonging to religious minority groups in India could be due to cultural constraints reported in other studies or inequalities that minority groups are subjected to which influences their healthcare seeking behaviours, even in countries that have universal access to medical care.[12] In India, Schedule tribe and Schedule caste social groups have been historically disadvantaged, belonging to a Schedule caste group was associated with higher odds of stillbirth which is consistent with previous literature of disadvantaged groups having higher risk.[13] However, women belonging to the Schedule tribe group had lower odds of stillbirth. While, it might be possible that individuals belonging to Schedule tribe groups have different health seeking behaviours leading to a lower risk,[14 15] other factors, not adjusted for directly in this study, could explain this difference. Examples include nutritional risk factors, exposure to environmental toxin through occupation or household (such as indoor air pollution) or exposure to domestic violence,

Self-reported tobacco smoking or chewing has been shown to be associated with increased risk of stillbirth in other studies.[16 17] In the study population, chewing tobacco was 15-times more common compared with smoking tobacco and women who chewed tobacco had a 14% higher odds of stillbirth compared with women who did not. In contrast to other studies,[16 17] smoking was not found to be significantly associated with stillbirth in our study population after adjusting for other risk factors. This could be because smoking at the time of the survey may not reflect smoking during pregnancy. While smoking during pregnancy is discouraged, healthcare messages about chewing tobacco during pregnancy are less clear.

Our findings related to timing and number of ANC visits conforms to the results of other studies. A study by Chopra et al estimated that 24% of stillbirth and perinatal deaths in South Africa could be prevented every year through improved use of ANC services.[18] However another study from South Africa found that timing of booking visit may not, in isolation, be an important determinant of stillbirth.[19] Improving the quality of pregnancy care with specific measures to prevent stillbirth are important in addition to increasing coverage of antennal care. Assisted delivery and caesarean section were found to be associated with higher risk of stillbirth, but this association was grossly attenuated and no longer statistically significant for caesarean section after adjusting for obstructed labour, suggesting the presence of a reverse causation. Similar to other studies, our study showed a U-shaped association between maternal age and stillbirth[20-22] and male sex of the fetus to be

For peer review only - http://bmjopen.bmj.com/site/about/guidelines.xhtml

associated with higher odds of stillbirth.[1 23] The higher odds of stillbirth observed with multiple pregnancies compared with singleton pregnancies can be explained by propensity of women with multiple pregnancies to have more complications.[24]

A number of studies have shown that anaemia during pregnancy is associated with increased risk of stillbirth, with 3.7-16 times higher odds of stillbirth associated with anaemia among pregnant women.[25 26] A meta-analysis of risk factors in high income countries reported higher odds of stillbirth for women with pregnancy-induced hypertension, pre-eclampsia and eclampsia.[27] Antepartum and intrapartum haemorrhage can be due to a myriad of obstetric complications, a meta-analysis reported that there was a four-fold increased risk of stillbirth associated with bleeding.[28] Breech presentation, abnormal fetal position, and obstructed labour operate through similar mechanisms, the fetus is trapped in the birth canal and is subjected to hypoxia leading to stillbirth.[29] However, a stillbirth could be prevented if these complications are detected timely and managed appropriately. Previous studies suggest that women with pre-existing mental health problems have 70% higher risk of stillbirth.[30]

The Annual Health Survey is the largest health survey in the world. The large sample provided adequate number of events (stillbirths) and allowed us to conduct an adequately powered, robust examination of a wide range of risk factors. The survey is a representative sample from nine states in India with high rates of stillbirth and the sampling strategy minimised the possibility of selection bias. The findings are generalizable for high burden states included in this study, but may not be generalizable to the rest of India. To our knowledge, no other large dataset is available to investigate stillbirth in India, the country with the highest number of stillbirths.

There are some limitations of this study which should be considered when interpreting the findings. Important to consider is that stillbirths may be under-reported or misclassified in the AHS from which our data were drawn. A study by Christou et al, 2017[31] showed that stillbirths are likely to be under-reported in routine household surveys, but this was more likely to be due a lack of rigorous ascertainment of pregnancy outcomes rather than deliberate non-reporting by women due to any reason. We did not find any evidence from published literature suggesting under-reporting of stillbirth to vary by risk factors or specific population groups, thus we excluded the possibility of differential under-reporting. However, we cannot exclude the possibility of misclassification of stillbirth as miscarriage/ abortion or neonatal deaths as stillbirth. A small proportion of the stillbirths were reported as pregnancy loss/ abortion after seven months of gestation in the dataset. We reclassified these as stillbirth in our analysis.

For peer review only - http://bmjopen.bmj.com/site/about/guidelines.xhtml

Retrospective data collection could also have led to potential recall bias, and self-reported complications during pregnancy were not verified with hospital records. It was not possible to differentiate between pregnancy-induced hypertension and pre-existing essential hypertension. To minimise the effect of recall bias, dummy variables (e.g. anaemia) were generated wherever feasible and highly subjective symptoms (e.g. prolonged labour) were not investigated. Data were not available for a number of potential risk factors for stillbirth such as timing of stillbirth, thus we were not able to analyse the risk factors separately for antepartum and intrapartum stillbirths. An inherent limitation of routine survey data is missing information; however, the results of the analyses using three different methods to account for missing data were not substantially different.

Conclusion

Our study showed that despite several efforts being made to improve pregnancy care in India, socioeconomic disparities in stillbirth still exits and maternal and fetal complications were found to be important preventable risk factors. Another important finding was the risk associated with chewing tobacco which demonstrates an urgent need for strong public health messages to stop chewing tobacco during pregnancy in addition to the messages to stop smoking. Improving uptake of antenatal care, and timely identification and effective management of maternal and fetal complications could reduce preventable stillbirths. India has a large cadre of frontline healthcare workers or community health and nutrition workers called 'ASHAs' and 'Anganwadi workers'. They could play an important role in timely identification of danger signs through frequent interactions with pregnant women who are at a higher risk of stillbirth. In addition, ASHAs and Anganwadi workers are ideally placed to facilitate Information, Education and Communication (IEC) programmes to specifically target stigma around reporting of stillbirth. Eradicating poverty and promotion of female education are part of the global developmental agenda espoused by the Sustainable Development Goals, for which India is a signatory. This study showed that progress towards these goals could accelerate progress in preventing stillbirths.

Despite limitations, as highlighted by Lawn et al, 2010[32], household surveys remain the primary source of stillbirth data for low-and-middle income countries with more than 75% of the global burden of stillbirths. AHS is one of the largest household surveys in the world conducted by the Office of the Registrar General & Census Commissioner of India and is therefore an important data source for preliminary and baseline studies for generating hypothesis for further in-depth research. Studies are needed to identify risk factors separately for antepartum and intrapartum stillbirths in India. The association between the maternal and fetal complications and stillbirth suggests that although at present, pregnant

For peer review only - http://bmjopen.bmj.com/site/about/guidelines.xhtml

For peer review only

women in India are incentivised to seek antenatal care and give birth in health institutions, the quality of pregnancy care need further investigation. Currently, neither are all stillbirths recorded nor are local reviews of stillbirths conducted in the country. Targeting at-risk population groups, recording all stillbirths and conducting local reviews would be important to reduce the high burden of stillbirths in India.

REFERENCE LIST

1. Lawn JE, Blencowe H, Waiswa P, et al. Stillbirths: rates, risk factors, and acceleration towards 2030. *The Lancet* 2016;387(10018):587-603. doi: http://dx.doi.org/10.1016/S0140-6736(15)00837-5
2. Frøen JF, Friberg IK, Lawn JE, et al. Stillbirths: progress and unfinished business. *The Lancet*;387(10018):574-86. doi: 10.1016/S0140-6736(15)00818-1
3. Blencowe H, Cousens S, Jassir FB, et al. National, regional, and worldwide estimates of stillbirth rates in 2015, with trends from 2000: a systematic analysis. *The Lancet Global Health* 2016;4(2):e98-e108. doi: http://dx.doi.org/10.1016/S2214-109X(15)00275-2
4. Sharma D. India newborn action plan. *International Journal of Medical Science Research and Practice* 2015;2(2):58.
5. Di Mario S, Say L, Lincetto O. Risk Factors for Stillbirth in Developing Countries: A Systematic Review of the Literature. *Sexually Transmitted Diseases* 2007;34(7):S11-S21. doi: 10.1097/01.olq.0000258130.07476.e3
6. Ministry of Health and Family Welfare (MoHFW) GoI. National Health Portal: Janani Suraksha Yojana (JSY) Ne Delhi: National Institute of Health and Family Welfare (NIHFW), MoHFW; [cited 2017 18 December]. accessed 18 December 2017.
7. Office of Registrar General & Census Commissioner MoHA, Government of India. Annual Health Survey Report: A report on core and vital health indicators part I. 2014
8. Pedersen AB, Mikkelsen EM, Cronin-Fenton D, et al. Missing data and multiple imputation in clinical epidemiological research. *Clinical Epidemiology* 2017;9:157.
9. Dandona R, Kumar GA, Kumar A, et al. Identification of factors associated with stillbirth in the Indian state of Bihar using verbal autopsy: A population-based study. *PLOS Medicine* 2017;14(8):e1002363. doi: 10.1371/journal.pmed.1002363
10. McClure EM, Goldenberg RL. Stillbirth in Developing Countries: A review of causes, risk factors and prevention strategies. *The journal of maternal-fetal & neonatal medicine : the official journal of the European Association of Perinatal Medicine, the Federation of Asia and Oceania Perinatal Societies, the International Society of Perinatal Obstetricians* 2009;22(3):183-90. doi: 10.1080/14767050802559129
11. Pincus T, Callahan LF. Associations of low formal education level and poor health status: behavioral, in addition to demographic and medical, explanations? *Journal of clinical epidemiology* 1994;47(4):355-61.
12. Penn N, Oteng-Ntim E, Oakley LL, et al. Ethnic variation in stillbirth risk and the role of maternal obesity: analysis of routine data from a London maternity unit. *BMC pregnancy and childbirth* 2014;14(1):404.
13. Smith GCS, Fretts RC. Stillbirth. *The Lancet* 2007;370(9600):1715-25. doi: http://dx.doi.org/10.1016/S0140-6736(07)61723-1
14. Moffatt M. An untouchable community in South India: Structure and consensus: Princeton University Press 2015.
15. Navaneetham K, Dharmalingam A. Utilization of maternal health care services in Southern India. *Social science & medicine (1982)* 2002;55(10):1849-69. [published Online First: 2002/10/18]

For peer review only - http://bmjopen.bmj.com/site/about/guidelines.xhtml

16. Gupta PC, Subramoney S. Smokeless tobacco use and risk of stillbirth: a cohort study in Mumbai, India. *Epidemiology (Cambridge, Mass)* 2006;17(1):47-51. [published Online First: 2005/12/17]
17. Marufu TC, Ahankari A, Coleman T, et al. Maternal smoking and the risk of still birth: systematic review and meta-analysis. *BMC Public Health* 2015;15:239. doi: 10.1186/s12889-015-1552-5
18. Neogi SB, Negandhi P, Chopra S, et al. Risk Factors for Stillbirth: Findings from a Population-Based Case–Control Study, Haryana, India. *Paediatric and perinatal epidemiology* 2016;30(1):56-66.
19. Beauclair R, Petro G, Myer L. The association between timing of initiation of antenatal care and stillbirths: a retrospective cohort study of pregnant women in Cape Town, South Africa. *BMC Pregnancy and Childbirth* 2014;14(1):204. doi: 10.1186/1471-2393-14-204
20. Andersen A-MN, Wohlfahrt J, Christens P, et al. Maternal age and fetal loss: population based register linkage study. *BMJ* 2000;320(7251):1708-12. doi: 10.1136/bmj.320.7251.1708
21. Althabe F, Moore JL, Gibbons L, et al. Adverse maternal and perinatal outcomes in adolescent pregnancies: The Global Network's Maternal Newborn Health Registry study. *Reproductive Health* 2015;12(Suppl 2):S8-S8. doi: 10.1186/1742-4755-12-S2-S8
22. Khandait DW, Ambadekar NN, Zodpey SP, et al. Maternal age as a risk factor for stillbirth. *Indian journal of public health* 2000;44(1):28-30. [published Online First: 2001/07/07]
23. Mondal D, Galloway TS, Bailey TC, et al. Elevated risk of stillbirth in males: systematic review and meta-analysis of more than 30 million births. *BMC Medicine* 2014;12:220. doi: 10.1186/s12916-014-0220-4
24. Kurdi AM, Mesleh RA, Al-Hakeem MM, et al. Multiple pregnancy and preterm labor. *Saudi medical journal* 2004;25(5):632-7. [published Online First: 2004/05/13]
25. Lone FW, Qureshi RN, Emanuel F. Maternal anaemia and its impact on perinatal outcome. *Tropical Medicine & International Health* 2004;9(4):486-90.
26. Engelgau MM, Sampson UK, Rabadan-Diehl C, et al. Tackling NCD in LMIC: Achievements and Lessons Learned From the NHLBI–UnitedHealth Global Health Centers of Excellence Program. *Global Heart* 2016;11(1):5-15. doi: http://dx.doi.org/10.1016/j.gheart.2015.12.016
27. Flenady V, Koopmans L, Middleton P, et al. Major risk factors for stillbirth in high-income countries: a systematic review and meta-analysis. *Lancet* 2011;377 doi: 10.1016/s0140-6736(10)62233-7
28. Ananth CV, Savitz DA. Vaginal bleeding and adverse reproductive outcomes: a meta-analysis. *Paediatric and Perinatal Epidemiology* 1994;8(1):62-78. doi: 10.1111/j.1365-3016.1994.tb00436.x
29. Lawn J, Shibuya K, Stein C. No cry at birth: global estimates of intrapartum stillbirths and intrapartum-related neonatal deaths. *Bulletin of the World Health Organization* 2005;83(6):409-17.
30. Gardosi J, Madurasinghe V, Williams M, et al. Maternal and fetal risk factors for stillbirth: population based study. *Bmj* 2013;346:f108.
31. Christou A, Dibley MJ, Raynes-Greenow C. Beyond counting stillbirths to understanding their determinants in low-and middle-income countries: a systematic assessment of stillbirth data availability in household surveys. *Tropical Medicine & International Health* 2017;22(3):294-311.
32. Lawn JE, Gravett MG, Nunes TM, et al. Global report on preterm birth and stillbirth (1 of 7): definitions, description of the burden and opportunities to improve data. *BMC Pregnancy and Childbirth* 2010;10(1):S1. doi: 10.1186/1471-2393-10-s1-s1

AUTHOR STATEMENT

For peer review only - http://bmjopen.bmj.com/site/about/guidelines.xhtml

Competing interests

No, there are no competing interests for any author.

Contribution to authorship

NA reviewed the literature, conducted the analysis and wrote the first draft of the paper; CC helped with the data analysis, and edited the paper; SSC helped in acquiring and interpreting the data, and edited the paper; AR edited the paper; UCS edited the paper; MK contributed to the interpretation and discussion of the results, and edited the paper; MN developed the concept for the study, supervised the data analysis, interpretation and discussion of the results, and edited the paper.

Details of ethics approval

Ethics approval was not required since this was a secondary analysis of anonymous survey data.

Funding

The study was funded by a Medical Research Council Career Development Award to Manisha Nair (Grant Ref: MR/P022030/1). The funder had no role in the study design, data analysis, data interpretation, or writing of the manuscript. All authors, had full access to all of the data in the study and can take responsibility for the integrity of the data and the accuracy of the data analysis.

Data sharing statement

The anonymised data is freely available through the Indian Government's Data Sharing Portal.

Figure legends

Figure 1: Flowchart showing the derivation of the final study population

Figure 2: Hypothesized relationship between the risk factors and stillbirth

Figure 3: Association between maternal age in 5 year groups and stillbirth

Figure-4: Rate of stillbirth overall and by nine states in India using the Annual Health Survey data (2010-13)

For peer review only

For peer review only - http://bmjopen.bmj.com/site/about/guidelines.xhtml

Figure 1: Flowchart showing the derivation of the final study population

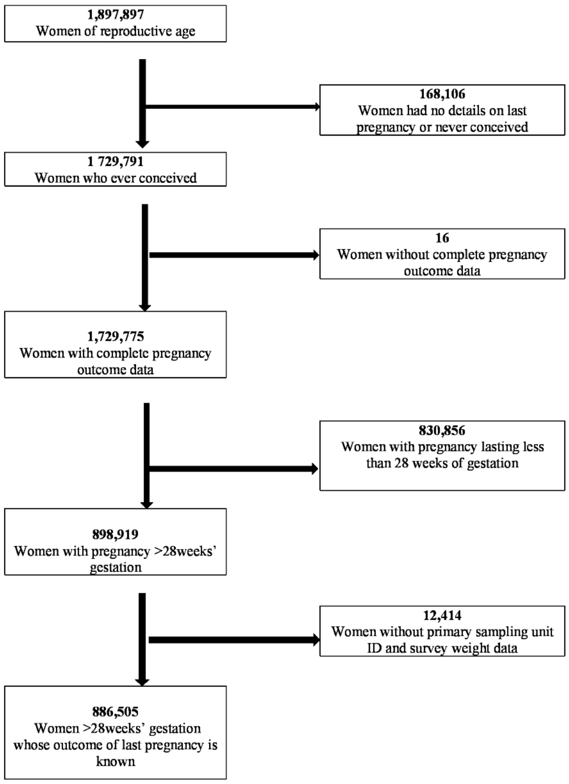

209x297mm (300 x 300 DPI)

Figure 2: Hypothesized relationship between the risk factors and stillbirth

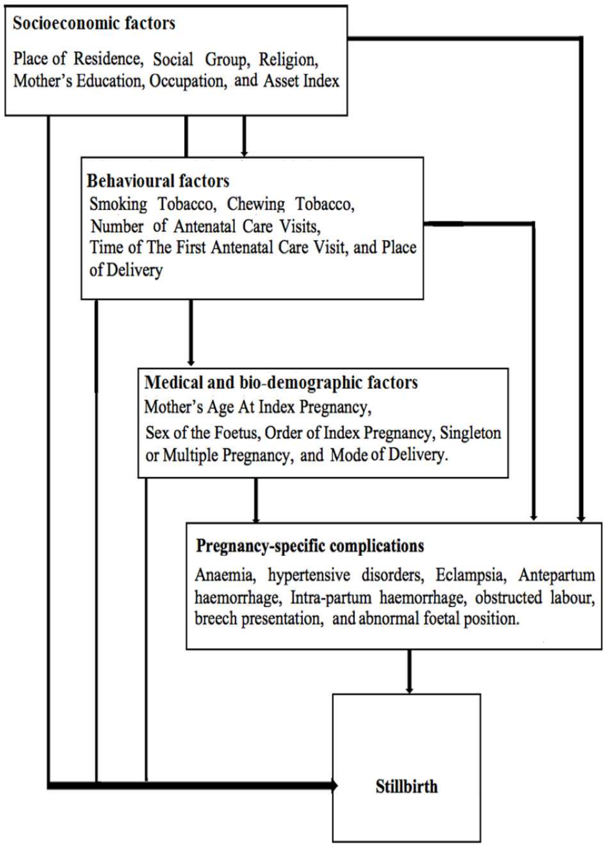

209x297mm (300 x 300 DPI)

Figure 3: Association between maternal age in 5 year groups and stillbirth

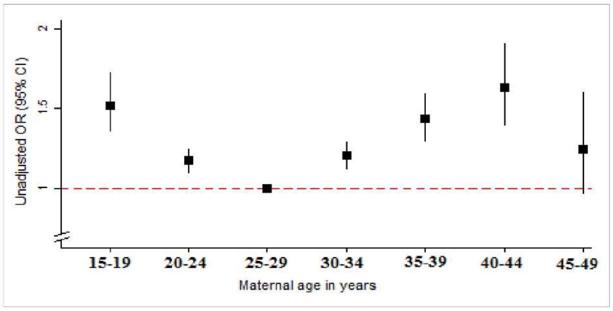

Data source: Annual Health Survey 2012-13, India

209x297mm (300 x 300 DPI)

Figure-4: Rate of stillbirth overall and by nine states in India using the Annual Health Survey data (2010-13)

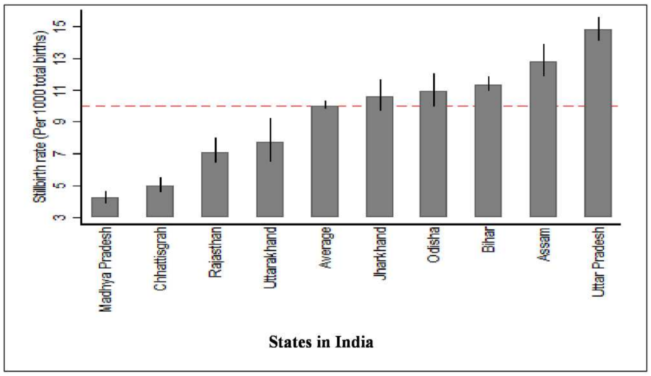

209x297mm (300 x 300 DPI)

Table-S1: Unadjusted and adjusted[1] odds ratios (OR) and 95%CI[2] for stillbirth by socioeconomic, health seeking behaviour, pre-existing medical conditions, and bio-demographic factors

Variables	Unadjusted Odds Ratios (95%CI)	Main model	Sensitivity analysis	
		Missing indicator Adjusted Odds Ratios (95%CI)	Complete case analysis Adjusted Odds Ratios (95%CI)	Multiple imputation Adjusted Odds Ratios (95%CI)
**Maternal Age (years)**				
15-19	1·52 (1·35-1·72)	1·76 (1·55-2·00)	1·58 (1·30-1·93)	1·79 (1·55 - 2·07)
20-24	1·17 (1·09-1·24)	1·29 (1·21-1·37)	1·21 (1·10-1·32)	1·29 (1·20 - 1·39)
25-29	1·00 (Ref)	1·00 (Ref)	1·00 (Ref)	1·00 (Ref)
30-34	1·20 (1·11-1·29)	1·05 (0·97-1·14)	1·13 (1·01-1·26)	1·07 (0·98 - 1·18)
35-39	1·43 (1·29-1·59)	1·16 (1·04-1·29)	1·18 (1·00-1·38)	1·18 (1·04 - 1·34)
40-45	1·63 (1·39-1·91)	1·29 (1·09-1·51)	1·42 (1·12-1·79)	1·37 (1·14 - 1·65)
45-49	1·24 (0·96-1·60)	1·04 (0·80-1·35)	1·05 (0·72-1·55)	1·05 (0·82 - 1·49)
**Place of Residence**				
Urban	1·00 (Ref)	1·00 (Ref)	1·00 (Ref)	1·00 (Ref)
Rural	1·35 (1·24-1·47)	1·27 (1·16 - 1·39)	1·31 (1·15-1·49)	1·25 (1·13 - 1·38)
**Religion**				
Hindu	1·00 (Ref)	1·00 (Ref)	1·00 (Ref)	1·00 (Ref)
Muslim	1·48 (1·39-1·58)	1·33 (1·25-1·43)	1·35 (1·22-1·49)	1·40 (1·29 - 1·51)
Christian	1·49 (1·25-1·78)	1·42 (1·19-1·70)	1·53 (1·25-1·86)	1·44 (1·20 - 1·73)
Others	0·87 (0·69-1·11)	1·06 (0·84-1·35)	0·85 (0·60-1·19)	1·09 (0·85 - 1·40)
**Social Group**				
Other	1·00 (Ref)	1·00 (Ref)	1·00 (Ref)	1·00 (Ref)
Schedule Caste	1·09 (1·02-1·16)	1·11 (1·04-1·19)	1·16 (1·05-1·28)	1·14 (1·05 - 1·23)
Schedule Tribe	0·68 (0·62-0·74)	0·74 (0·68-0·81)	0·67 (0·59-0·76)	0·71 (0·64 - 0·79)
**Maternal Education**				
Tertiary and above	1·00 (Ref)	1·00 (Ref)	1·00 (Ref)	1·00 (Ref)
Secondary	1·31 (1·08-1·60)	1·10 (0·90-1·34)	1·11 (0·84-1·47)	1·04 (0·84 - 1·30)
Primary and below	1·77 (1·48-2·12)	1·34 (1·10-1·62)	1·25 (0·95-1·64)	1·24 (1·00 - 1·53)
Illiterate	2·06 (1·72-2·47)	1·43 (1·17-1·74)	1·34 (1·02-1·77)	1·31 (1·06 - 1·62)
**Employment**				
In paid employment	1·00 (Ref)	1·00 (Ref)	1·00 (Ref)	1·00 (Ref)
Not in-paid employment	1·29 (1·20-1·38)	1·15 (1·07-1·24)	1·22 (1·10-1·36)	1·19 (1·09-1·29)
**Asset Index, Quintiles**				
5 “Highest”	1·00 (Ref)	1·00 (Ref)	1·00 (Ref)	1·00 (Ref)
4	1·26 (1·14-1·38)	1·34 (1·01-1·79)	1·58 (1·10-2·28)	1·32 (0·99-1·75)
3	1·41 (1·29-1·55)	1·91 (1·45-2·52)	2·13 (1·49-3·03)	1·81 (1·39-2·34)
2	1·46 (1·33-1·60)	2·45 (1·85-3·24)	2·64 (1·83-3·78)	2·23 (1·69-2·92)
1 “Lowest”	1·39 (1·26-1·52)	2·42 (1·82-3·21)	3·14 (2·20-4·47)	2·24 (1·71-2·94)
Missing	1·41 (1·27-1·56)	1·21 (0·91-1·62)	-	-

**Chewing Tobacco[3]**				
No	1·00 (Ref)	1·00 (Ref)	1·00 (Ref)	1·00 (Ref)
Yes	1·10 (1·01-1·19)	1·11 (1·02-1·21)	1·07 (0·97-1·19)	1·12 (1·00-1·25)
Missing	1·16 (1·09-1·24)	1·44 (1·29-1·59)	-	-
**Number of antenatal care visit**				
4 or more	1·00 (Ref)	1·00 (Ref)	1·00 (Ref)	1·00 (Ref)
Less than 4	1·19 (1·12-1·26)	1·08 (1·01-1·15)	1·11 (1·03-1·21)	1·06 (0·99-1·14)
Missing	1·53 (1·41-1·66)	1·36 (1·25-1·48)	-	-
**Timing of the first antenatal**				
**care visit**	1·00 (Ref)	-	-	-
Three or less	1·12 (1·06-1·19)	-	-	-
More than three	1·41 (1·31-1·52)	-	-	-
Missing				
**Place of delivery**				
Medical facility	1·00 (Ref)	-	-	-
Home	0·94 (0·89-0·99)	-	-	-
**Number of pregnancies**				
1	1·00 (Ref)	1·00 (Ref)	1·00 (Ref)	1·00 (Ref)
2-4	1·77 (1·63-1·93)	3·06 (2·42-3·86)	2·95 (2·20-3·94)	2·72 (2·18-3·38)
5 or more	2·60 (2·36-2·87)	4·98 (3·66-6·74)	5·35 (3·73-7·67)	4·30 (3·24-5·70)
Missing	1·21 (1·10-1·32)	1·40 (0·68-2·90)	-	-
**Sex of the baby**				
Female	1·00 (Ref)	1·00 (Ref)	1·00 (Ref)	1·00 (Ref)
Male	1·25 (1·19-1·32)	1·26 (1·20 - 1·33)	1·23 (1·14-1·32)	1·25 (1·18 - 1·32)
**Multiple gestations (index**				
**pregnancy)**	1·00 (Ref)	1·00 (Ref)	1·00 (Ref)	1·00 (Ref)
Singleton	2·03 (1·67-2·45)	1·77 (1·47 - 2·15)	1·90 (1·46-2·49)	1·84 (1·49 - 2·28)
Twin pregnancy				
**Mode of delivery**				
Spontaneous vaginal	1·00 (Ref)	1·00 (Ref)	1·00 (Ref)	1·00 (Ref)
Assisted vaginal	3·13 (2·76-3·55)	3·45 (3·02-3·93)	3·88 (3·25-4·63)	3·63 (3·16-4·18)
Caesarean section	1·53 (1·40-1·66)	1·73 (1·58-1·89)	1·75 (1·56-1·98)	1·58 (1·43-1·74)
**Any pregnancy complications**				
No	1·00 (Ref)	1·00 (Ref)	1·00 (Ref)	1·00 (Ref)
Yes	1·47 (1·38-1·56)	1·42 (1·33-1·51)	1·44 (1·33-1·56)	1·40 (1·31-1·49)
Missing	0·91 (0·84-0·98)	1·99 (1·00-3·94)	-	-

[1] Multivariable logistic regression, adjusting for the other variables in the model –except self-reported mental illness. The model also adjusts for the observed significant interaction between Gravidity and Asset Index.
[2] The 95% confidence intervals were calculated using linearized standard errors
[3] These variables reflect practice at the time of the survey
Results are weighted for design effects and non-response

Table-S2: Sensitivity analysis - Unadjusted and adjusted[1] odds ratios (95% CI[2]) for stillbirth by pregnancy complications

Variable	Unadjusted Odds ratios (95%CI)	Main Model	Sensitivity analyses	
		Missing indicator method Adjusted Odds ratios (95%CI)	Complete case analysis[2] Adjusted Odds ratios (95%CI)	Multiple imputations[2] Adjusted Odds ratios (95%CI)
**Anemia**				
No	1 (Ref)	1 (Ref)	1 (Ref)	1 (Ref)
Yes	1·45 (1·36-1·54)	1·35 (1·27-1·43)	1·41 (1·30-1·52)	1·34 (1·26-1·42)
**Eclampsia**				
No	1 (Ref)	1 (Ref)	1 (Ref)	1 (Ref)
Yes	1·90 (1·73 -2·10)	1·79 (1·62 -1·97)	1·94 (1·72 -2·19)	1·79 (1·62 -1·98)
**Other hypertensive disorders**				
No	1 (Ref)	1 (Ref)	1 (Ref)	1 (Ref)
Yes	1·20 (1·05-1·36)	1·22 (1·07-1·38)	1·25 (1·07-1·46)	1·21 (1·06-1·38)
**Intra-partum Hemorrhage**				
No	1 (Ref)	1 (Ref)	1 (Ref)	1 (Ref)
Yes	2·97 (2·75 -3·21)	2·75 (2·54 -2·97)	2·73 (2·47 -3·02)	2·77 (2·56 -3·00)
**Ante-partum Hemorrhage**				
No	1 (Ref)	1 (Ref)	1 (Ref)	1 (Ref)
Yes	1·44 (1·25-1·67)	1·29 (1·11-1·50)	1·51 (1·39-1·64)	1·32 (1·14-1·53)
**Obstructed Labor**				
No	1 (Ref)	1 (Ref)	1 (Ref)	1 (Ref)
Yes	3·35 (3·13-3·58)	3·45 (3·19-3·74)	3·57 (3·24-3·94)	3·42 (3·16-3·70)
**Breech presentation**				
No	1 (Ref)	1 (Ref)	1 (Ref)	1 (Ref)
Yes	3·18 (2·88-3·50)	2·80 (2·51-3·12)	3·04 (2·65-3·47)	2·85 (2·56-3·18)
**Abnormal Fetal position**				
No	1 (Ref)	1 (Ref)	1 (Ref)	1 (Ref)
Yes	1·72 (1·54-1·92)	1·58 (1·40-1·77)	1·66 (1·43-1·91)	1·60 (1·43-1·80)

[1]Each multivariable logistic regression model adjusts for socioeconomic, health seeking behaviour, and bio-demographic characteristics identified to be significantly associated with Stillbirth in the previous model.
[2] Although the sub-population was restricted to women who had complete data on pregnancy-specific complication, the other variables adjusted for in the model had missing data, therefore sensitivity analyses were conducted.
Results are weighted for design effects and non-response

STROBE Statement—Checklist of items that should be included in reports of ***cross-sectional studies***

	Item No	Recommendation	Page No
**Title and abstract**	1	(*a*) Indicate the study's design with a commonly used term in the title or the abstract	1,2
		(*b*) Provide in the abstract an informative and balanced summary of what was done and what was found	2
**Introduction**			
Background/rationale	2	Explain the scientific background and rationale for the investigation being reported	4
Objectives	3	State specific objectives, including any prespecified hypotheses	4
**Methods**			
Study design	4	Present key elements of study design early in the paper	4, 5
Setting	5	Describe the setting, locations, and relevant dates, including periods of recruitment, exposure, follow-up, and data collection	4,5
Participants	6	(*a*) Give the eligibility criteria, and the sources and methods of selection of participants	4,5, Figure-1
Variables	7	Clearly define all outcomes, exposures, predictors, potential confounders, and effect modifiers. Give diagnostic criteria, if applicable	5,6,7
Data sources/ measurement	8*	For each variable of interest, give sources of data and details of methods of assessment (measurement). Describe comparability of assessment methods if there is more than one group	5,6
Bias	9	Describe any efforts to address potential sources of bias	6,7
Study size	10	Explain how the study size was arrived at	7
Quantitative variables	11	Explain how quantitative variables were handled in the analyses. If applicable, describe which groupings were chosen and why	5,6,7
Statistical methods	12	(*a*) Describe all statistical methods, including those used to control for confounding	6,7
		(*b*) Describe any methods used to examine subgroups and interactions	6,7
		(*c*) Explain how missing data were addressed	7
		(*d*) If applicable, describe analytical methods taking account of sampling strategy	7
		(*e*) Describe any sensitivity analyses	7
**Results**			
Participants	13*	(a) Report numbers of individuals at each stage of study—eg numbers potentially eligible, examined for eligibility, confirmed eligible, included in the study, completing follow-up, and analysed	4 and Figure-1
		(b) Give reasons for non-participation at each stage	Figure-1
		(c) Consider use of a flow diagram	Figure-1
Descriptive data	14*	(a) Give characteristics of study participants (eg demographic, clinical, social) and information on exposures and potential confounders	8,9, 10, 11, 12
		(b) Indicate number of participants with missing data for each	10,11,12

For peer review only

		variable of interest	
Outcome data	15*	Report numbers of outcome events or summary measures	8, 9
Main results	16	(*a*) Give unadjusted estimates and, if applicable, confounder-adjusted estimates and their precision (eg, 95% confidence interval). Make clear which confounders were adjusted for and why they were included	8, 9,10,11,12, 13,14, 15
		(*b*) Report category boundaries when continuous variables were categorized	9
		(*c*) If relevant, consider translating estimates of relative risk into absolute risk for a meaningful time period	Not applicable
Other analyses	17	Report other analyses done—eg analyses of subgroups and interactions, and sensitivity analyses	Table-S1 and S2
**Discussion**			
Key results	18	Summarise key results with reference to study objectives	16
Limitations	19	Discuss limitations of the study, taking into account sources of potential bias or imprecision. Discuss both direction and magnitude of any potential bias	18, 19
Interpretation	20	Give a cautious overall interpretation of results considering objectives, limitations, multiplicity of analyses, results from similar studies, and other relevant evidence	16,17,18
Generalisability	21	Discuss the generalisability (external validity) of the study results	17,18
**Other information**			
Funding	22	Give the source of funding and the role of the funders for the present study and, if applicable, for the original study on which the present article is based	22

*Give information separately for exposed and unexposed groups.

Note: An Explanation and Elaboration article discusses each checklist item and gives methodological background and published examples of transparent reporting. The STROBE checklist is best used in conjunction with this article (freely available on the Web sites of PLoS Medicine at http://www.plosmedicine.org/, Annals of Internal Medicine at http://www.annals.org/, and Epidemiology at http://www.epidem.com/). Information on the STROBE Initiative is available at www.strobe-statement.org.

For peer review only

For peer review only - http://bmjopen.bmj.com/site/about/guidelines.xhtml

BMJ Open

Stillbirth among women in nine states in India: Rate and risk factors in study of 886,505 women from the Annual Health Survey

Journal:	*BMJ Open*
Manuscript ID	bmjopen-2018-022583.R2
Article Type:	Research
Date Submitted by the Author:	12-Sep-2018
Complete List of Authors:	Altijani , Noon; University of Oxford, Nuffield Department of Population Health<br>Carson, Claire; University of Oxford, National Perinatal Epidemiology Unit (NPEU); National Perinatal Epidemiology Unit (NPEU),<br>Choudhury , Saswati; FAA Medical College and Hospital, Department of Obstetrics and Gynaecology<br>Rani, Anjali; Banaras Hindu University Institute of Medical Sciences, Department of Obstetrics and Gynaecology<br>Sarma, Umesh; Srimanta Sankaradeva University of Health Sciences<br>Knight, Marian; National Perinatal Epidemiology Unit<br>Nair, Manisha; University of Oxford, NPEU, Nuffield Department of Population Health
**Primary Subject Heading**:	Epidemiology
Secondary Subject Heading:	Global health, Public health
Keywords:	Stillbirth, Risk factors, Pregnancy complications, India

SCHOLARONE™
Manuscripts

TITLE PAGE

Stillbirth among women in nine states in India: Rate and risk factors in study of 886,505 women from the Annual Health Survey

Authors

1. Noon Altijani (MSc)
   Nuffield Department of Population Health, University of Oxford
   Richard Doll Building, Old Road Campus, Headington, Oxford, OX3 7LF, UK
   Email: noon.altijani@gtc.ox.ac.uk

2. Claire Carson (PhD)
   National Perinatal Epidemiology Unit, Nuffield Department of Population Health, University of Oxford
   Richard Doll Building, Old Road Campus, Headington, Oxford, OX3 7LF, UK
   Email: claire.carson@npeu.ox.ac.uk

3. Saswati Sanyal Choudhury (MD)
   Department of Obstetrics and Gynaecology, FAA Medical College and Hospital, Barpeta, Barpeta-Hospital-Jania Road, Joti Gaon, Barpeta, Assam 781301, India
   Email: saswatischoudhury@gmail.com

4. Anjali Rani (MD)
   Department of Obstetrics and Gynaecology, Institute of Medical Sciences, Banaras Hindu University, Ajagara, Banaras Hindu University Campus, Varanasi, Uttar Pradesh 221005, India
   Email: anjalirani.imsbhu@gmail.com

5. Umesh C Sarma (MD)
   Srimanta Sankaradeva University of Health Sciences, Assam
   Narkashur Hilltop, Christian Basti Bhangagarh, Guwahati, Assam 781032, India
   Email: ssuhs_assam@yahoo.in

6. Marian Knight (DPhil)
   National Perinatal Epidemiology Unit, Nuffield Department of Population Health, University of Oxford
   Richard Doll Building, Old Road Campus, Headington, Oxford, OX3 7LF, UK.
   Email: marian.knight@npeu.ox.ac.uk

7. Manisha Nair (DPhil)
   National Perinatal Epidemiology Unit, Nuffield Department of Population Health, University of Oxford
   Richard Doll Building, Old Road Campus, Headington, Oxford, OX3 7LF, UK
   Email: manisha.nair@npeu.ox.ac.uk

Corresponding author

Manisha Nair
Senior Epidemiologist and Research Fellow,
National Perinatal Epidemiology Unit, NDPH, University of Oxford
Richard Doll Building, Old Road Campus, Headington, Oxford, OX3 7LF, UK
Email: manisha.nair@npeu.ox.ac.uk; Phone: +44-(0)1865-617820

For peer review only - http://bmjopen.bmj.com/site/about/guidelines.xhtml

ABSTRACT

Objectives: To assess the rate of stillbirth and associated risk factors across nine states in India.

Design: Secondary analysis of cross-sectional data from the Indian Annual Health Survey (2010-2013).

Setting: Nine states in India: Madhya Pradesh, Chattisgarh, Rajasthan, Uttarakhand, Jharkhand, Odisha, Bihar, Assam, Uttar Pradesh

Participants: 886,505 women, aged 15–49 years

Main outcome measures: Stillbirth rate with 95% Confidence Interval (CI). Adjusted odds ratio to examine the associations between stillbirth and (i) socioeconomic, behavioural, and bio-demographic risk factors (ii) complications in pregnancy (anaemia, eclampsia, other hypertensive disorders, antepartum and intrapartum haemorrhage, obstructed labour, breech presentation, abnormal fetal position).

Results: The overall rate of stillbirth was 10 per 1000 total births (95%CI: 9·8-10·3). Indicators of socioeconomic deprivation were strongly associated with an increase in stillbirth: rural residence (aOR:1·27,95%CI:1·16-1·39), female illiteracy (aOR:1·43,95%CI 1·17-1·74); low socioeconomic status (aOR:2·42, 95%CI:1·82-3·21); schedule caste background (aOR:1·11,95%CI:1·04-1·19), and woman not in paid employment (aOR:1·15,95%CI:1·07-1·24). Women from minority religious groups were at higher risk than the Hindu majority (Muslim (aOR:1·33,95%CI:1·25-1·43); Christian (aOR:1·42,95%CI:1·19-1·70)). While few women smoked (<1%), around 9% reported chewing tobacco, which was associated with an increased odds of stillbirth (aOR:1·11,95%CI:1·02-1·21). Adverse pregnancy and birth characteristics were also associated with stillbirth: antenatal care visits <4 (aOR:1·08,95%CI:1·01-1·15), maternal age <25 years (aOR:1·29,95%CI:1·21-1·37) and ≥35 years (aOR:1·16,95%CI:1·04-1·29), multigravida (aOR:3·06,95%CI:2·42-3·86), multiple pregnancy (aOR:1·77,95%CI:1·47-2·15), assisted delivery (aOR:3·45,95%CI:3·02-3·93), caesarean section (aOR:1·73,95%CI:1·58-1·89), as were pregnancy complications (aOR 1.42, 95%CI:1.33 – 1.51).

Conclusion: India is an emerging market economy experiencing a rapid health transition, yet these findings demonstrate the marked disparity in risk of stillbirth by women's socioeconomic status. Tobacco chewing, maternal and fetal complications were each found to be important modifiable risk factors. Targeting the 'at-risk' population identified here, improved recording of stillbirths and the introduction of local reviews would be important steps to reduce the high burden of stillbirths in India.

ARTICLE SUMMARY

Strengths and limitations of this study

- India has the highest number of stillbirths, globally. This study identifies the characteristics of high risk women and key risk factors in the Indian context using the largest available dataset

For peer review only - http://bmjopen.bmj.com/site/about/guidelines.xhtml

- The data were drawn from Indian Annual Health Survey (2010-2013), which sampled women from the nine states that account for 50% of the country’s population, using methods designed to minimise selection bias.
- This study analyses a large sample (>800,000 women), providing sufficient statistical power to conduct a robust examination of a wide range of risk factors.
- The findings are generalizable for high burden states included in this study, but may not be generalizable to the rest of India.
- However, retrospective data collection can lead to recall bias, and there is the potential for some under-reporting of stillbirth.

For peer review only

Key words: Stillbirth, Risk factors, Pregnancy complications, India

Word count: 3650

For peer review only - http://bmjopen.bmj.com/site/about/guidelines.xhtml

INTRODUCTION

Stillbirth is an important global health problem affecting over 7000 families every day and is associated with emotional, social and economic consequences.[1] In 2015 the stillbirth rate (SBR) was 18.4 per 1000 total births worldwide.[1] The progress in reducing stillbirth since 1990 has been slower than reductions in neonatal and under-five child mortality.[2] Currently, 98% of stillbirths occur in low-to-middle-income countries (LMICs)[1] and India has the highest number of stillbirths, with an estimated 592,100 deaths per year[3], and a World Health Organisation (WHO) estimated rate of 22 per 1000 total births. The Government of India has developed an Indian Newborn Action Plan (INAP) which includes efforts to reduce stillbirths to <10 per 1,000 births by 2030".[4] A modest reduction in India's SBR would translate into thousands of lives saved.

While previous studies have examined the immediate pregnancy-related risk factors for stillbirth such as infections during pregnancy and hypertensive disorders,[1 5] knowledge about distal risk factors such as socioeconomic, lifestyle related and co-morbidities is limited.[1] The Indian government recognises the need to improve pregnancy care and institutional delivery among disadvantaged socioeconomic groups who have a higher risk of maternal and fetal death.[6] Since 2005, the government has made several efforts including cash assistance and dedicated services through community health workers with a stronger focus in the states with poor health and development indicators.[6] The objective of this study was to understand the current disparities in the risk of stillbirth in these 'high focus' states by examining the association between socioeconomic, bio-demographic, behavioural, and pregnancy-specific risk factors and stillbirth in nine states in India.

METHODS

We conducted an analysis of secondary data from India's Annual Health Survey (2010-13). The survey covers nine states (Madhya Pradesh, Chattisgarh, Rajasthan, Uttarakhand, Jharkhand, Odisha, Bihar, Assam, Uttar Pradesh) which account for about 50% of the country's population.[7] A total of 886,505 women for whom information about the outcome of their last pregnancy (live birth or stillbirth) was available and whose pregnancy lasted more than seven completed months (~28 weeks' gestation) were included. Figure-1 illustrates how the sample for this study was derived.

Based on the outcome of last pregnancy, women were divided into two groups, those with a livebirth and those with a stillbirth. The outcome data is from the Women schedule (Section-

For peer review only - http://bmjopen.bmj.com/site/about/guidelines.xhtml

1) that was implemented during the baseline round of the Annual Health Survey (AHS) in 2010-11. Ever married women in the age group 15-49 years were asked about the outcome of their last pregnancy during the reference period 01-January-2007 to 31-December-2009, which was reported as either livebirth, stillbirth or abortion. Information on gestational age at stillbirth or type of stillbirth (antepartum or intrapartum) was not available. The numerator 'stillbirth' and denominator 'total birth = stillbirth + livebirth) is from the same reference period of the survey data. Potential risk factors for stillbirth were grouped as: socioeconomic, behavioural, and bio-demographic based on a review of published literature. The description of variables included in each group is provided in Table-1. A directed acyclic graph was used to construct a theoretical framework of relationships between the aforementioned risk factors and stillbirth (Figure-2). In addition, in a sub-sample of the population (n=668,892), we examined the association of stillbirth with the following self-reported problems/ complications during pregnancy: anaemia, eclampsia, other hypertensive disorders, antepartum haemorrhage, intrapartum haemorrhage, abnormal fetal position, breech presentation, and obstructed labour.

Table 1: Description of the variables used to examine the risk factors for stillbirth

Risk factors	Description of the variables
**Proximal or Bio-Demographic factors**	
**Fetal factors**	
Sex	Sex of the foetus was coded as either female or male
Multiple pregnancy	Number of foetuses: Women were categorised as singleton (one foetus) or multiple pregnancy (twins or higher-order multiple gestations)
**Maternal factors**	
Maternal age	Maternal age at the time of the survey was divided into five year age bands: <20, 20-24, 25-29, 30-34, 35-39, 40-44, 45 and above
Gravidity	Gravidity (number of pregnancies) was used as a proxy for parity (number of deliveries) for which information was not available· Gravidity was categorised as "first" if the index pregnancy was the first pregnancy, "second - fourth", and "five or more"·
Any complication during pregnancy	Women who reported to have any of the following complications during their index pregnancy we coded as 'yes', otherwise 'no': anaemia, eclampsia, other hypertensive disorders, antepartum haemorrhage, intrapartum haemorrhage, abnormal fetal position, breech presentation, and obstructed labour
Mode of delivery	This was categorised into vaginal delivery, assisted vaginal delivery and Caesarean section
**Intermediate factors or behavioural factors**	
**Lifestyle factors**	
Smoking tobacco	Women were asked about smoking and chewing tobacco during the interview; current practice was coded as "yes", while women who never practiced or no longer practiced were coded as "no"
Chewing tobacco	
**Health seeking behaviour**	
Antenatal care visits	Number of antenatal care (ANC) visits was categorised as '≥4 visits'

For peer review only - http://bmjopen.bmj.com/site/about/guidelines.xhtml

	(which is recommended as adequate by the WHO), and '<4 visits'
Timing of first ANC visit	Timing of the first ANC visit was categorised as 'visit in the first trimester (or first three months), which is recommended as adequate by the WHO, and 'after first three months'
Place of delivery	Place of delivery was grouped as "medical facility" or "home"
**Distal or Socioeconomic factors**	
**Social factors**	
Religion	Women were categorised into the following religious groups: 'Hindu', 'Muslim', 'Christian', and 'Others' which included several groups with small numbers such as Sikh, Buddhists, etc
Place of Residence	Place of residence was grouped into urban and rural
Social group	Women were categorised into the following social groups: 'Schedule caste (SC)', 'Schedule tribe (ST)' and 'Others'<br>○ SC and ST are officially designated groups of historically disadvantaged populations in India<br>○ 'Others' included the general social class and other backward classes
**Economic factors**	
Education	Maternal education at the time of the survey was categorised into: illiterate, primary school or below, secondary school, tertiary and above
Occupation	Based on occupational status, women were grouped as 'being in paid employment' or 'not in paid employment'
Economic status	Asset index was used to measure the economic status of the participants· Asset index scores were calculated and the study sample was divided into quintiles ranging from the lowest (quintile-1) to highest (quintile-5) socioeconomic status
**Pregnancy complication**	
Anaemia during pregnancy	This is a derived variable based on questions related to the following signs and symptoms: paleness, giddiness, weakness, excessive fatigue

Statistical analysis

Overall and state-specific rates of stillbirth per 1000 total births with 95% confidence intervals (CIs) were calculated in the study population. The characteristics of women who had a stillbirth were compared with those who had a livebirth. A univariable logistic regression analysis was performed to investigate the association between each potential risk factor and stillbirth. Maternal age was tested for linearity which showed the presence of a non-linear association with stillbirth (Figure-3); it was therefore used as a categorical variable. A multivariable model was built using a step-wise forward regression, including risk factors that were statistically significantly associated with stillbirth during univariable analysis (based on a Wald p-value of <0.05). The order in which the variables were added to the model, distal followed by intermediate then proximal factors, was informed by the theoretical framework (Figure-2). Three variables, 'smoking tobacco', 'place of delivery' and 'timing of

For peer review only - http://bmjopen.bmj.com/site/about/guidelines.xhtml

first ANC visit' were not statistically significantly associated with stillbirth at $p<0.05$ during the model building process, and were therefore dropped from the final models.

Calculated pairwise correlation coefficients did not show any significant collinearity between the risk factors. The following interactions were identified a priori and tested for significance using Wald test, which is a method of choice for survey-weighted data: Asset index and gravidity, place of residence and place of delivery. We found a significant interaction between 'Gravidity' (number of pregnancies) and 'Asset index'. The final regression model was adjusted for this by fitting an interaction term. We conducted further analysis to examine the association between gravidity stratified by the quintiles of the Asset index.

In a sub-sample of the study population with complete data on complications during pregnancy and medical co-morbidities, we conducted univariable logistic regression analysis to investigate their individual association with stillbirth. Statistically significant associations were further examined using multivariable logistic regression models that adjusted for socioeconomic, behavioural and bio-demographic risk factors found to be significantly associated with stillbirth. We compared the proportion of stillbirth in the subsample with the excluded group and the total sample which showed that in all three groups the proportion of stillbirth was about 1% and livebirth 99%. This suggests that the subsample for the specific pregnancy complication analysis was not a biased sample.

Missingness was investigated and data were assumed to be 'Missing at Random'. Three methods were used to address bias due to missing data; missing indicator method, complete case analysis, and multiple imputations.[8] The 'missing indicator' model in which missing data were grouped as a separate category was used as the final model. However, to maintain model stability, for variables that had <1% missing data, a separate category for 'missing' was not generated. All analyses accounted for the stratified, clustered nature of the data and were conducted using the survey command (svy) in Stata V.13.1, SE (StataCorp, College Station, Texas, USA). The proportions, means and confidence intervals presented here are weighted for design effects and non-response.

Study power

The fixed sample had more than 90% power to detect an OR of ≥1.11 or ≤0.91 associated with stillbirth at $p<0.05$ (two-tailed) for the risk factor with the highest prevalence (83% for rural residence) and an OR of ≥1.39 or ≤0.67 for the risk factor with the lowest prevalence in the study population (current smoker 0.8%).

Patient and Public Involvement

For peer review only - http://bmjopen.bmj.com/site/about/guidelines.xhtml

This is not applicable since this was a secondary analysis of anonymous survey data.

RESULTS

Rate of Stillbirth

Of the 886,505 women included in the analyses 8,429 reported a stillbirth, giving an overall rate of 10 stillbirths (95%CI 9.8-10.3) per 1000 total births. The rates of stillbirth per state are shown in Table-2 and Figure-4.

Table 2: Number and rate of stillbirths in the study population in nine states in India

State	Stillbirths	Total births	Stillbirth rate per 1000 total birth (95% CI)
Assam	856	64 841	12·8 (11·8-13·9)
Bihar	2 833	250 609	11·3 (10·9-11·8)
Chhattisgarh	476	98 220	5·0 (4·5-5·5)
Jharkhand	657	60 153	10·6 (9·7-11·6)
Madhya Pradesh	615	145 552	4·2 (3·8 - 4·6)
Odisha	534	46 162	10·9 (9·9-12·0)
Rajasthan	413	52 537	7·1 (6·4-8·0)
Uttar Pradesh	1 813	131 324	14·8 (14·1-15·6)
Uttarakhand	233	37 485	7·7 (6·5-9·2)
Overall	8 430	886 505	10·0 (9·8-10·3)

Frequencies are unweighted (true counts). Rates are weighted for design effects and non-response

Association between socioeconomic, behavioural, and bio-demographic characteristics and stillbirth

The median age for women in the study population was 26 years (Interquartile range 23-30). The frequency, proportions and association with stillbirth (unadjusted and adjusted OR) for socioeconomic, behavioural and bio-demographic variables are shown in Table 3, and pregnancy-related variables in Table 4. After adjusting for other risk factors, all examined socioeconomic factors were found to be significantly associated with stillbirth. Women living in rural settings had 27% higher odds of stillbirth compared with urban settings. Belonging to a Schedule caste social group was associated with 11% higher odds compared with the reference 'other' social group, but 26% lower odds of stillbirth for women belonging to the Schedule tribe group. Compared with Hindus, belonging to Muslim and Christian religious groups was associated with increased odds of stillbirth. Compared with women with university education or higher, women with no school education had 43% higher odds. Women not in paid employment were 15% more likely to have a stillborn baby compared with women in paid employment.

Chewing tobacco was associated with 11% higher odds of stillbirth after adjusting for other risk factors in the study population. Health seeking behaviours were significantly associated

For peer review only - http://bmjopen.bmj.com/site/about/guidelines.xhtml

For peer review only

with stillbirth. Women attending <4 antenatal care (ANC) visits had 8% higher odds compared with women who had ≥4 ANC visits.

All examined demographic and pregnancy-related factors were also found to be significantly associated with stillbirth in the study population. After full adjustment, the non-linear (U-shaped) association between mother's age and stillbirth persisted. Multiple pregnancies were associated with 77% higher odds of stillbirth compared with singleton. Pregnancies with a male fetus had 26% higher odds compared with a female fetus. Women who had an assisted delivery or caesarean section were more likely to report a stillbirth compared with women who had a normal delivery.

We found a significant statistical interaction between 'gravidity' and 'Asset index'. A stratified analysis showed that multigravida women had higher odds of stillbirth and this association was substantially stronger for women who belonged to the higher Asset Index quintiles (Table-5). The results of sensitivity analyses (complete case analysis and multiple imputations) were not materially different from the 'missing indicator' model (Supplementary Table-S1).

For peer review only

For peer review only - http://bmjopen.bmj.com/site/about/guidelines.xhtml

Table 3: The Association between stillbirth and socioeconomic behaviouraland bio-demographic factors: frequencies, unadjusted and adjusted Odds Ratios

Variables	Total births N=886 505 Frequency (%)	Stillbirths N= 8 429 Frequency (%)	Live births, N= 878 076 Frequency (%)	Unadjusted Odds Ratios (95%CI[2])	Adjusted Odds Ratios[1] (95%CI[2])
**Maternal Age (years)**					
15-19	33 482 (3·7)	436 (4·9)	33 046 (3·7)	1·52 (1·35-1·72)	1·76 (1·55-2·00)
20-24	324 130 (36·0)	3 079 (36·2)	321 051 (36·0)	1·17 (1·09-1·24)	1·29 (1·21-1·37)
25-29	301 330 (33·7)	2 546 (29·2)	298 784 (33·7)	1·00 (Ref)	1·00 (Ref)
30-34	143 529 (16·6)	1 400 (17·1)	142 129 (16·6)	1·20 (1·11-1·29)	1·05 (0·97-1·14)
35-39	55 893 (6·6)	643 (8·2)	55 250 (6·6)	1·43 (1·29-1·59)	1·16 (1·04-1·29)
40-45	20 006 (2·4)	244 (3·4)	19 762 (2·4)	1·63 (1·39-1·91)	1·29 (1·09-1·51)
45-49	8 135 (1·0)	81 (1·1)	8 054 (1·0)	1·24 (0·96-1·60)	1·04 (0·80-1·35)
**Place of Residence**					
Urban	123 099 (16·4)	874 (12·7)	122 225 (16·4)	1·00 (Ref)	1·00 (Ref)
Rural	763 406 (83·7)	7 555 (87·4)	755 851 (83·6)	1·35 (1·24-1·47)	1·27 (1·16 - 1·39)
**Religion**					
Hindu	734 134 (81·5)	6 522 (75·3)	727 612 (81·5)	1·00 (Ref)	1·00 (Ref)
Muslim	125 723 (15·9)	1 619 (21·6)	124 104 (15·8)	1·48 (1·39-1·58)	1·33 (1·25-1·43)
Christian	14 914 (1·8)	178 (2·4)	14 736 (1·8)	1·49 (1·25-1·78)	1·42 (1·19-1·70)
Others	11 734 (0·9)	110 (0·7)	11 624 (0·9)	0·87 (0·69-1·11)	1·06 (0·84-1·35)
**Social Group**					
Other	598 477 (68·4)	5 868 (70·0)	592 609 (68·4)	1·00 (Ref)	1·00 (Ref)
Schedule Caste	161 273 (19·2)	1 644 (21·4)	159 629 (19·2)	1·09 (1·02-1·16)	1·11 (1·04-1·19)
Schedule Tribe	126 755 (12·4)	917 (8·7)	125 838 (12·5)	0·68 (0·62-0·74)	0·74 (0·68-0·81)
**Maternal Education**					
Tertiary and above	36 617 (3·9)	188 (2·2)	36 429 (4·0)	1·00 (Ref)	1·00 (Ref)
Secondary	110 458 (11·3)	786 (8·2)	109 672 (11·3)	1·31 (1·08-1·60)	1·10 (0·90-1·34)
Primary and below	364 809 (39·1)	3 413 (38·1)	361 396 (39·1)	1·77 (1·48-2·12)	1·34 (1·10-1·62)
Illiterate	374 621 (45·7)	4 042 (51·6)	370 579 (45·6)	2·06 (1·72-2·47)	1·43 (1·17-1·74)
**Employment**					
In paid employment	154 177 (16·4)	1 273 (13·2)	152 904 (16·4)	1·00 (Ref)	1·00 (Ref)
Not in-paid employment	732 328 (83·6)	7 156 (86·8)	725 172 (83·6)	1·29 (1·20-1·38)	1·15 (1·07-1·24)
**Asset Index, Quintiles**					
5 "Highest"	159 957 (17·9)	1 133 (13·6)	158 824 (17·9)	1·00 (Ref)	1·00 (Ref)
4	160 335 (17·6)	1 422 (16·9)	158 913 (17·6)	1·26 (1·14-1·38)	1·34 (1·01-1·79)
3	159 567 (18·0)	1 606 (19·3)	157 961 (18·0)	1·41 (1·29-1·55)	1·91 (1·45-2·52)
2	157 506 (17·9)	1 654 (19·8)	155 852 (17·8)	1·46 (1·33-1·60)	2·45 (1·85-3·24)
1 "Lowest"	155 283 (18·0)	1 626 (18·9)	153 657 (17·9)	1·39 (1·26-1·52)	2·42 (1·82-3·21)
Missing	93 857 (10·7)	988 (11·5)	92 869 (10·7)	1·41 (1·27-1·56)	1·21 (0·91-1·62)
**Current Smoking[3]**					
No	725 560 (82·0)	6 670 (79·6)	718 890 (82·0)	1·00 (Ref)	-
Yes	6 553 (0·8)	80 (1·2)	6 473 (0·8)	1·53 (1·19-1·97)	-
Missing	154 392 (17·2)	1 679(19·3)	152 713 (17·2)	1·16 (1·08-1·23)	-

For peer review only - http://bmjopen.bmj.com/site/about/guidelines.xhtml

**Chewing Tobacco**[3]					
No	635 393 (74·1)	5 743 (71·5)	629 650 (74·1)	1·00 (Ref)	1·00 (Ref)
Yes	96 806 (8·7)	1 010 (9·3)	95 796 (8·7)	1·10 (1·01-1·19)	1·11 (1·02-1·21)
Missing	154 306 (17·2)	1 676 (19·2)	152 630 (17·2)	1·16 (1·09-1·24)	1·44 (1·29-1·59)

[1] Multivariable logistic regression, adjusting for the other variables in the model –except self-reported mental illness. The model also adjusts for the observed significant interaction between Gravidity and Asset Index. Smoking was not significantly associated with stillbirth during the model building process and therefore, removed from the final model.

[2] The 95% confidence intervals were calculated using linearized standard errors

[3] These variables reflect practice at the time of the survey

Frequencies are true counts, % and OR are weighted for design effects and non-response

For peer review only

Table 4: The Association between stillbirth and pregnancy-related factors: frequencies, unadjusted and adjusted Odds Ratios

Variables	Total births N=886 505 Frequency (%)	Stillbirths N= 8 429 Frequency (%)	Live births N= 878 076 Frequency (%)	Unadjusted Odds Ratios (95%CI[2])	Adjusted Odds Ratios[1] (95%CI[2])
**Number of antenatal care visit**					
4 or more	269 148 (28·9)	2 222 (24·6)	266 926 (29·0)	1·00 (Ref)	1·00 (Ref)
Less than 4	518 288 (58·7)	4 939 (59·3)	513 349 (58·6)	1·19 (1·12-1·26)	1·08 (1·01-1·15)
Missing	99 069 (12·4)	1 268 (16·1)	97 801 (12·4)	1·53 (1·41-1·66)	1·36 (1·25-1·48)
**Timing of the first antenatal care visit**					
Three or less	534 118 (58·6)	4 678 (54·0)	529 440 (58·7)	1·00 (Ref)	-
More than three	253 460 (28·9)	2 483 (29·9)	250 977 (28·9)	1·12 (1·06-1·19)	-
Missing	98 927 (12·4)	1 268 (16·1)	97 659 (12·4)	1·41 (1·31-1·52)	-
**Place of delivery**					
Medical facility	539 704 (60·5)	5 176 (61·9)	534 528 (60·5)	1·00 (Ref)	-
Home	346 801 (39·5)	3 253 (38·1)	343 548 (39·5)	0·94 (0·89-0·99)	-
**Number of pregnancies**					
1	158 886 (16·9)	957 (10·6)	157 929 (16·9)	1·00 (Ref)	1·00 (Ref)
2-4	414 938 (46·8)	4 409 (51·8)	410 529 (46·7)	1·77 (1·63-1·93)	3·06 (2·42-3·86)
5 or more	93 458 (11·7)	1 404 (19·0)	92 024 (11·7)	2·60 (2·36-2·87)	4·98 (3·66-6·74)
Missing	219 253 (24·7)	1 659 (18·6)	217 594 (24·7)	1·21 (1·10-1·32)	1·40 (0·68-2·90)
**Sex of the baby**					
Female	414 913 (46·6)	3 468 (41·2)	411 445 (46·7)	1·00 (Ref)	1·00 (Ref)
Male	471 592 (53·4)	4 961 (58·9)	466 631 (53·3)	1·25 (1·19-1·32)	1·26 (1·20 - 1·33)
**Multiple gestations (index pregnancy)**					
Singleton	878 556 (99·1)	8 267 (98·2)	870 289 (99·1)	1·00 (Ref)	1·00 (Ref)
Twin pregnancy	7 949 (0·9)	162 (1·8)	7 787 (0·9)	2·03 (1·67-2·45)	1·77 (1·47 - 2·15)
**Mode of delivery**					
Spontaneous vaginal	805 430 (90·7)	7 146 (84·3)	798 284 (90·8)	1·00 (Ref)	1·00 (Ref)
Assisted vaginal	14 909 (1·8)	390 (5·0)	14 519 (1·7)	3·13 (2·76-3·55)	3·45 (3·02-3·93)
Caesarean section	66 166 (7·6)	893 (10·7)	65 273 (7·5)	1·53 (1·40-1·66)	1·73 (1·58-1·89)
**Any pregnancy complications**					
No	250 099 (28·1)	2 000 (23·5)	250 099 (28·1)	1·00 (Ref)	1·00 (Ref)
Yes	418 856 (47·5)	4 779 (58·0)	414 077 (47·4)	1·47 (1·38-1·56)	1·42 (1·33-1·51)
Missing	217 550 (24·5)	1 650 (18·5)	215 900 (24·5)	0·91 (0·84-0·98)	1·99 (1·00-3·94)

[1] Multivariable logistic regression, adjusting for the other variables in the model –except self-reported mental illness. The model also adjusts for the observed significant interaction between Gravidity and Asset Index.

[2] The 95% confidence intervals were calculated using linearized standard errors

Frequencies are true counts, % and OR are weighted for design effects and non-response

For peer review only - http://bmjopen.bmj.com/site/about/guidelines.xhtml

Table 5: Adjusted[1] odds ratios and 95%CI for the association between gravidity and stillbirth stratified by the Asset index quintiles

**A· Missing Indicator Method**					
	**Asset Index Quintiles**				
	**1 (most deprived)**	**2**	**3**	**4**	**5 (least deprived)**
Gravidity					
1	1·00 (Ref)	1·00 (Ref)	1·00 (Ref)	1·00 (Ref)	1·00 (Ref)
2-4	1·44 (1·15-1·81)	1·47 (1·18-1·84)	1·83 (1·48 - 2·26)	2·55 (2·02 – 3·23)	3·04 (2·37 – 3·90)
More than 5	1·89 (1·43-2·49)	1·88 (1·43-2·48)	3·04 (2·34 - 3·94)	3·71 (2·75 - 5·02)	4·62 (3·27 - 6·54)
Missing	0·95 (0·74-1·22)	1·18 (0·92-1·50)	1·37 (1·09-1·72)	1·88 (1·44 – 2·45)	2·26 (1·67 – 3·05)
**B· Complete Case Analysis**					
	**Asset Index Quintiles**				
	**1**	**2**	**3**	**4**	**5**
Gravidity					
1	1·00 (Ref)	1·00 (Ref)	1·00 (Ref)	1·00 (Ref)	1·00 (Ref)
2-4	1·19 (0·92-1·54)	1·22 (0·94-1·60)	1·74 (1·35 - 2·24)	2·05 (1·56 – 2·67)	2·86 (2·11 – 3·88)
More than 5	1·59 (1·15-2·18)	1·48 (1·05-2·08)	2·70 (1·97 - 3·73)	3·02 (2·12 - 4·31)	5·02 (3·35 - 7·53)
**C· Multiple Imputations**					
	**Asset Index Quintiles**				
	**1**	**2**	**3**	**4**	**5**
Gravidity					
1	1·00 (Ref)	1·00 (Ref)	1·00 (Ref)	1·00 (Ref)	1·00 (Ref)
2-4	1·48 (0·90-2·48)	1·50 (0·90-2·55)	1·77 (1·08-2·92)	2·29 (1·39-3·84)	2·73 (2·20 – 3·40)
More than 5	1·90 (1·00-3·52)	1·98 (1·07-3·81)	2·69 (1·40-5·04)	3·31 (1·70-6·45)	4·41 (3·33 - 5·86)

[1]Multivariable logistic regression, adjusting for socioeconomic, health seeking behaviour, pre-existing medical conditions, and bio-demographic characteristics
Results are weighted for design effects and non-response

For peer review only - http://bmjopen.bmj.com/site/about/guidelines.xhtml

Association between specific complications during pregnancy and stillbirth

Reporting any pregnancy complication was associated with significantly higher odds of stillbirth after adjusting for the identified socioeconomic, behavioural, and bio-demographic risk factors (Table-4). The association between each of the reported complication and stillbirth are shown in Table-6. Women who reported to have anaemia during pregnancy had 35% higher odds of stillbirth compared with women who did not have anaemia. Eclampsia was associated with almost twice the odds of stillbirth and other hypertensive disorders were associated with about 22% higher odds. Women who had an ante-partum or intra-partum haemorrhage had significantly higher odds of stillbirth compared with women who did not have these complications. The adjusted odds of stillbirth were 58% higher in women with abnormal fetal position, more than three times higher for women who had an obstructed labour, and nearly three times higher for women who had a breech presentation compared with women who did not report these complications. We also noted that the association between stillbirth and caesarean section was no longer significant at $p<0.05$ and the aOR for assisted delivery was significantly attenuated in the model that examined the association between obstructed labour and stillbirth. Sensitivity analysis conducted using complete case analysis and multiple imputations did not change the results materially (Supplementary Table-S2).

For peer review only - http://bmjopen.bmj.com/site/about/guidelines.xhtml

Table 6: Association between stillbirth and complications during pregnancy–frequencies, unadjusted and adjusted Odds Ratios (95% CI)

Variables	Total births N=668,892 Frequency (%)	Stillbirths N= 6,777 Frequency (%)	Live births, N= 662,115 Frequency (%)	Unadjusted Odds ratios (95%CI)	Adjusted Odds ratios (95%CI)
**Anemia**					
No	500 785 (73.8)	4 611 (66.0)	496 174 (73.9)	1 (Ref)	1 (Ref)
Yes	168 107 (26.2)	2 166 (34.0)	165 941 (26.1)	1·45 (1·36-1·54)	1·35 (1·27-1·43)
**Eclampsia**					
No	628 681 (93.8)	6 043 (88.9)	622 638 (93.8)	1 (Ref)	1 (Ref)
Yes	40 211 (6.2)	734 (11.2)	39 477 (6.2)	1·90 (1·73 -2·10)	1·79 (1·62 -1·97)
**Other hypertensive disorders**					
No	639 689 (95.5)	6 412 (94.7)	633 277 (95.5)	1 (Ref)	1 (Ref)
Yes	29 203 (4.5)	365 (5.3)	28 838 (4.5)	1·20 (1·05-1·36)	1·22 (1·07-1·38)
**Intra-partum Hemorrhage**					
No	622 206 (93.3)	5 559 (82.6)	616 647 (93.4)	1 (Ref)	1 (Ref)
Yes	46 686 (6.7)	1 218 (17.4)	45 468 (6.6)	2·97 (2·75 -3·21)	2·75 (2·54 -2·97)
**Ante-partum Hemorrhage**					
No	650 631 (97.2)	6 488 (96.0)	644 143 (97.2)	1 (Ref)	1 (Ref)
Yes	18 261 (2.8)	289 (4.0)	17 972 (2.8)	1·44 (1·25-1·67)	1·29 (1·11-1·50)
**Obstructed Labor**					
No	602 184 (90.1)	5 037 (73.4)	597 147 (90.2)	1 (Ref)	1 (Ref)
Yes	66 708 (9.9)	1 740 (26.6)	64 968 (9.8)	3·35 (3·13-3·58)	3·45 (3·19-3·74)
**Breech presentation**					
No	645 162 (96.2)	6 092 (89.1)	639 070 (96.3)	1 (Ref)	1 (Ref)
Yes	23 730 (3.8)	685 (10.9)	23 045 (3.7)	3·18 (2·88-3·50)	2·80 (2·51-3·12)
**Abnormal Fetal position**					
No	640 015 (95.4)	6 269 (92.5)	633 746 (95.5)	1 (Ref)	1 (Ref)
Yes	28 877 (4.6)	508 (7.5)	28 369 (4.5)	1·72 (1·54-1·92)	1·58 (1·40-1·77)

[1]Each multivariable logistic regression model adjusts for socioeconomic, health seeking behaviour, and bio-demographic characteristics identified to be significantly associated with Stillbirth in the previous model.
[2] Although the sub-population was restricted to women who had complete data on pregnancy-specific complication, the other variables adjusted for in the model had missing data, therefore sensitivity analyses were conducted.
Frequencies are true counts, % and OR are weighted for design effects and non-response

For peer review only - http://bmjopen.bmj.com/site/about/guidelines.xhtml

DISCUSSION

The overall rate of stillbirth was found to be 10 per 1000 total births, but varied widely between the nine states ranging from 4.2 to 14.8 per 1000 total births. The marked variation at the state level may be explained by the different population characteristics and the varying distribution of risk factors across the nine states. The findings show the impact of inequality on stillbirth, as women in the most deprived groups are at highest risk. This was identified across a number of variables which capture different aspects of deprivation; for example, poorer women, those with little education, those living in rural areas, not currently working and belonging to 'schedule caste' groups are at increased risk of stillbirth compared to more affluent and advantaged women. Yet, among the multigravidas odds of stillbirth were substantially higher in the more affluent group, after adjusting for other factors. Chewing tobacco is highlighted as a significant risk factor for stillbirth, which is important in the Indian context where few women report tobacco smoking. Pregnancy complications, including anaemia, eclampsia, other hypertensive disorders, antepartum haemorrhage, intrapartum haemorrhage, abnormal fetal position, breech presentation, and obstructed labour significantly increased the odds of stillbirth.

The overall estimated stillbirth rate in the study population from the nine states in India was approximately half that of the World Health Organisation (WHO) estimated rate of 22 per 1000 total births. This was also true for the rate of stillbirth for the state of Bihar (11.3 per 1000 births; 95% CI = 10.9-11.8 reported in Table 2) when compared with the rate estimated for this state by another study that used both household survey and verbal autopsy to estimate the rate (21.2 per 1,000 births; 95% CI 19.7 to 22.6).[9] However, the risk factors for stillbirth identified by our study is consistent with the findings of the other study by Dandona et al.[9] Possible explanations for the low stillbirth rate include under-reporting of stillbirths or classifying stillbirths as neonatal deaths due to associated stigma. It is also important to acknowledge that the rates of stillbirth from the WHO, from Dandona et al's study[9] and from our study are all estimates, and it is difficult to confirm which estimate is the most accurate.

We found a significant disparity in the risk of stillbirth by socioeconomic status which is consistent with previous research. A systematic review of studies from developing countries showed that low socioeconomic status was significantly associated with stillbirth with a population attributable fraction ranging between 2% and 75%.[10] A study from 13 European countries reported that lower maternal education and maternal unemployment were associated with 1.9 and 1.6 times higher odds of stillbirth, respectively.[10] Maternal

For peer review only - http://bmjopen.bmj.com/site/about/guidelines.xhtml

education and employment may act through promoting high self-esteem and empowering women to make decisions about healthcare utilisation.[11]

The increased odds of stillbirth in women belonging to religious minority groups in India could be due to cultural constraints reported in other studies or inequalities that minority groups are subjected to which influences their healthcare seeking behaviours, even in countries that have universal access to medical care.[12] In India, Schedule tribe and Schedule caste social groups have been historically disadvantaged, belonging to a Schedule caste group was associated with higher odds of stillbirth which is consistent with previous literature of disadvantaged groups having higher risk.[13] However, women belonging to the Schedule tribe group had lower odds of stillbirth. While, it might be possible that individuals belonging to Schedule tribe groups have different health seeking behaviours leading to a lower risk,[14 15] other factors, not adjusted for directly in this study, could explain this difference. Examples include nutritional risk factors, exposure to environmental toxin through occupation or household (such as indoor air pollution) or exposure to domestic violence,

Self-reported tobacco smoking or chewing has been shown to be associated with increased risk of stillbirth in other studies.[16 17] In the study population, chewing tobacco was 15-times more common compared with smoking tobacco and women who chewed tobacco had a 14% higher odds of stillbirth compared with women who did not. In contrast to other studies,[16 17] smoking was not found to be significantly associated with stillbirth in our study population after adjusting for other risk factors (aOR = 1.26; 95% CI = 0.97 to 1.63). This could be because smoking at the time of the survey may not reflect smoking during pregnancy. While smoking during pregnancy is discouraged, healthcare messages about chewing tobacco during pregnancy are less clear.

Our findings related to timing and number of ANC visits conforms to the results of other studies. A study by Chopra et al estimated that 24% of stillbirth and perinatal deaths in South Africa could be prevented every year through improved use of ANC services.[18] However another study from South Africa found that timing of booking visit may not, in isolation, be an important determinant of stillbirth.[19] Improving the quality of pregnancy care with specific measures to prevent stillbirth are important in addition to increasing coverage of antennal care. Assisted delivery and caesarean section were found to be associated with higher risk of stillbirth, but this association was grossly attenuated and no longer statistically significant for caesarean section after adjusting for obstructed labour, suggesting the presence of a reverse causation. Similar to other studies, our study showed a U-shaped association between maternal age and stillbirth[20-22] and male sex of the fetus to be

For peer review only - http://bmjopen.bmj.com/site/about/guidelines.xhtml

associated with higher odds of stillbirth.[1 23] The higher odds of stillbirth observed with multiple pregnancies compared with singleton pregnancies can be explained by propensity of women with multiple pregnancies to have more complications.[24]

A number of studies have shown that anaemia during pregnancy is associated with increased risk of stillbirth, with 3.7-16 times higher odds of stillbirth associated with anaemia among pregnant women.[25 26] A meta-analysis of risk factors in high income countries reported higher odds of stillbirth for women with pregnancy-induced hypertension, pre-eclampsia and eclampsia.[27] Antepartum and intrapartum haemorrhage can be due to a myriad of obstetric complications, a meta-analysis reported that there was a four-fold increased risk of stillbirth associated with bleeding.[28] Breech presentation, abnormal fetal position, and obstructed labour operate through similar mechanisms, the fetus is trapped in the birth canal and is subjected to hypoxia leading to stillbirth.[29] However, a stillbirth could be prevented if these complications are detected timely and managed appropriately. Previous studies suggest that women with pre-existing mental health problems have 70% higher risk of stillbirth.[30]

The Annual Health Survey is the largest health survey in the world. The large sample provided adequate number of events (stillbirths) and allowed us to conduct an adequately powered, robust examination of a wide range of risk factors. The survey is a representative sample from nine states in India with high rates of stillbirth and the sampling strategy minimised the possibility of selection bias. The findings are generalizable for high burden states included in this study, but may not be generalizable to the rest of India. To our knowledge, no other large dataset is available to investigate stillbirth in India, the country with the highest number of stillbirths.

There are some limitations of this study which should be considered when interpreting the findings. Important to consider is that stillbirths may be under-reported or misclassified in the AHS from which our data were drawn. A study by Christou et al, 2017[31] showed that stillbirths are likely to be under-reported in routine household surveys, but this was more likely to be due a lack of rigorous ascertainment of pregnancy outcomes rather than deliberate non-reporting by women due to any reason. We did not find any evidence from published literature suggesting under-reporting of stillbirth to vary by risk factors or specific population groups, thus we excluded the possibility of differential under-reporting. However, we cannot exclude the possibility of misclassification of stillbirth as miscarriage/ abortion or neonatal deaths as stillbirth. A small proportion of the stillbirths were reported as pregnancy loss/ abortion after seven months of gestation in the dataset. We reclassified these as stillbirth in our analysis.

For peer review only - http://bmjopen.bmj.com/site/about/guidelines.xhtml

We acknowledge that household surveys are not the ideal source of data for stillbirth, but at present they are the only source of data for a majority of the countries. The reliability of the reporting of stillbirth in household surveys could be improved by including information on gestational age at stillbirth, probing questions as was done in a study by Dandona et al conducted in one state in India[9] or by using verbal autopsy,[32] but currently it is not being used in large household surveys due to cost implications.

Retrospective data collection could also have led to potential recall bias, and self-reported complications during pregnancy were not verified with hospital records. It was not possible to differentiate between pregnancy-induced hypertension and pre-existing essential hypertension. To minimise the effect of recall bias, dummy variables (e.g. anaemia) were generated wherever feasible and highly subjective symptoms (e.g. prolonged labour) were not investigated. Data were not available for a number of potential risk factors for stillbirth such as timing of stillbirth, thus we were not able to analyse the risk factors separately for antepartum and intrapartum stillbirths. An inherent limitation of routine survey data is missing information; however, the results of the analyses using three different methods to account for missing data were not substantially different.

Conclusion

Our study showed that despite several efforts being made to improve pregnancy care in India, socioeconomic disparities in stillbirth still exits and maternal and fetal complications were found to be important preventable risk factors. Another important finding was the risk associated with chewing tobacco which demonstrates an urgent need for strong public health messages to stop chewing tobacco during pregnancy in addition to the messages to stop smoking. Improving uptake of antenatal care, and timely identification and effective management of maternal and fetal complications could reduce preventable stillbirths. India has a large cadre of frontline healthcare workers or community health and nutrition workers called 'ASHAs' and 'Anganwadi workers'. They could play an important role in timely identification of danger signs through frequent interactions with pregnant women who are at a higher risk of stillbirth. In addition, ASHAs and Anganwadi workers are ideally placed to facilitate Information, Education and Communication (IEC) programmes to specifically target stigma around reporting of stillbirth. Eradicating poverty and promotion of female education are part of the global developmental agenda espoused by the Sustainable Development Goals, for which India is a signatory. This study showed that progress towards these goals could accelerate progress in preventing stillbirths.

Despite limitations, as highlighted by Lawn et al, 2010[32], household surveys remain the primary source of stillbirth data for low-and-middle income countries with more than 75% of

For peer review only - http://bmjopen.bmj.com/site/about/guidelines.xhtml

the global burden of stillbirths. AHS is one of the largest household surveys in the world conducted by the Office of the Registrar General & Census Commissioner of India and is therefore an important data source for preliminary and baseline studies for generating hypothesis for further in-depth research. Studies are needed to identify risk factors separately for antepartum and intrapartum stillbirths in India. The association between the maternal and fetal complications and stillbirth suggests that although at present, pregnant women in India are incentivised to seek antenatal care and give birth in health institutions, the quality of pregnancy care need further investigation. Currently, neither are all stillbirths recorded nor are local reviews of stillbirths conducted in the country. Targeting at-risk population groups, recording all stillbirths and conducting local reviews would be important to reduce the high burden of stillbirths in India.

REFERENCE LIST

1. Lawn JE, Blencowe H, Waiswa P, et al. Stillbirths: rates, risk factors, and acceleration towards 2030. The Lancet 2016;**387**(10018):587-603.
2. Frøen JF, Friberg IK, Lawn JE, et al. Stillbirths: progress and unfinished business. The Lancet;**387**(10018):574-86.
3. Blencowe H, Cousens S, Jassir FB, et al. National, regional, and worldwide estimates of stillbirth rates in 2015, with trends from 2000: a systematic analysis. The Lancet Global Health 2016;**4**(2):e98-e108.
4. Sharma D. India newborn action plan. International Journal of Medical Science Research and Practice 2015;**2**(2):58.
5. Di Mario S, Say L, Lincetto O. Risk Factors for Stillbirth in Developing Countries: A Systematic Review of the Literature. Sexually Transmitted Diseases 2007;**34**(7):S11-S21.
6. Ministry of Health and Family Welfare (MoHFW) GoI. National Health Portal: Janani Suraksha Yojana (JSY) Ne Delhi: National Institute of Health and Family Welfare (NIHFW), MoHFW; [cited 2017 18 December].
7. Office of Registrar General & Census Commissioner MoHA, Government of India. Annual Health Survey Report: A report on core and vital health indicators part I. 2014.
8. Pedersen AB, Mikkelsen EM, Cronin-Fenton D, et al. Missing data and multiple imputation in clinical epidemiological research. Clinical Epidemiology 2017;**9**:157.
9. Dandona R, Kumar GA, Kumar A, et al. Identification of factors associated with stillbirth in the Indian state of Bihar using verbal autopsy: A population-based study. PLOS Medicine 2017;**14**(8):e1002363.
10. McClure EM, Goldenberg RL. Stillbirth in Developing Countries: A review of causes, risk factors and prevention strategies. The journal of maternal-fetal & neonatal medicine : the official journal of the European Association of Perinatal Medicine, the Federation of Asia and Oceania Perinatal Societies, the International Society of Perinatal Obstetricians 2009;**22**(3):183-90.
11. Pincus T, Callahan LF. Associations of low formal education level and poor health status: behavioral, in addition to demographic and medical, explanations? Journal of clinical epidemiology 1994;**47**(4):355-61.
12. Penn N, Oteng-Ntim E, Oakley LL, et al. Ethnic variation in stillbirth risk and the role of maternal obesity: analysis of routine data from a London maternity unit. BMC pregnancy and childbirth 2014;**14**(1):404.

For peer review only - http://bmjopen.bmj.com/site/about/guidelines.xhtml

13. Smith GCS, Fretts RC. Stillbirth. The Lancet 2007;**370**(9600):1715-25.
14. Moffatt M. *An untouchable community in South India: Structure and consensus*: Princeton University Press, 2015.
15. Navaneetham K, Dharmalingam A. Utilization of maternal health care services in Southern India. Social science & medicine (1982) 2002;**55**(10):1849-69.
16. Gupta PC, Subramoney S. Smokeless tobacco use and risk of stillbirth: a cohort study in Mumbai, India. Epidemiology (Cambridge, Mass) 2006;**17**(1):47-51.
17. Marufu TC, Ahankari A, Coleman T, et al. Maternal smoking and the risk of still birth: systematic review and meta-analysis. BMC Public Health 2015;**15**:239.
18. Neogi SB, Negandhi P, Chopra S, et al. Risk Factors for Stillbirth: Findings from a Population-Based Case–Control Study, Haryana, India. Paediatric and perinatal epidemiology 2016;**30**(1):56-66.
19. Beauclair R, Petro G, Myer L. The association between timing of initiation of antenatal care and stillbirths: a retrospective cohort study of pregnant women in Cape Town, South Africa. BMC Pregnancy and Childbirth 2014;**14**(1):204.
20. Andersen A-MN, Wohlfahrt J, Christens P, et al. Maternal age and fetal loss: population based register linkage study. BMJ 2000;**320**(7251):1708-12.
21. Althabe F, Moore JL, Gibbons L, et al. Adverse maternal and perinatal outcomes in adolescent pregnancies: The Global Network's Maternal Newborn Health Registry study. Reproductive Health 2015;**12**(Suppl 2):S8-S8.
22. Khandait DW, Ambadekar NN, Zodpey SP, et al. Maternal age as a risk factor for stillbirth. Indian journal of public health 2000;**44**(1):28-30.
23. Mondal D, Galloway TS, Bailey TC, et al. Elevated risk of stillbirth in males: systematic review and meta-analysis of more than 30 million births. BMC Medicine 2014;**12**:220.
24. Kurdi AM, Mesleh RA, Al-Hakeem MM, et al. Multiple pregnancy and preterm labor. Saudi medical journal 2004;**25**(5):632-7.
25. Lone FW, Qureshi RN, Emanuel F. Maternal anaemia and its impact on perinatal outcome. Tropical Medicine & International Health 2004;**9**(4):486-90.
26. Engelgau MM, Sampson UK, Rabadan-Diehl C, et al. Tackling NCD in LMIC: Achievements and Lessons Learned From the NHLBI–UnitedHealth Global Health Centers of Excellence Program. Global Heart 2016;**11**(1):5-15.
27. Flenady V, Koopmans L, Middleton P, et al. Major risk factors for stillbirth in high-income countries: a systematic review and meta-analysis. Lancet 2011;**377**.
28. Ananth CV, Savitz DA. Vaginal bleeding and adverse reproductive outcomes: a meta-analysis. Paediatric and Perinatal Epidemiology 1994;**8**(1):62-78.
29. Lawn J, Shibuya K, Stein C. No cry at birth: global estimates of intrapartum stillbirths and intrapartum-related neonatal deaths. Bulletin of the World Health Organization 2005;**83**(6):409-17.
30. Gardosi J, Madurasinghe V, Williams M, et al. Maternal and fetal risk factors for stillbirth: population based study. Bmj 2013;**346**:f108.
31. Christou A, Dibley MJ, Raynes-Greenow C. Beyond counting stillbirths to understanding their determinants in low-and middle-income countries: a systematic assessment of stillbirth data availability in household surveys. Tropical Medicine & International Health 2017;**22**(3):294-311.
32. Lawn JE, Gravett MG, Nunes TM, et al. Global report on preterm birth and stillbirth (1 of 7): definitions, description of the burden and opportunities to improve data. BMC Pregnancy and Childbirth 2010;**10**(1):S1.

AUTHOR STATEMENT

For peer review only - http://bmjopen.bmj.com/site/about/guidelines.xhtml

Competing interests
No, there are no competing interests for any author.

Contribution to authorship
NA reviewed the literature, conducted the analysis and wrote the first draft of the paper; CC helped with the data analysis, and edited the paper; SSC helped in acquiring and interpreting the data, and edited the paper; AR edited the paper; UCS edited the paper; MK contributed to the interpretation and discussion of the results, and edited the paper; MN developed the concept for the study, supervised the data analysis, interpretation and discussion of the results, and edited the paper.

Details of ethics approval
Ethics approval was not required since this was a secondary analysis of anonymous survey data.

Funding
The study was funded by a Medical Research Council Career Development Award to Manisha Nair (Grant Ref: MR/P022030/1). The funder had no role in the study design, data analysis, data interpretation, or writing of the manuscript. All authors, had full access to all of the data in the study and can take responsibility for the integrity of the data and the accuracy of the data analysis.

Data sharing statement
The anonymised data is freely available through the Indian Government's Data Sharing Portal.

Figure legends
Figure 1: Flowchart showing the derivation of the final study population

Figure 2: Hypothesized relationship between the risk factors and stillbirth

Figure 3: Association between maternal age in 5 year groups and stillbirth

Figure-4: Rate of stillbirth overall and by nine states in India using the Annual Health Survey data (2010-13)

For peer review only

For peer review only - http://bmjopen.bmj.com/site/about/guidelines.xhtml

Figure 1: Flowchart showing the derivation of the final study population

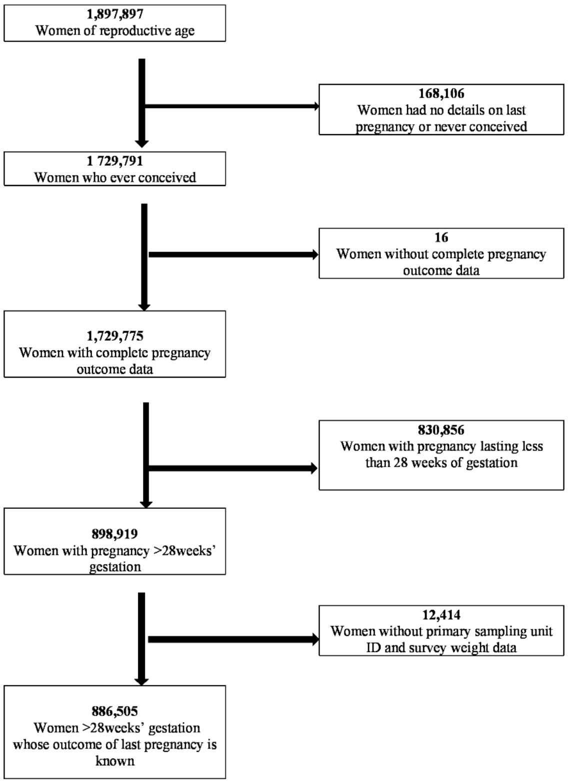

209x297mm (300 x 300 DPI)

Figure 2: Hypothesized relationship between the risk factors and stillbirth

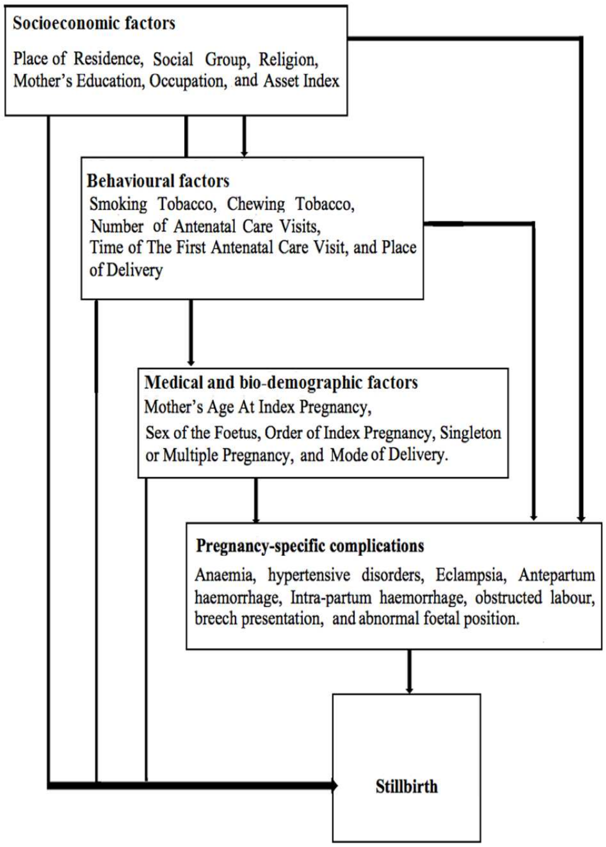

209x297mm (300 x 300 DPI)

Figure 3: Association between maternal age in 5 year groups and stillbirth

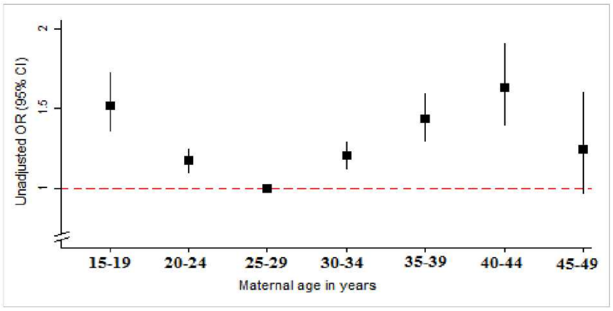

Data source: Annual Health Survey 2012-13, India

209x297mm (300 x 300 DPI)

Figure-4: Rate of stillbirth overall and by nine states in India using the Annual Health Survey data (2010-13)

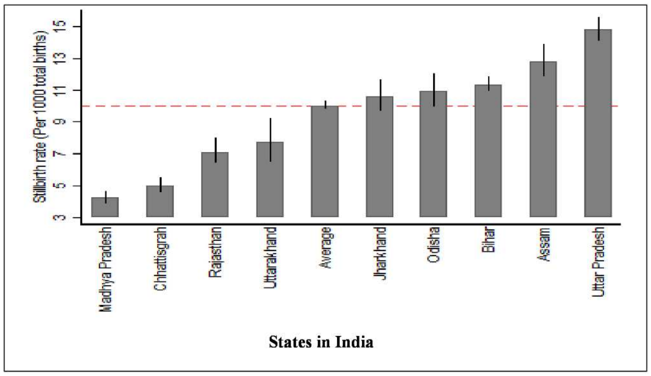

209x297mm (300 x 300 DPI)

Table-S1: Unadjusted and adjusted[1] odds ratios (OR) and 95%CI[2] for stillbirth by socioeconomic, health seeking behaviour, pre-existing medical conditions, and bio-demographic factors

Variables	Unadjusted Odds Ratios (95%CI)	Main model	Sensitivity analysis	
		Missing indicator Adjusted Odds Ratios (95%CI)	Complete case analysis Adjusted Odds Ratios (95%CI)	Multiple imputation Adjusted Odds Ratios (95%CI)
**Maternal Age (years)**				
15-19	1·52 (1·35-1·72)	1·76 (1·55-2·00)	1·58 (1·30-1·93)	1·79 (1·55 - 2·07)
20-24	1·17 (1·09-1·24)	1·29 (1·21-1·37)	1·21 (1·10-1·32)	1·29 (1·20 - 1·39)
25-29	1·00 (Ref)	1·00 (Ref)	1·00 (Ref)	1·00 (Ref)
30-34	1·20 (1·11-1·29)	1·05 (0·97-1·14)	1·13 (1·01-1·26)	1·07 (0·98 - 1·18)
35-39	1·43 (1·29-1·59)	1·16 (1·04-1·29)	1·18 (1·00-1·38)	1·18 (1·04 - 1·34)
40-45	1·63 (1·39-1·91)	1·29 (1·09-1·51)	1·42 (1·12-1·79)	1·37 (1·14 - 1·65)
45-49	1·24 (0·96-1·60)	1·04 (0·80-1·35)	1·05 (0·72-1·55)	1·05 (0·82 - 1·49)
**Place of Residence**				
Urban	1·00 (Ref)	1·00 (Ref)	1·00 (Ref)	1·00 (Ref)
Rural	1·35 (1·24-1·47)	1·27 (1·16 - 1·39)	1·31 (1·15-1·49)	1·25 (1·13 - 1·38)
**Religion**				
Hindu	1·00 (Ref)	1·00 (Ref)	1·00 (Ref)	1·00 (Ref)
Muslim	1·48 (1·39-1·58)	1·33 (1·25-1·43)	1·35 (1·22-1·49)	1·40 (1·29 - 1·51)
Christian	1·49 (1·25-1·78)	1·42 (1·19-1·70)	1·53 (1·25-1·86)	1·44 (1·20 - 1·73)
Others	0·87 (0·69-1·11)	1·06 (0·84-1·35)	0·85 (0·60-1·19)	1·09 (0·85 - 1·40)
**Social Group**				
Other	1·00 (Ref)	1·00 (Ref)	1·00 (Ref)	1·00 (Ref)
Schedule Caste	1·09 (1·02-1·16)	1·11 (1·04-1·19)	1·16 (1·05-1·28)	1·14 (1·05 - 1·23)
Schedule Tribe	0·68 (0·62-0·74)	0·74 (0·68-0·81)	0·67 (0·59-0·76)	0·71 (0·64 - 0·79)
**Maternal Education**				
Tertiary and above	1·00 (Ref)	1·00 (Ref)	1·00 (Ref)	1·00 (Ref)
Secondary	1·31 (1·08-1·60)	1·10 (0·90-1·34)	1·11 (0·84-1·47)	1·04 (0·84 - 1·30)
Primary and below	1·77 (1·48-2·12)	1·34 (1·10-1·62)	1·25 (0·95-1·64)	1·24 (1·00 - 1·53)
Illiterate	2·06 (1·72-2·47)	1·43 (1·17-1·74)	1·34 (1·02-1·77)	1·31 (1·06 - 1·62)
**Employment**				
In paid employment	1·00 (Ref)	1·00 (Ref)	1·00 (Ref)	1·00 (Ref)
Not in-paid employment	1·29 (1·20-1·38)	1·15 (1·07-1·24)	1·22 (1·10-1·36)	1·19 (1·09-1·29)
**Asset Index, Quintiles**				
5 "Highest"	1·00 (Ref)	1·00 (Ref)	1·00 (Ref)	1·00 (Ref)
4	1·26 (1·14-1·38)	1·34 (1·01-1·79)	1·58 (1·10-2·28)	1·32 (0·99-1·75)
3	1·41 (1·29-1·55)	1·91 (1·45-2·52)	2·13 (1·49-3·03)	1·81 (1·39-2·34)
2	1·46 (1·33-1·60)	2·45 (1·85-3·24)	2·64 (1·83-3·78)	2·23 (1·69-2·92)
1 "Lowest"	1·39 (1·26-1·52)	2·42 (1·82-3·21)	3·14 (2·20-4·47)	2·24 (1·71-2·94)
Missing	1·41 (1·27-1·56)	1·21 (0·91-1·62)	-	-

**Chewing Tobacco[3]**				
No	1·00 (Ref)	1·00 (Ref)	1·00 (Ref)	1·00 (Ref)
Yes	1·10 (1·01-1·19)	1·11 (1·02-1·21)	1·07 (0·97-1·19)	1·12 (1·00-1·25)
Missing	1·16 (1·09-1·24)	1·44 (1·29-1·59)	-	-
**Number of antenatal care visit**				
4 or more	1·00 (Ref)	1·00 (Ref)	1·00 (Ref)	1·00 (Ref)
Less than 4	1·19 (1·12-1·26)	1·08 (1·01-1·15)	1·11 (1·03-1·21)	1·06 (0·99-1·14)
Missing	1·53 (1·41-1·66)	1·36 (1·25-1·48)	-	-
**Timing of the first antenatal care visit**	1·00 (Ref)	-	-	-
Three or less	1·12 (1·06-1·19)	-	-	-
More than three	1·41 (1·31-1·52)	-	-	-
Missing				
**Place of delivery**				
Medical facility	1·00 (Ref)	-	-	-
Home	0·94 (0·89-0·99)	-	-	-
**Number of pregnancies**				
1	1·00 (Ref)	1·00 (Ref)	1·00 (Ref)	1·00 (Ref)
2-4	1·77 (1·63-1·93)	3·06 (2·42-3·86)	2·95 (2·20-3·94)	2·72 (2·18-3·38)
5 or more	2·60 (2·36-2·87)	4·98 (3·66-6·74)	5·35 (3·73-7·67)	4·30 (3·24-5·70)
Missing	1·21 (1·10-1·32)	1·40 (0·68-2·90)	-	-
**Sex of the baby**				
Female	1·00 (Ref)	1·00 (Ref)	1·00 (Ref)	1·00 (Ref)
Male	1·25 (1·19-1·32)	1·26 (1·20 - 1·33)	1·23 (1·14-1·32)	1·25 (1·18 - 1·32)
**Multiple gestations (index pregnancy)**	1·00 (Ref)	1·00 (Ref)	1·00 (Ref)	1·00 (Ref)
Singleton	2·03 (1·67-2·45)	1·77 (1·47 - 2·15)	1·90 (1·46-2·49)	1·84 (1·49 - 2·28)
Twin pregnancy				
**Mode of delivery**				
Spontaneous vaginal	1·00 (Ref)	1·00 (Ref)	1·00 (Ref)	1·00 (Ref)
Assisted vaginal	3·13 (2·76-3·55)	3·45 (3·02-3·93)	3·88 (3·25-4·63)	3·63 (3·16-4·18)
Caesarean section	1·53 (1·40-1·66)	1·73 (1·58-1·89)	1·75 (1·56-1·98)	1·58 (1·43-1·74)
**Any pregnancy complications**				
No	1·00 (Ref)	1·00 (Ref)	1·00 (Ref)	1·00 (Ref)
Yes	1·47 (1·38-1·56)	1·42 (1·33-1·51)	1·44 (1·33-1·56)	1·40 (1·31-1·49)
Missing	0·91 (0·84-0·98)	1·99 (1·00-3·94)	-	-

[1] Multivariable logistic regression, adjusting for the other variables in the model –except self-reported mental illness. The model also adjusts for the observed significant interaction between Gravidity and Asset Index.

[2] The 95% confidence intervals were calculated using linearized standard errors

[3] These variables reflect practice at the time of the survey

Results are weighted for design effects and non-response

Table-S2: Sensitivity analysis - Unadjusted and adjusted[1] odds ratios (95% CI[2]) for stillbirth by pregnancy complications

Variable	Unadjusted Odds ratios (95%CI)	Main Model	Sensitivity analyses	
		Missing indicator method Adjusted Odds ratios (95%CI)	Complete case analysis[2] Adjusted Odds ratios (95%CI)	Multiple imputations[2] Adjusted Odds ratios (95%CI)
**Anemia**				
No	1 (Ref)	1 (Ref)	1 (Ref)	1 (Ref)
Yes	1·45 (1·36-1·54)	1·35 (1·27-1·43)	1·41 (1·30-1·52)	1·34 (1·26-1·42)
**Eclampsia**				
No	1 (Ref)	1 (Ref)	1 (Ref)	1 (Ref)
Yes	1·90 (1·73 -2·10)	1·79 (1·62 -1·97)	1·94 (1·72 -2·19)	1·79 (1·62 -1·98)
**Other hypertensive disorders**				
No	1 (Ref)	1 (Ref)	1 (Ref)	1 (Ref)
Yes	1·20 (1·05-1·36)	1·22 (1·07-1·38)	1·25 (1·07-1·46)	1·21 (1·06-1·38)
**Intra-partum Hemorrhage**				
No	1 (Ref)	1 (Ref)	1 (Ref)	1 (Ref)
Yes	2·97 (2·75 -3·21)	2·75 (2·54 -2·97)	2·73 (2·47 -3·02)	2·77 (2·56 -3·00)
**Ante-partum Hemorrhage**				
No	1 (Ref)	1 (Ref)	1 (Ref)	1 (Ref)
Yes	1·44 (1·25-1·67)	1·29 (1·11-1·50)	1·51 (1·39-1·64)	1·32 (1·14-1·53)
**Obstructed Labor**				
No	1 (Ref)	1 (Ref)	1 (Ref)	1 (Ref)
Yes	3·35 (3·13-3·58)	3·45 (3·19-3·74)	3·57 (3·24-3·94)	3·42 (3·16-3·70)
**Breech presentation**				
No	1 (Ref)	1 (Ref)	1 (Ref)	1 (Ref)
Yes	3·18 (2·88-3·50)	2·80 (2·51-3·12)	3·04 (2·65-3·47)	2·85 (2·56-3·18)
**Abnormal Fetal position**				
No	1 (Ref)	1 (Ref)	1 (Ref)	1 (Ref)
Yes	1·72 (1·54-1·92)	1·58 (1·40-1·77)	1·66 (1·43-1·91)	1·60 (1·43-1·80)

[1]Each multivariable logistic regression model adjusts for socioeconomic, health seeking behaviour, and bio-demographic characteristics identified to be significantly associated with Stillbirth in the previous model.
[2] Although the sub-population was restricted to women who had complete data on pregnancy-specific complication, the other variables adjusted for in the model had missing data, therefore sensitivity analyses were conducted.
Results are weighted for design effects and non-response

STROBE Statement—Checklist of items that should be included in reports of ***cross-sectional studies***

	Item No	Recommendation	Page No
**Title and abstract**	1	(*a*) Indicate the study's design with a commonly used term in the title or the abstract	1,2
		(*b*) Provide in the abstract an informative and balanced summary of what was done and what was found	2
**Introduction**			
Background/rationale	2	Explain the scientific background and rationale for the investigation being reported	4
Objectives	3	State specific objectives, including any prespecified hypotheses	4
**Methods**			
Study design	4	Present key elements of study design early in the paper	4, 5
Setting	5	Describe the setting, locations, and relevant dates, including periods of recruitment, exposure, follow-up, and data collection	4,5
Participants	6	(*a*) Give the eligibility criteria, and the sources and methods of selection of participants	4,5, Figure-1
Variables	7	Clearly define all outcomes, exposures, predictors, potential confounders, and effect modifiers. Give diagnostic criteria, if applicable	5,6,7
Data sources/ measurement	8*	For each variable of interest, give sources of data and details of methods of assessment (measurement). Describe comparability of assessment methods if there is more than one group	5,6
Bias	9	Describe any efforts to address potential sources of bias	6,7
Study size	10	Explain how the study size was arrived at	7
Quantitative variables	11	Explain how quantitative variables were handled in the analyses. If applicable, describe which groupings were chosen and why	5,6,7
Statistical methods	12	(*a*) Describe all statistical methods, including those used to control for confounding	6,7
		(*b*) Describe any methods used to examine subgroups and interactions	6,7
		(*c*) Explain how missing data were addressed	7
		(*d*) If applicable, describe analytical methods taking account of sampling strategy	7
		(*e*) Describe any sensitivity analyses	7
**Results**			
Participants	13*	(a) Report numbers of individuals at each stage of study—eg numbers potentially eligible, examined for eligibility, confirmed eligible, included in the study, completing follow-up, and analysed	4 and Figure-1
		(b) Give reasons for non-participation at each stage	Figure-1
		(c) Consider use of a flow diagram	Figure-1
Descriptive data	14*	(a) Give characteristics of study participants (eg demographic, clinical, social) and information on exposures and potential confounders	8,9, 10, 11, 12
		(b) Indicate number of participants with missing data for each	10,11,12

For peer review only

		variable of interest	
Outcome data	15*	Report numbers of outcome events or summary measures	8, 9
Main results	16	(*a*) Give unadjusted estimates and, if applicable, confounder-adjusted estimates and their precision (eg, 95% confidence interval). Make clear which confounders were adjusted for and why they were included	8, 9,10,11,12, 13,14, 15
		(*b*) Report category boundaries when continuous variables were categorized	9
		(*c*) If relevant, consider translating estimates of relative risk into absolute risk for a meaningful time period	Not applicable
Other analyses	17	Report other analyses done—eg analyses of subgroups and interactions, and sensitivity analyses	Table-S1 and S2
**Discussion**			
Key results	18	Summarise key results with reference to study objectives	16
Limitations	19	Discuss limitations of the study, taking into account sources of potential bias or imprecision. Discuss both direction and magnitude of any potential bias	18, 19
Interpretation	20	Give a cautious overall interpretation of results considering objectives, limitations, multiplicity of analyses, results from similar studies, and other relevant evidence	16,17,18
Generalisability	21	Discuss the generalisability (external validity) of the study results	17,18
**Other information**			
Funding	22	Give the source of funding and the role of the funders for the present study and, if applicable, for the original study on which the present article is based	22

*Give information separately for exposed and unexposed groups.

Note: An Explanation and Elaboration article discusses each checklist item and gives methodological background and published examples of transparent reporting. The STROBE checklist is best used in conjunction with this article (freely available on the Web sites of PLoS Medicine at http://www.plosmedicine.org/, Annals of Internal Medicine at http://www.annals.org/, and Epidemiology at http://www.epidem.com/). Information on the STROBE Initiative is available at www.strobe-statement.org.

For peer review only
